# Supplementary material for: Genome-wide identification, characterization and gene expression of BES1 transcription factor family in grapevine (Vitis vinifera L.)
Source: Sci Rep. 2023 Jan 5;13:240. doi: 10.1038/s41598-022-24407-y (PMC9816167; doi:10.1038/s41598-022-24407-y)
Supplement: Supplementary file 3 — Supplementary Information. [file 41598_2022_24407_MOESM3_ESM.zip › Chromosome collinearity analysis/MCsanX.out.html.zip!/MCsanX.out.html/8.html]

|  |  |  |  |  |  |  |  |  |  |  |  |  |  |
| --- | --- | --- | --- | --- | --- | --- | --- | --- | --- | --- | --- | --- | --- |
| Duplication depth | Reference chromosome | Collinear blocks | | | | | | | | | | | |
| 0 | transcript:Vitvi08g04000\_t001 |  |  |  |  |  |  |  |  |  |  |  |  |
| 0 | transcript:Vitvi08g04001\_t001 |  |  |  |  |  |  |  |  |  |  |  |  |
| 0 | transcript:Vitvi08g00004\_t002 |  |  |  |  |  |  |  |  |  |  |  |  |
| 0 | transcript:Vitvi08g00005\_t001 |  |  |  |  |  |  |  |  |  |  |  |  |
| 0 | transcript:Vitvi08g04002\_t001 |  |  |  |  |  |  |  |  |  |  |  |  |
| 0 | transcript:Vitvi08g04003\_t001 |  |  |  |  |  |  |  |  |  |  |  |  |
| 0 | transcript:Vitvi08g00008\_t001 |  |  |  |  |  |  |  |  |  |  |  |  |
| 0 | transcript:Vitvi08g00009\_t001 |  |  |  |  |  |  |  |  |  |  |  |  |
| 0 | transcript:Vitvi08g00010\_t001 |  |  |  |  |  |  |  |  |  |  |  |  |
| 0 | transcript:Vitvi08g00011\_t001 |  |  |  |  |  |  |  |  |  |  |  |  |
| 0 | transcript:Vitvi08g00012\_t001 |  |  |  |  |  |  |  |  |  |  |  |  |
| 0 | transcript:Vitvi08g00013\_t002 |  |  |  |  |  |  |  |  |  |  |  |  |
| 0 | transcript:Vitvi08g00014\_t001 |  |  |  |  |  |  |  |  |  |  |  |  |
| 0 | transcript:Vitvi08g01970\_t001 |  |  |  |  |  |  |  |  |  |  |  |  |
| 0 | transcript:Vitvi08g00015\_t001 |  |  |  |  |  |  |  |  |  |  |  |  |
| 0 | transcript:Vitvi08g01971\_t001 |  |  |  |  |  |  |  |  |  |  |  |  |
| 0 | transcript:Vitvi08g00016\_t001 |  |  |  |  |  |  |  |  |  |  |  |  |
| 0 | transcript:Vitvi08g00017\_t001 |  |  |  |  |  |  |  |  |  |  |  |  |
| 0 | transcript:Vitvi08g00018\_t001 |  |  |  |  |  |  |  |  |  |  |  |  |
| 0 | transcript:Vitvi08g00019\_t001 |  |  |  |  |  |  |  |  |  |  |  |  |
| 0 | transcript:Vitvi08g00020\_t001 |  |  |  |  |  |  |  |  |  |  |  |  |
| 0 | transcript:Vitvi08g00021\_t001 |  |  |  |  |  |  |  |  |  |  |  |  |
| 0 | transcript:Vitvi08g00022\_t001 |  |  |  |  |  |  |  |  |  |  |  |  |
| 0 | transcript:Vitvi08g00023\_t001 |  |  |  |  |  |  |  |  |  |  |  |  |
| 0 | transcript:Vitvi08g01973\_t001 |  |  |  |  |  |  |  |  |  |  |  |  |
| 0 | transcript:Vitvi08g00024\_t002 |  |  |  |  |  |  |  |  |  |  |  |  |
| 0 | transcript:Vitvi08g00026\_t001 |  |  |  |  |  |  |  |  |  |  |  |  |
| 0 | transcript:Vitvi08g01974\_t001 |  |  |  |  |  |  |  |  |  |  |  |  |
| 0 | transcript:Vitvi08g00027\_t001 |  |  |  |  |  |  |  |  |  |  |  |  |
| 0 | transcript:Vitvi08g00028\_t002 |  |  |  |  |  |  |  |  |  |  |  |  |
| 0 | transcript:Vitvi08g00030\_t001 |  |  |  |  |  |  |  |  |  |  |  |  |
| 0 | transcript:Vitvi08g04004\_t001 |  |  |  |  |  |  |  |  |  |  |  |  |
| 0 | transcript:Vitvi08g00033\_t001 |  |  |  |  |  |  |  |  |  |  |  |  |
| 0 | transcript:Vitvi08g04005\_t001 |  |  |  |  |  |  |  |  |  |  |  |  |
| 0 | transcript:Vitvi08g04006\_t001 |  |  |  |  |  |  |  |  |  |  |  |  |
| 0 | transcript:Vitvi08g04007\_t001 |  |  |  |  |  |  |  |  |  |  |  |  |
| 0 | transcript:Vitvi08g00037\_t001 |  |  |  |  |  |  |  |  |  |  |  |  |
| 0 | transcript:Vitvi08g00038\_t001 |  |  |  |  |  |  |  |  |  |  |  |  |
| 0 | transcript:Vitvi08g00039\_t001 |  |  |  |  |  |  |  |  |  |  |  |  |
| 0 | transcript:Vitvi08g00041\_t001 |  |  |  |  |  |  |  |  |  |  |  |  |
| 0 | transcript:Vitvi08g00042\_t001 |  |  |  |  |  |  |  |  |  |  |  |  |
| 0 | transcript:Vitvi08g04008\_t001 |  |  |  |  |  |  |  |  |  |  |  |  |
| 0 | transcript:Vitvi08g00045\_t001 |  |  |  |  |  |  |  |  |  |  |  |  |
| 0 | transcript:Vitvi08g00046\_t002 |  |  |  |  |  |  |  |  |  |  |  |  |
| 0 | transcript:Vitvi08g04009\_t001 |  |  |  |  |  |  |  |  |  |  |  |  |
| 0 | transcript:Vitvi08g01983\_t001 |  |  |  |  |  |  |  |  |  |  |  |  |
| 0 | transcript:Vitvi08g00047\_t001 |  |  |  |  |  |  |  |  |  |  |  |  |
| 0 | transcript:Vitvi08g00048\_t001 |  |  |  |  |  |  |  |  |  |  |  |  |
| 0 | transcript:Vitvi08g04010\_t001 |  |  |  |  |  |  |  |  |  |  |  |  |
| 0 | transcript:Vitvi08g00050\_t001 |  |  |  |  |  |  |  |  |  |  |  |  |
| 0 | transcript:Vitvi08g00051\_t002 |  |  |  |  |  |  |  |  |  |  |  |  |
| 0 | transcript:Vitvi08g00052\_t001 |  |  |  |  |  |  |  |  |  |  |  |  |
| 0 | transcript:Vitvi08g00053\_t001 |  |  |  |  |  |  |  |  |  |  |  |  |
| 0 | transcript:Vitvi08g00054\_t001 |  |  |  |  |  |  |  |  |  |  |  |  |
| 0 | transcript:Vitvi08g00055\_t001 |  |  |  |  |  |  |  |  |  |  |  |  |
| 0 | transcript:Vitvi08g04011\_t001 |  |  |  |  |  |  |  |  |  |  |  |  |
| 0 | transcript:Vitvi08g00056\_t001 |  |  |  |  |  |  |  |  |  |  |  |  |
| 0 | transcript:Vitvi08g01987\_t005 |  |  |  |  |  |  |  |  |  |  |  |  |
| 0 | transcript:Vitvi08g00058\_t001 |  |  |  |  |  |  |  |  |  |  |  |  |
| 0 | transcript:Vitvi08g00059\_t001 |  |  |  |  |  |  |  |  |  |  |  |  |
| 0 | transcript:Vitvi08g00060\_t001 |  |  |  |  |  |  |  |  |  |  |  |  |
| 0 | transcript:Vitvi08g00061\_t002 |  |  |  |  |  |  |  |  |  |  |  |  |
| 0 | transcript:Vitvi08g00062\_t002 |  |  |  |  |  |  |  |  |  |  |  |  |
| 0 | transcript:Vitvi08g01988\_t001 |  |  |  |  |  |  |  |  |  |  |  |  |
| 0 | transcript:Vitvi08g00064\_t001 |  |  |  |  |  |  |  |  |  |  |  |  |
| 0 | transcript:Vitvi08g04012\_t001 |  |  |  |  |  |  |  |  |  |  |  |  |
| 0 | transcript:Vitvi08g00067\_t001 |  |  |  |  |  |  |  |  |  |  |  |  |
| 0 | transcript:Vitvi08g00068\_t002 |  |  |  |  |  |  |  |  |  |  |  |  |
| 0 | transcript:Vitvi08g00069\_t001 |  |  |  |  |  |  |  |  |  |  |  |  |
| 0 | transcript:Vitvi08g00070\_t001 |  |  |  |  |  |  |  |  |  |  |  |  |
| 0 | transcript:Vitvi08g00071\_t001 |  |  |  |  |  |  |  |  |  |  |  |  |
| 0 | transcript:Vitvi08g00072\_t001 |  |  |  |  |  |  |  |  |  |  |  |  |
| 0 | transcript:Vitvi08g04013\_t001 |  |  |  |  |  |  |  |  |  |  |  |  |
| 0 | transcript:Vitvi08g04014\_t001 |  |  |  |  |  |  |  |  |  |  |  |  |
| 0 | transcript:Vitvi08g04015\_t001 |  |  |  |  |  |  |  |  |  |  |  |  |
| 0 | transcript:Vitvi08g00076\_t001 |  |  |  |  |  |  |  |  |  |  |  |  |
| 0 | transcript:Vitvi08g01990\_t001 |  |  |  |  |  |  |  |  |  |  |  |  |
| 0 | transcript:Vitvi08g04016\_t001 |  |  |  |  |  |  |  |  |  |  |  |  |
| 0 | transcript:Vitvi08g00078\_t001 |  |  |  |  |  |  |  |  |  |  |  |  |
| 0 | transcript:Vitvi08g04017\_t001 |  |  |  |  |  |  |  |  |  |  |  |  |
| 0 | transcript:Vitvi08g00079\_t001 |  |  |  |  |  |  |  |  |  |  |  |  |
| 0 | transcript:Vitvi08g00083\_t001 |  |  |  |  |  |  |  |  |  |  |  |  |
| 0 | transcript:Vitvi08g00085\_t002 |  |  |  |  |  |  |  |  |  |  |  |  |
| 0 | transcript:Vitvi08g01991\_t001 |  |  |  |  |  |  |  |  |  |  |  |  |
| 0 | transcript:Vitvi08g04018\_t001 |  |  |  |  |  |  |  |  |  |  |  |  |
| 0 | transcript:Vitvi08g00086\_t001 |  |  |  |  |  |  |  |  |  |  |  |  |
| 0 | transcript:Vitvi08g04019\_t001 |  |  |  |  |  |  |  |  |  |  |  |  |
| 0 | transcript:Vitvi08g00090\_t001 |  |  |  |  |  |  |  |  |  |  |  |  |
| 0 | transcript:Vitvi08g04020\_t001 |  |  |  |  |  |  |  |  |  |  |  |  |
| 0 | transcript:Vitvi08g00091\_t001 |  |  |  |  |  |  |  |  |  |  |  |  |
| 0 | transcript:Vitvi08g04021\_t001 |  |  |  |  |  |  |  |  |  |  |  |  |
| 0 | transcript:Vitvi08g00092\_t001 |  |  |  |  |  |  |  |  |  |  |  |  |
| 0 | transcript:Vitvi08g00093\_t001 |  |  |  |  |  |  |  |  |  |  |  |  |
| 0 | transcript:Vitvi08g00094\_t001 |  |  |  |  |  |  |  |  |  |  |  |  |
| 0 | transcript:Vitvi08g04022\_t001 |  |  |  |  |  |  |  |  |  |  |  |  |
| 0 | transcript:Vitvi08g00095\_t001 |  |  |  |  |  |  |  |  |  |  |  |  |
| 0 | transcript:Vitvi08g00096\_t001 |  |  |  |  |  |  |  |  |  |  |  |  |
| 0 | transcript:Vitvi08g00097\_t001 |  |  |  |  |  |  |  |  |  |  |  |  |
| 0 | transcript:Vitvi08g00099\_t003 |  |  |  |  |  |  |  |  |  |  |  |  |
| 0 | transcript:Vitvi08g00100\_t001 |  |  |  |  |  |  |  |  |  |  |  |  |
| 0 | transcript:Vitvi08g00101\_t001 |  |  |  |  |  |  |  |  |  |  |  |  |
| 0 | transcript:Vitvi08g00102\_t001 |  |  |  |  |  |  |  |  |  |  |  |  |
| 0 | transcript:Vitvi08g00103\_t001 |  |  |  |  |  |  |  |  |  |  |  |  |
| 0 | transcript:Vitvi08g00104\_t001 |  |  |  |  |  |  |  |  |  |  |  |  |
| 0 | transcript:Vitvi08g00105\_t001 |  |  |  |  |  |  |  |  |  |  |  |  |
| 0 | transcript:Vitvi08g00107\_t001 |  |  |  |  |  |  |  |  |  |  |  |  |
| 0 | transcript:Vitvi08g00108\_t001 |  |  |  |  |  |  |  |  |  |  |  |  |
| 0 | transcript:Vitvi08g00110\_t001 |  |  |  |  |  |  |  |  |  |  |  |  |
| 0 | transcript:Vitvi08g01993\_t001 |  |  |  |  |  |  |  |  |  |  |  |  |
| 0 | transcript:Vitvi08g00112\_t001 |  |  |  |  |  |  |  |  |  |  |  |  |
| 0 | transcript:Vitvi08g01994\_t001 |  |  |  |  |  |  |  |  |  |  |  |  |
| 0 | transcript:Vitvi08g00113\_t001 |  |  |  |  |  |  |  |  |  |  |  |  |
| 0 | transcript:Vitvi08g00114\_t001 |  |  |  |  |  |  |  |  |  |  |  |  |
| 0 | transcript:Vitvi08g00115\_t001 |  |  |  |  |  |  |  |  |  |  |  |  |
| 0 | transcript:Vitvi08g00117\_t001 |  |  |  |  |  |  |  |  |  |  |  |  |
| 0 | transcript:Vitvi08g00118\_t001 |  |  |  |  |  |  |  |  |  |  |  |  |
| 0 | transcript:Vitvi08g00120\_t001 |  |  |  |  |  |  |  |  |  |  |  |  |
| 0 | transcript:Vitvi08g01995\_t001 |  |  |  |  |  |  |  |  |  |  |  |  |
| 0 | transcript:Vitvi08g04023\_t001 |  |  |  |  |  |  |  |  |  |  |  |  |
| 0 | transcript:Vitvi08g00125\_t001 |  |  |  |  |  |  |  |  |  |  |  |  |
| 0 | transcript:Vitvi08g00126\_t001 |  |  |  |  |  |  |  |  |  |  |  |  |
| 0 | transcript:Vitvi08g01999\_t001 |  |  |  |  |  |  |  |  |  |  |  |  |
| 0 | transcript:Vitvi08g00128\_t001 |  |  |  |  |  |  |  |  |  |  |  |  |
| 0 | transcript:Vitvi08g02000\_t001 |  |  |  |  |  |  |  |  |  |  |  |  |
| 0 | transcript:Vitvi08g00129\_t001.1.6037826f |  |  |  |  |  |  |  |  |  |  |  |  |
| 0 | transcript:Vitvi08g00130\_t001 |  |  |  |  |  |  |  |  |  |  |  |  |
| 0 | transcript:Vitvi08g00132\_t001 |  |  |  |  |  |  |  |  |  |  |  |  |
| 0 | transcript:Vitvi08g00133\_t001 |  |  |  |  |  |  |  |  |  |  |  |  |
| 0 | transcript:Vitvi08g00134\_t001 |  |  |  |  |  |  |  |  |  |  |  |  |
| 0 | transcript:Vitvi08g04024\_t001 |  |  |  |  |  |  |  |  |  |  |  |  |
| 0 | transcript:Vitvi08g00135\_t003 |  |  |  |  |  |  |  |  |  |  |  |  |
| 0 | transcript:Vitvi08g04025\_t001 |  |  |  |  |  |  |  |  |  |  |  |  |
| 0 | transcript:Vitvi08g04026\_t001 |  |  |  |  |  |  |  |  |  |  |  |  |
| 0 | transcript:Vitvi08g00137\_t001 |  |  |  |  |  |  |  |  |  |  |  |  |
| 0 | transcript:Vitvi08g00138\_t001 |  |  |  |  |  |  |  |  |  |  |  |  |
| 0 | transcript:Vitvi08g04027\_t001 |  |  |  |  |  |  |  |  |  |  |  |  |
| 0 | transcript:Vitvi08g00140\_t001 |  |  |  |  |  |  |  |  |  |  |  |  |
| 0 | transcript:Vitvi08g00142\_t001 |  |  |  |  |  |  |  |  |  |  |  |  |
| 0 | transcript:Vitvi08g04028\_t001 |  |  |  |  |  |  |  |  |  |  |  |  |
| 0 | transcript:Vitvi08g00144\_t001 |  |  |  |  |  |  |  |  |  |  |  |  |
| 0 | transcript:Vitvi08g00145\_t001 |  |  |  |  |  |  |  |  |  |  |  |  |
| 0 | transcript:Vitvi08g00146\_t001 |  |  |  |  |  |  |  |  |  |  |  |  |
| 0 | transcript:Vitvi08g00148\_t001 |  |  |  |  |  |  |  |  |  |  |  |  |
| 0 | transcript:Vitvi08g00151\_t001 |  |  |  |  |  |  |  |  |  |  |  |  |
| 0 | transcript:Vitvi08g00152\_t001 |  |  |  |  |  |  |  |  |  |  |  |  |
| 0 | transcript:Vitvi08g00153\_t001 |  |  |  |  |  |  |  |  |  |  |  |  |
| 0 | transcript:Vitvi08g02002\_t001 |  |  |  |  |  |  |  |  |  |  |  |  |
| 0 | transcript:Vitvi08g00155\_t001 |  |  |  |  |  |  |  |  |  |  |  |  |
| 0 | transcript:Vitvi08g04029\_t001 |  |  |  |  |  |  |  |  |  |  |  |  |
| 0 | transcript:Vitvi08g00157\_t001 |  |  |  |  |  |  |  |  |  |  |  |  |
| 0 | transcript:Vitvi08g00159\_t001 |  |  |  |  |  |  |  |  |  |  |  |  |
| 0 | transcript:Vitvi08g02003\_t001 |  |  |  |  |  |  |  |  |  |  |  |  |
| 0 | transcript:Vitvi08g00160\_t001 |  |  |  |  |  |  |  |  |  |  |  |  |
| 0 | transcript:Vitvi08g00161\_t001 |  |  |  |  |  |  |  |  |  |  |  |  |
| 0 | transcript:Vitvi08g00163\_t001 |  |  |  |  |  |  |  |  |  |  |  |  |
| 0 | transcript:Vitvi08g02004\_t001 |  |  |  |  |  |  |  |  |  |  |  |  |
| 0 | transcript:Vitvi08g02006\_t001 |  |  |  |  |  |  |  |  |  |  |  |  |
| 0 | transcript:Vitvi08g02007\_t001 |  |  |  |  |  |  |  |  |  |  |  |  |
| 0 | transcript:Vitvi08g02008\_t001 |  |  |  |  |  |  |  |  |  |  |  |  |
| 0 | transcript:Vitvi08g04030\_t001 |  |  |  |  |  |  |  |  |  |  |  |  |
| 0 | transcript:Vitvi08g00169\_t001 |  |  |  |  |  |  |  |  |  |  |  |  |
| 0 | transcript:Vitvi08g04031\_t001 |  |  |  |  |  |  |  |  |  |  |  |  |
| 0 | transcript:Vitvi08g00171\_t001 |  |  |  |  |  |  |  |  |  |  |  |  |
| 0 | transcript:Vitvi08g04032\_t001 |  |  |  |  |  |  |  |  |  |  |  |  |
| 0 | transcript:Vitvi08g00172\_t001 |  |  |  |  |  |  |  |  |  |  |  |  |
| 0 | transcript:Vitvi08g00173\_t001 |  |  |  |  |  |  |  |  |  |  |  |  |
| 0 | transcript:Vitvi08g04033\_t001 |  |  |  |  |  |  |  |  |  |  |  |  |
| 0 | transcript:Vitvi08g00174\_t002 |  |  |  |  |  |  |  |  |  |  |  |  |
| 0 | transcript:Vitvi08g00175\_t001 |  |  |  |  |  |  |  |  |  |  |  |  |
| 0 | transcript:Vitvi08g04034\_t002 |  |  |  |  |  |  |  |  |  |  |  |  |
| 0 | transcript:Vitvi08g00177\_t001 |  |  |  |  |  |  |  |  |  |  |  |  |
| 0 | transcript:Vitvi08g00178\_t001 |  |  |  |  |  |  |  |  |  |  |  |  |
| 0 | transcript:Vitvi08g04035\_t001 |  |  |  |  |  |  |  |  |  |  |  |  |
| 0 | transcript:Vitvi08g00180\_t001 |  |  |  |  |  |  |  |  |  |  |  |  |
| 0 | transcript:Vitvi08g00182\_t001 |  |  |  |  |  |  |  |  |  |  |  |  |
| 0 | transcript:Vitvi08g02016\_t001 |  |  |  |  |  |  |  |  |  |  |  |  |
| 0 | transcript:Vitvi08g02017\_t001 |  |  |  |  |  |  |  |  |  |  |  |  |
| 0 | transcript:Vitvi08g04036\_t001 |  |  |  |  |  |  |  |  |  |  |  |  |
| 0 | transcript:Vitvi08g00184\_t001 |  |  |  |  |  |  |  |  |  |  |  |  |
| 0 | transcript:Vitvi08g00185\_t001 |  |  |  |  |  |  |  |  |  |  |  |  |
| 0 | transcript:Vitvi08g00186\_t001 |  |  |  |  |  |  |  |  |  |  |  |  |
| 0 | transcript:Vitvi08g00187\_t001 |  |  |  |  |  |  |  |  |  |  |  |  |
| 0 | transcript:Vitvi08g00188\_t001 |  |  |  |  |  |  |  |  |  |  |  |  |
| 0 | transcript:Vitvi08g00189\_t003 |  |  |  |  |  |  |  |  |  |  |  |  |
| 0 | transcript:Vitvi08g04037\_t001 |  |  |  |  |  |  |  |  |  |  |  |  |
| 0 | transcript:Vitvi08g04038\_t001 |  |  |  |  |  |  |  |  |  |  |  |  |
| 0 | transcript:Vitvi08g04039\_t001 |  |  |  |  |  |  |  |  |  |  |  |  |
| 0 | transcript:Vitvi08g04040\_t001 |  |  |  |  |  |  |  |  |  |  |  |  |
| 0 | transcript:Vitvi08g04041\_t001 |  |  |  |  |  |  |  |  |  |  |  |  |
| 0 | transcript:Vitvi08g00192\_t001 |  |  |  |  |  |  |  |  |  |  |  |  |
| 0 | transcript:Vitvi08g00193\_t001 |  |  |  |  |  |  |  |  |  |  |  |  |
| 0 | transcript:Vitvi08g00195\_t001 |  |  |  |  |  |  |  |  |  |  |  |  |
| 0 | transcript:Vitvi08g04042\_t001 |  |  |  |  |  |  |  |  |  |  |  |  |
| 0 | transcript:Vitvi08g04043\_t001 |  |  |  |  |  |  |  |  |  |  |  |  |
| 0 | transcript:Vitvi08g04044\_t001 |  |  |  |  |  |  |  |  |  |  |  |  |
| 0 | transcript:Vitvi08g04045\_t001 |  |  |  |  |  |  |  |  |  |  |  |  |
| 0 | transcript:Vitvi08g00197\_t001 |  |  |  |  |  |  |  |  |  |  |  |  |
| 0 | transcript:Vitvi08g00198\_t001 |  |  |  |  |  |  |  |  |  |  |  |  |
| 0 | transcript:Vitvi08g02021\_t001 |  |  |  |  |  |  |  |  |  |  |  |  |
| 0 | transcript:Vitvi08g04046\_t001 |  |  |  |  |  |  |  |  |  |  |  |  |
| 0 | transcript:Vitvi08g04047\_t001 |  |  |  |  |  |  |  |  |  |  |  |  |
| 0 | transcript:Vitvi08g00200\_t001 |  |  |  |  |  |  |  |  |  |  |  |  |
| 0 | transcript:Vitvi08g04048\_t001 |  |  |  |  |  |  |  |  |  |  |  |  |
| 0 | transcript:Vitvi08g04049\_t001 |  |  |  |  |  |  |  |  |  |  |  |  |
| 0 | transcript:Vitvi08g04050\_t001 |  |  |  |  |  |  |  |  |  |  |  |  |
| 0 | transcript:Vitvi08g00201\_t001 |  |  |  |  |  |  |  |  |  |  |  |  |
| 0 | transcript:Vitvi08g00202\_t001 |  |  |  |  |  |  |  |  |  |  |  |  |
| 0 | transcript:Vitvi08g04051\_t001 |  |  |  |  |  |  |  |  |  |  |  |  |
| 0 | transcript:Vitvi08g04052\_t001 |  |  |  |  |  |  |  |  |  |  |  |  |
| 0 | transcript:Vitvi08g04053\_t001 |  |  |  |  |  |  |  |  |  |  |  |  |
| 0 | transcript:Vitvi08g00203\_t002 |  |  |  |  |  |  |  |  |  |  |  |  |
| 0 | transcript:Vitvi08g00205\_t001 |  |  |  |  |  |  |  |  |  |  |  |  |
| 0 | transcript:Vitvi08g00207\_t001 |  |  |  |  |  |  |  |  |  |  |  |  |
| 0 | transcript:Vitvi08g00209\_t001 |  |  |  |  |  |  |  |  |  |  |  |  |
| 0 | transcript:Vitvi08g00212\_t001 |  |  |  |  |  |  |  |  |  |  |  |  |
| 0 | transcript:Vitvi08g00213\_t001 |  |  |  |  |  |  |  |  |  |  |  |  |
| 0 | transcript:Vitvi08g00214\_t001 |  |  |  |  |  |  |  |  |  |  |  |  |
| 0 | transcript:Vitvi08g00215\_t001 |  |  |  |  |  |  |  |  |  |  |  |  |
| 0 | transcript:Vitvi08g00216\_t001 |  |  |  |  |  |  |  |  |  |  |  |  |
| 0 | transcript:Vitvi08g00217\_t001 |  |  |  |  |  |  |  |  |  |  |  |  |
| 0 | transcript:Vitvi08g04054\_t001 |  |  |  |  |  |  |  |  |  |  |  |  |
| 0 | transcript:Vitvi08g00219\_t001 |  |  |  |  |  |  |  |  |  |  |  |  |
| 0 | transcript:Vitvi08g02028\_t001 |  |  |  |  |  |  |  |  |  |  |  |  |
| 0 | transcript:Vitvi08g04055\_t001 |  |  |  |  |  |  |  |  |  |  |  |  |
| 0 | transcript:Vitvi08g00221\_t001 |  |  |  |  |  |  |  |  |  |  |  |  |
| 0 | transcript:Vitvi08g04056\_t001 |  |  |  |  |  |  |  |  |  |  |  |  |
| 0 | transcript:Vitvi08g00222\_t001 |  |  |  |  |  |  |  |  |  |  |  |  |
| 0 | transcript:Vitvi08g00223\_t001 |  |  |  |  |  |  |  |  |  |  |  |  |
| 0 | transcript:Vitvi08g02030\_t001 |  |  |  |  |  |  |  |  |  |  |  |  |
| 0 | transcript:Vitvi08g04057\_t001 |  |  |  |  |  |  |  |  |  |  |  |  |
| 0 | transcript:Vitvi08g04058\_t001 |  |  |  |  |  |  |  |  |  |  |  |  |
| 0 | transcript:Vitvi08g00224\_t001 |  |  |  |  |  |  |  |  |  |  |  |  |
| 0 | transcript:Vitvi08g02032\_t001 |  |  |  |  |  |  |  |  |  |  |  |  |
| 0 | transcript:Vitvi08g04059\_t001 |  |  |  |  |  |  |  |  |  |  |  |  |
| 0 | transcript:Vitvi08g00225\_t001 |  |  |  |  |  |  |  |  |  |  |  |  |
| 0 | transcript:Vitvi08g04060\_t001 |  |  |  |  |  |  |  |  |  |  |  |  |
| 0 | transcript:Vitvi08g00227\_t001 |  |  |  |  |  |  |  |  |  |  |  |  |
| 0 | transcript:Vitvi08g00230\_t001 |  |  |  |  |  |  |  |  |  |  |  |  |
| 0 | transcript:Vitvi08g00233\_t001 |  |  |  |  |  |  |  |  |  |  |  |  |
| 0 | transcript:Vitvi08g00234\_t001 |  |  |  |  |  |  |  |  |  |  |  |  |
| 0 | transcript:Vitvi08g00235\_t003 |  |  |  |  |  |  |  |  |  |  |  |  |
| 0 | transcript:Vitvi08g00238\_t001 |  |  |  |  |  |  |  |  |  |  |  |  |
| 0 | transcript:Vitvi08g00239\_t001 |  |  |  |  |  |  |  |  |  |  |  |  |
| 0 | transcript:Vitvi08g04061\_t001 |  |  |  |  |  |  |  |  |  |  |  |  |
| 0 | transcript:Vitvi08g00240\_t001 |  |  |  |  |  |  |  |  |  |  |  |  |
| 0 | transcript:Vitvi08g02035\_t001 |  |  |  |  |  |  |  |  |  |  |  |  |
| 0 | transcript:Vitvi08g04062\_t001 |  |  |  |  |  |  |  |  |  |  |  |  |
| 0 | transcript:Vitvi08g00241\_t001 |  |  |  |  |  |  |  |  |  |  |  |  |
| 0 | transcript:Vitvi08g00243\_t001 |  |  |  |  |  |  |  |  |  |  |  |  |
| 0 | transcript:Vitvi08g04063\_t001 |  |  |  |  |  |  |  |  |  |  |  |  |
| 0 | transcript:Vitvi08g02036\_t001 |  |  |  |  |  |  |  |  |  |  |  |  |
| 0 | transcript:Vitvi08g00245\_t001 |  |  |  |  |  |  |  |  |  |  |  |  |
| 0 | transcript:Vitvi08g04064\_t001 |  |  |  |  |  |  |  |  |  |  |  |  |
| 0 | transcript:Vitvi08g04065\_t001 |  |  |  |  |  |  |  |  |  |  |  |  |
| 0 | transcript:Vitvi08g04066\_t001 |  |  |  |  |  |  |  |  |  |  |  |  |
| 0 | transcript:Vitvi08g04067\_t001 |  |  |  |  |  |  |  |  |  |  |  |  |
| 0 | transcript:Vitvi08g01967\_t001 |  |  |  |  |  |  |  |  |  |  |  |  |
| 0 | transcript:Vitvi08g04068\_t001 |  |  |  |  |  |  |  |  |  |  |  |  |
| 0 | transcript:Vitvi08g00256\_t001 |  |  |  |  |  |  |  |  |  |  |  |  |
| 0 | transcript:Vitvi08g00258\_t001 |  |  |  |  |  |  |  |  |  |  |  |  |
| 0 | transcript:Vitvi08g00259\_t001 |  |  |  |  |  |  |  |  |  |  |  |  |
| 0 | transcript:Vitvi08g04069\_t001 |  |  |  |  |  |  |  |  |  |  |  |  |
| 0 | transcript:Vitvi08g02039\_t001 |  |  |  |  |  |  |  |  |  |  |  |  |
| 0 | transcript:Vitvi08g00261\_t001 |  |  |  |  |  |  |  |  |  |  |  |  |
| 0 | transcript:Vitvi08g00262\_t001 |  |  |  |  |  |  |  |  |  |  |  |  |
| 0 | transcript:Vitvi08g04070\_t001 |  |  |  |  |  |  |  |  |  |  |  |  |
| 0 | transcript:Vitvi08g00263\_t001 |  |  |  |  |  |  |  |  |  |  |  |  |
| 0 | transcript:Vitvi08g04071\_t001 |  |  |  |  |  |  |  |  |  |  |  |  |
| 0 | transcript:Vitvi08g04072\_t001 |  |  |  |  |  |  |  |  |  |  |  |  |
| 0 | transcript:Vitvi08g00265\_t001 |  |  |  |  |  |  |  |  |  |  |  |  |
| 0 | transcript:Vitvi08g04073\_t001 |  |  |  |  |  |  |  |  |  |  |  |  |
| 0 | transcript:Vitvi08g04074\_t001 |  |  |  |  |  |  |  |  |  |  |  |  |
| 0 | transcript:Vitvi08g04075\_t001 |  |  |  |  |  |  |  |  |  |  |  |  |
| 0 | transcript:Vitvi08g00271\_t002 |  |  |  |  |  |  |  |  |  |  |  |  |
| 0 | transcript:Vitvi08g00272\_t001 |  |  |  |  |  |  |  |  |  |  |  |  |
| 0 | transcript:Vitvi08g00274\_t001 |  |  |  |  |  |  |  |  |  |  |  |  |
| 0 | transcript:Vitvi08g04076\_t001 |  |  |  |  |  |  |  |  |  |  |  |  |
| 0 | transcript:Vitvi08g02044\_t001 |  |  |  |  |  |  |  |  |  |  |  |  |
| 0 | transcript:Vitvi08g00281\_t001 |  |  |  |  |  |  |  |  |  |  |  |  |
| 0 | transcript:Vitvi08g00282\_t001 |  |  |  |  |  |  |  |  |  |  |  |  |
| 0 | transcript:Vitvi08g00283\_t001 |  |  |  |  |  |  |  |  |  |  |  |  |
| 0 | transcript:Vitvi08g02046\_t001 |  |  |  |  |  |  |  |  |  |  |  |  |
| 0 | transcript:Vitvi08g00289\_t002 |  |  |  |  |  |  |  |  |  |  |  |  |
| 0 | transcript:Vitvi08g00290\_t001 |  |  |  |  |  |  |  |  |  |  |  |  |
| 0 | transcript:Vitvi08g00291\_t001 |  |  |  |  |  |  |  |  |  |  |  |  |
| 0 | transcript:Vitvi08g04077\_t001 |  |  |  |  |  |  |  |  |  |  |  |  |
| 0 | transcript:Vitvi08g00292\_t001 |  |  |  |  |  |  |  |  |  |  |  |  |
| 0 | transcript:Vitvi08g00294\_t001 |  |  |  |  |  |  |  |  |  |  |  |  |
| 0 | transcript:Vitvi08g00297\_t001 |  |  |  |  |  |  |  |  |  |  |  |  |
| 0 | transcript:Vitvi08g00298\_t001 |  |  |  |  |  |  |  |  |  |  |  |  |
| 0 | transcript:Vitvi08g00300\_t001 |  |  |  |  |  |  |  |  |  |  |  |  |
| 0 | transcript:Vitvi08g00301\_t001 |  |  |  |  |  |  |  |  |  |  |  |  |
| 0 | transcript:Vitvi08g04078\_t001 |  |  |  |  |  |  |  |  |  |  |  |  |
| 0 | transcript:Vitvi08g04079\_t001 |  |  |  |  |  |  |  |  |  |  |  |  |
| 0 | transcript:Vitvi08g04080\_t001 |  |  |  |  |  |  |  |  |  |  |  |  |
| 0 | transcript:Vitvi08g04081\_t001 |  |  |  |  |  |  |  |  |  |  |  |  |
| 0 | transcript:Vitvi08g04082\_t001 |  |  |  |  |  |  |  |  |  |  |  |  |
| 0 | transcript:Vitvi08g04083\_t001 |  |  |  |  |  |  |  |  |  |  |  |  |
| 0 | transcript:Vitvi08g00621\_t001 |  |  |  |  |  |  |  |  |  |  |  |  |
| 0 | transcript:Vitvi08g00623\_t001 |  |  |  |  |  |  |  |  |  |  |  |  |
| 0 | transcript:Vitvi08g00624\_t001 |  |  |  |  |  |  |  |  |  |  |  |  |
| 0 | transcript:Vitvi08g04084\_t001 |  |  |  |  |  |  |  |  |  |  |  |  |
| 0 | transcript:Vitvi08g02052\_t001 |  |  |  |  |  |  |  |  |  |  |  |  |
| 0 | transcript:Vitvi08g02051\_t001 |  |  |  |  |  |  |  |  |  |  |  |  |
| 0 | transcript:Vitvi08g04085\_t001 |  |  |  |  |  |  |  |  |  |  |  |  |
| 0 | transcript:Vitvi08g00630\_t001 |  |  |  |  |  |  |  |  |  |  |  |  |
| 0 | transcript:Vitvi08g00632\_t001 |  |  |  |  |  |  |  |  |  |  |  |  |
| 0 | transcript:Vitvi08g00633\_t001 |  |  |  |  |  |  |  |  |  |  |  |  |
| 0 | transcript:Vitvi08g04086\_t001 |  |  |  |  |  |  |  |  |  |  |  |  |
| 0 | transcript:Vitvi08g00636\_t001 |  |  |  |  |  |  |  |  |  |  |  |  |
| 0 | transcript:Vitvi08g00637\_t001 |  |  |  |  |  |  |  |  |  |  |  |  |
| 0 | transcript:Vitvi08g00639\_t001 |  |  |  |  |  |  |  |  |  |  |  |  |
| 0 | transcript:Vitvi08g00640\_t001 |  |  |  |  |  |  |  |  |  |  |  |  |
| 1 | transcript:Vitvi08g00641\_t001 |  | transcript:Vitvi06g00644\_t001 |  |  |  |  |  |  |  |  |  |  |
| 2 | transcript:Vitvi08g00642\_t001 |  | transcript:Vitvi06g00648\_t001 |  | transcript:Vitvi13g00048\_t001 |  |  |  |  |  |  |  |  |
| 2 | transcript:Vitvi08g00643\_t001 |  | \| \| |  | \| \| |  |  |  |  |  |  |  |  |
| 2 | transcript:Vitvi08g00644\_t001 |  | \| \| |  | \| \| |  |  |  |  |  |  |  |  |
| 2 | transcript:Vitvi08g00645\_t001 |  | \| \| |  | transcript:Vitvi13g00051\_t001 |  |  |  |  |  |  |  |  |
| 2 | transcript:Vitvi08g04087\_t001 |  | \| \| |  | \| \| |  |  |  |  |  |  |  |  |
| 2 | transcript:Vitvi08g00650\_t002 |  | \| \| |  | \| \| |  |  |  |  |  |  |  |  |
| 2 | transcript:Vitvi08g00653\_t001 |  | \| \| |  | transcript:Vitvi13g00054\_t001 |  |  |  |  |  |  |  |  |
| 2 | transcript:Vitvi08g00654\_t001 |  | \| \| |  | \| \| |  |  |  |  |  |  |  |  |
| 2 | transcript:Vitvi08g04088\_t001 |  | \| \| |  | \| \| |  |  |  |  |  |  |  |  |
| 2 | transcript:Vitvi08g00656\_t001 |  | \| \| |  | transcript:Vitvi13g00055\_t001 |  |  |  |  |  |  |  |  |
| 2 | transcript:Vitvi08g02055\_t001 |  | \| \| |  | \| \| |  |  |  |  |  |  |  |  |
| 2 | transcript:Vitvi08g00658\_t001 |  | \| \| |  | \| \| |  |  |  |  |  |  |  |  |
| 2 | transcript:Vitvi08g00661\_t001 |  | \| \| |  | \| \| |  |  |  |  |  |  |  |  |
| 2 | transcript:Vitvi08g04089\_t001 |  | \| \| |  | \| \| |  |  |  |  |  |  |  |  |
| 2 | transcript:Vitvi08g02056\_t001 |  | transcript:Vitvi06g01749\_t001 |  | \| \| |  |  |  |  |  |  |  |  |
| 2 | transcript:Vitvi08g00662\_t001 |  | transcript:Vitvi06g00654\_t001 |  | transcript:Vitvi13g00060\_t001 |  |  |  |  |  |  |  |  |
| 2 | transcript:Vitvi08g00665\_t001 |  | \| \| |  | \| \| |  |  |  |  |  |  |  |  |
| 2 | transcript:Vitvi08g00666\_t001 |  | \| \| |  | transcript:Vitvi13g00061\_t001 |  |  |  |  |  |  |  |  |
| 2 | transcript:Vitvi08g04090\_t001 |  | \| \| |  | \| \| |  |  |  |  |  |  |  |  |
| 2 | transcript:Vitvi08g00667\_t001 |  | \| \| |  | \| \| |  |  |  |  |  |  |  |  |
| 2 | transcript:Vitvi08g00668\_t001 |  | \| \| |  | transcript:Vitvi13g00063\_t001 |  |  |  |  |  |  |  |  |
| 2 | transcript:Vitvi08g04091\_t001 |  | transcript:Vitvi06g00658\_t001 |  | \| \| |  |  |  |  |  |  |  |  |
| 2 | transcript:Vitvi08g00671\_t001 |  | \| \| |  | \| \| |  |  |  |  |  |  |  |  |
| 2 | transcript:Vitvi08g00672\_t001 |  | \| \| |  | \| \| |  |  |  |  |  |  |  |  |
| 2 | transcript:Vitvi08g00673\_t001 |  | \| \| |  | transcript:Vitvi13g00066\_t001 |  |  |  |  |  |  |  |  |
| 2 | transcript:Vitvi08g00674\_t002 |  | \| \| |  | \| \| |  |  |  |  |  |  |  |  |
| 2 | transcript:Vitvi08g02058\_t001 |  | \| \| |  | \| \| |  |  |  |  |  |  |  |  |
| 2 | transcript:Vitvi08g00675\_t001 |  | \| \| |  | \| \| |  |  |  |  |  |  |  |  |
| 2 | transcript:Vitvi08g00676\_t002 |  | \| \| |  | \| \| |  |  |  |  |  |  |  |  |
| 2 | transcript:Vitvi08g00678\_t001 |  | \| \| |  | \| \| |  |  |  |  |  |  |  |  |
| 2 | transcript:Vitvi08g00679\_t001 |  | \| \| |  | transcript:Vitvi13g00070\_t001 |  |  |  |  |  |  |  |  |
| 1 | transcript:Vitvi08g04092\_t001 |  | \| \| |  |  |  |  |  |  |  |  |  |  |
| 1 | transcript:Vitvi08g00682\_t001 |  | \| \| |  |  |  |  |  |  |  |  |  |  |
| 1 | transcript:Vitvi08g00684\_t001 |  | \| \| |  |  |  |  |  |  |  |  |  |  |
| 1 | transcript:Vitvi08g00685\_t001 |  | transcript:Vitvi06g00670\_t001 |  |  |  |  |  |  |  |  |  |  |
| 1 | transcript:Vitvi08g00686\_t001 |  | transcript:Vitvi06g00671\_t001 |  |  |  |  |  |  |  |  |  |  |
| 1 | transcript:Vitvi08g00687\_t001 |  | \| \| |  |  |  |  |  |  |  |  |  |  |
| 1 | transcript:Vitvi08g00689\_t001 |  | \| \| |  |  |  |  |  |  |  |  |  |  |
| 1 | transcript:Vitvi08g00690\_t001 |  | transcript:Vitvi06g00673\_t001 |  |  |  |  |  |  |  |  |  |  |
| 0 | transcript:Vitvi08g00691\_t001 |  |  |  |  |  |  |  |  |  |  |  |  |
| 0 | transcript:Vitvi08g04093\_t001 |  |  |  |  |  |  |  |  |  |  |  |  |
| 0 | transcript:Vitvi08g02060\_t001 |  |  |  |  |  |  |  |  |  |  |  |  |
| 0 | transcript:Vitvi08g00700\_t001 |  |  |  |  |  |  |  |  |  |  |  |  |
| 0 | transcript:Vitvi08g04094\_t001 |  |  |  |  |  |  |  |  |  |  |  |  |
| 0 | transcript:Vitvi08g04095\_t001 |  |  |  |  |  |  |  |  |  |  |  |  |
| 0 | transcript:Vitvi08g04096\_t001 |  |  |  |  |  |  |  |  |  |  |  |  |
| 0 | transcript:Vitvi08g00703\_t001 |  |  |  |  |  |  |  |  |  |  |  |  |
| 0 | transcript:Vitvi08g04097\_t001 |  |  |  |  |  |  |  |  |  |  |  |  |
| 0 | transcript:Vitvi08g04098\_t001 |  |  |  |  |  |  |  |  |  |  |  |  |
| 0 | transcript:Vitvi08g04099\_t001 |  |  |  |  |  |  |  |  |  |  |  |  |
| 0 | transcript:Vitvi08g04100\_t001 |  |  |  |  |  |  |  |  |  |  |  |  |
| 0 | transcript:Vitvi08g04101\_t001 |  |  |  |  |  |  |  |  |  |  |  |  |
| 0 | transcript:Vitvi08g02065\_t001 |  |  |  |  |  |  |  |  |  |  |  |  |
| 0 | transcript:Vitvi08g04102\_t001 |  |  |  |  |  |  |  |  |  |  |  |  |
| 0 | transcript:Vitvi08g04103\_t001 |  |  |  |  |  |  |  |  |  |  |  |  |
| 0 | transcript:Vitvi08g04104\_t001 |  |  |  |  |  |  |  |  |  |  |  |  |
| 0 | transcript:Vitvi08g04105\_t001 |  |  |  |  |  |  |  |  |  |  |  |  |
| 0 | transcript:Vitvi08g04106\_t001 |  |  |  |  |  |  |  |  |  |  |  |  |
| 0 | transcript:Vitvi08g02066\_t001 |  |  |  |  |  |  |  |  |  |  |  |  |
| 0 | transcript:Vitvi08g02067\_t001 |  |  |  |  |  |  |  |  |  |  |  |  |
| 0 | transcript:Vitvi08g04107\_t001 |  |  |  |  |  |  |  |  |  |  |  |  |
| 0 | transcript:Vitvi08g02068\_t001 |  |  |  |  |  |  |  |  |  |  |  |  |
| 0 | transcript:Vitvi08g04108\_t001 |  |  |  |  |  |  |  |  |  |  |  |  |
| 0 | transcript:Vitvi08g04109\_t001 |  |  |  |  |  |  |  |  |  |  |  |  |
| 0 | transcript:Vitvi08g02069\_t001 |  |  |  |  |  |  |  |  |  |  |  |  |
| 0 | transcript:Vitvi08g04110\_t001 |  |  |  |  |  |  |  |  |  |  |  |  |
| 0 | transcript:Vitvi08g04111\_t001 |  |  |  |  |  |  |  |  |  |  |  |  |
| 0 | transcript:Vitvi08g04112\_t001 |  |  |  |  |  |  |  |  |  |  |  |  |
| 0 | transcript:Vitvi08g00715\_t001 |  |  |  |  |  |  |  |  |  |  |  |  |
| 0 | transcript:Vitvi08g00716\_t001 |  |  |  |  |  |  |  |  |  |  |  |  |
| 2 | transcript:Vitvi08g00718\_t001 |  | transcript:Vitvi13g00079\_t001 |  | transcript:Vitvi06g01753\_t001 |  |  |  |  |  |  |  |  |
| 2 | transcript:Vitvi08g00719\_t001 |  | \| \| |  | \| \| |  |  |  |  |  |  |  |  |
| 2 | transcript:Vitvi08g00720\_t001 |  | \| \| |  | \| \| |  |  |  |  |  |  |  |  |
| 2 | transcript:Vitvi08g00721\_t001 |  | \| \| |  | transcript:Vitvi06g00680\_t001 |  |  |  |  |  |  |  |  |
| 2 | transcript:Vitvi08g00723\_t001 |  | \| \| |  | \| \| |  |  |  |  |  |  |  |  |
| 2 | transcript:Vitvi08g00725\_t001 |  | \| \| |  | \| \| |  |  |  |  |  |  |  |  |
| 2 | transcript:Vitvi08g00726\_t001 |  | \| \| |  | \| \| |  |  |  |  |  |  |  |  |
| 2 | transcript:Vitvi08g04113\_t001 |  | \| \| |  | \| \| |  |  |  |  |  |  |  |  |
| 2 | transcript:Vitvi08g04114\_t001 |  | \| \| |  | \| \| |  |  |  |  |  |  |  |  |
| 2 | transcript:Vitvi08g00728\_t001 |  | \| \| |  | \| \| |  |  |  |  |  |  |  |  |
| 2 | transcript:Vitvi08g04115\_t001 |  | \| \| |  | \| \| |  |  |  |  |  |  |  |  |
| 2 | transcript:Vitvi08g00729\_t001 |  | \| \| |  | \| \| |  |  |  |  |  |  |  |  |
| 2 | transcript:Vitvi08g00730\_t001 |  | transcript:Vitvi13g00084\_t001 |  | \| \| |  |  |  |  |  |  |  |  |
| 2 | transcript:Vitvi08g00731\_t001 |  | \| \| |  | \| \| |  |  |  |  |  |  |  |  |
| 2 | transcript:Vitvi08g04116\_t001 |  | \| \| |  | \| \| |  |  |  |  |  |  |  |  |
| 2 | transcript:Vitvi08g00735\_t001 |  | \| \| |  | \| \| |  |  |  |  |  |  |  |  |
| 2 | transcript:Vitvi08g04117\_t001 |  | \| \| |  | \| \| |  |  |  |  |  |  |  |  |
| 2 | transcript:Vitvi08g00736\_t001 |  | transcript:Vitvi13g00088\_t001 |  | transcript:Vitvi06g00686\_t001 |  |  |  |  |  |  |  |  |
| 2 | transcript:Vitvi08g00738\_t001 |  | \| \| |  | transcript:Vitvi06g00688\_t001 |  |  |  |  |  |  |  |  |
| 2 | transcript:Vitvi08g02071\_t001 |  | \| \| |  | \| \| |  |  |  |  |  |  |  |  |
| 2 | transcript:Vitvi08g00739\_t001 |  | \| \| |  | \| \| |  |  |  |  |  |  |  |  |
| 2 | transcript:Vitvi08g00740\_t001 |  | transcript:Vitvi13g04016\_t001 |  | \| \| |  |  |  |  |  |  |  |  |
| 2 | transcript:Vitvi08g04118\_t001 |  | \| \| |  | \| \| |  |  |  |  |  |  |  |  |
| 2 | transcript:Vitvi08g04119\_t001 |  | \| \| |  | \| \| |  |  |  |  |  |  |  |  |
| 2 | transcript:Vitvi08g00743\_t001 |  | \| \| |  | \| \| |  |  |  |  |  |  |  |  |
| 2 | transcript:Vitvi08g00744\_t001 |  | \| \| |  | \| \| |  |  |  |  |  |  |  |  |
| 2 | transcript:Vitvi08g00746\_t001 |  | \| \| |  | \| \| |  |  |  |  |  |  |  |  |
| 2 | transcript:Vitvi08g00747\_t001 |  | \| \| |  | \| \| |  |  |  |  |  |  |  |  |
| 2 | transcript:Vitvi08g04120\_t001 |  | \| \| |  | \| \| |  |  |  |  |  |  |  |  |
| 2 | transcript:Vitvi08g00749\_t001 |  | \| \| |  | \| \| |  |  |  |  |  |  |  |  |
| 2 | transcript:Vitvi08g04121\_t001 |  | \| \| |  | \| \| |  |  |  |  |  |  |  |  |
| 2 | transcript:Vitvi08g01969\_t001 |  | \| \| |  | \| \| |  |  |  |  |  |  |  |  |
| 2 | transcript:Vitvi08g00752\_t001 |  | \| \| |  | transcript:Vitvi06g00692\_t003 |  |  |  |  |  |  |  |  |
| 2 | transcript:Vitvi08g02074\_t001 |  | \| \| |  | \| \| |  |  |  |  |  |  |  |  |
| 2 | transcript:Vitvi08g00754\_t001 |  | \| \| |  | transcript:Vitvi06g01759\_t001 |  |  |  |  |  |  |  |  |
| 2 | transcript:Vitvi08g00755\_t002 |  | \| \| |  | \| \| |  |  |  |  |  |  |  |  |
| 2 | transcript:Vitvi08g00756\_t001 |  | \| \| |  | \| \| |  |  |  |  |  |  |  |  |
| 2 | transcript:Vitvi08g00757\_t001 |  | transcript:Vitvi13g00106\_t001 |  | transcript:Vitvi06g00701\_t001.1.6037826e |  |  |  |  |  |  |  |  |
| 2 | transcript:Vitvi08g02076\_t001 |  | transcript:Vitvi13g00107\_t001 |  | transcript:Vitvi06g01761\_t001 |  |  |  |  |  |  |  |  |
| 2 | transcript:Vitvi08g00761\_t001 |  | \| \| |  | \| \| |  |  |  |  |  |  |  |  |
| 2 | transcript:Vitvi08g04122\_t001 |  | \| \| |  | \| \| |  |  |  |  |  |  |  |  |
| 2 | transcript:Vitvi08g04123\_t001 |  | \| \| |  | \| \| |  |  |  |  |  |  |  |  |
| 2 | transcript:Vitvi08g00766\_t001 |  | \| \| |  | transcript:Vitvi06g00720\_t001 |  |  |  |  |  |  |  |  |
| 2 | transcript:Vitvi08g04124\_t001 |  | \| \| |  | \| \| |  |  |  |  |  |  |  |  |
| 2 | transcript:Vitvi08g00767\_t001 |  | \| \| |  | \| \| |  |  |  |  |  |  |  |  |
| 2 | transcript:Vitvi08g00768\_t001 |  | transcript:Vitvi13g00114\_t001 |  | transcript:Vitvi06g00724\_t001 |  |  |  |  |  |  |  |  |
| 2 | transcript:Vitvi08g00769\_t001 |  | \| \| |  | \| \| |  |  |  |  |  |  |  |  |
| 2 | transcript:Vitvi08g00771\_t001 |  | \| \| |  | \| \| |  |  |  |  |  |  |  |  |
| 2 | transcript:Vitvi08g00772\_t001 |  | \| \| |  | \| \| |  |  |  |  |  |  |  |  |
| 2 | transcript:Vitvi08g00773\_t001 |  | \| \| |  | \| \| |  |  |  |  |  |  |  |  |
| 2 | transcript:Vitvi08g00777\_t001 |  | \| \| |  | \| \| |  |  |  |  |  |  |  |  |
| 2 | transcript:Vitvi08g00778\_t001 |  | transcript:Vitvi13g00116\_t001 |  | \| \| |  |  |  |  |  |  |  |  |
| 2 | transcript:Vitvi08g04125\_t001 |  | \| \| |  | \| \| |  |  |  |  |  |  |  |  |
| 2 | transcript:Vitvi08g00780\_t001 |  | \| \| |  | transcript:Vitvi06g04232\_t001 |  |  |  |  |  |  |  |  |
| 2 | transcript:Vitvi08g00781\_t001 |  | transcript:Vitvi13g00120\_t001 |  | \| \| |  |  |  |  |  |  |  |  |
| 2 | transcript:Vitvi08g00782\_t001 |  | transcript:Vitvi13g00121\_t001 |  | \| \| |  |  |  |  |  |  |  |  |
| 2 | transcript:Vitvi08g00783\_t003 |  | \| \| |  | \| \| |  |  |  |  |  |  |  |  |
| 2 | transcript:Vitvi08g00784\_t001 |  | \| \| |  | \| \| |  |  |  |  |  |  |  |  |
| 2 | transcript:Vitvi08g00785\_t001 |  | \| \| |  | \| \| |  |  |  |  |  |  |  |  |
| 2 | transcript:Vitvi08g04126\_t001 |  | \| \| |  | \| \| |  |  |  |  |  |  |  |  |
| 2 | transcript:Vitvi08g00786\_t002 |  | transcript:Vitvi13g00124\_t001 |  | \| \| |  |  |  |  |  |  |  |  |
| 2 | transcript:Vitvi08g00788\_t002 |  | \| \| |  | \| \| |  |  |  |  |  |  |  |  |
| 2 | transcript:Vitvi08g00789\_t001 |  | \| \| |  | \| \| |  |  |  |  |  |  |  |  |
| 2 | transcript:Vitvi08g00790\_t001 |  | \| \| |  | transcript:Vitvi06g00737\_t001 |  |  |  |  |  |  |  |  |
| 2 | transcript:Vitvi08g02080\_t001 |  | \| \| |  | \| \| |  |  |  |  |  |  |  |  |
| 2 | transcript:Vitvi08g00791\_t001 |  | \| \| |  | transcript:Vitvi06g00740\_t001 |  |  |  |  |  |  |  |  |
| 2 | transcript:Vitvi08g00792\_t001 |  | \| \| |  | \| \| |  |  |  |  |  |  |  |  |
| 2 | transcript:Vitvi08g00793\_t001 |  | \| \| |  | transcript:Vitvi06g00741\_t001 |  |  |  |  |  |  |  |  |
| 2 | transcript:Vitvi08g00794\_t001 |  | \| \| |  | transcript:Vitvi06g00742\_t001 |  |  |  |  |  |  |  |  |
| 2 | transcript:Vitvi08g00795\_t002 |  | \| \| |  | \| \| |  |  |  |  |  |  |  |  |
| 2 | transcript:Vitvi08g02084\_t001 |  | \| \| |  | \| \| |  |  |  |  |  |  |  |  |
| 2 | transcript:Vitvi08g04127\_t001 |  | \| \| |  | \| \| |  |  |  |  |  |  |  |  |
| 2 | transcript:Vitvi08g00796\_t001 |  | \| \| |  | \| \| |  |  |  |  |  |  |  |  |
| 2 | transcript:Vitvi08g00797\_t002 |  | transcript:Vitvi13g00129\_t001.1.6037826b |  | \| \| |  |  |  |  |  |  |  |  |
| 2 | transcript:Vitvi08g04128\_t001 |  | \| \| |  | \| \| |  |  |  |  |  |  |  |  |
| 2 | transcript:Vitvi08g00798\_t001 |  | \| \| |  | \| \| |  |  |  |  |  |  |  |  |
| 2 | transcript:Vitvi08g00799\_t001 |  | \| \| |  | \| \| |  |  |  |  |  |  |  |  |
| 2 | transcript:Vitvi08g00801\_t001 |  | \| \| |  | transcript:Vitvi06g00744\_t001 |  |  |  |  |  |  |  |  |
| 2 | transcript:Vitvi08g04129\_t001 |  | \| \| |  | \| \| |  |  |  |  |  |  |  |  |
| 2 | transcript:Vitvi08g00802\_t001 |  | transcript:Vitvi13g00131\_t001 |  | transcript:Vitvi06g00745\_t001 |  |  |  |  |  |  |  |  |
| 2 | transcript:Vitvi08g00803\_t001 |  | transcript:Vitvi13g00132\_t001 |  | \| \| |  |  |  |  |  |  |  |  |
| 2 | transcript:Vitvi08g00804\_t001 |  | \| \| |  | \| \| |  |  |  |  |  |  |  |  |
| 2 | transcript:Vitvi08g00805\_t001 |  | \| \| |  | \| \| |  |  |  |  |  |  |  |  |
| 2 | transcript:Vitvi08g00806\_t001 |  | transcript:Vitvi13g00133\_t001 |  | \| \| |  |  |  |  |  |  |  |  |
| 2 | transcript:Vitvi08g04130\_t001 |  | \| \| |  | \| \| |  |  |  |  |  |  |  |  |
| 2 | transcript:Vitvi08g00808\_t001 |  | \| \| |  | \| \| |  |  |  |  |  |  |  |  |
| 2 | transcript:Vitvi08g00809\_t001 |  | \| \| |  | \| \| |  |  |  |  |  |  |  |  |
| 2 | transcript:Vitvi08g04131\_t001 |  | \| \| |  | \| \| |  |  |  |  |  |  |  |  |
| 2 | transcript:Vitvi08g02086\_t001 |  | \| \| |  | transcript:Vitvi06g01772\_t001 |  |  |  |  |  |  |  |  |
| 2 | transcript:Vitvi08g00812\_t001 |  | \| \| |  | \| \| |  |  |  |  |  |  |  |  |
| 2 | transcript:Vitvi08g02087\_t001 |  | \| \| |  | \| \| |  |  |  |  |  |  |  |  |
| 2 | transcript:Vitvi08g00813\_t001 |  | \| \| |  | \| \| |  |  |  |  |  |  |  |  |
| 2 | transcript:Vitvi08g04132\_t001 |  | \| \| |  | \| \| |  |  |  |  |  |  |  |  |
| 2 | transcript:Vitvi08g00816\_t001 |  | \| \| |  | transcript:Vitvi06g00759\_t001 |  |  |  |  |  |  |  |  |
| 2 | transcript:Vitvi08g00817\_t001 |  | \| \| |  | \| \| |  |  |  |  |  |  |  |  |
| 2 | transcript:Vitvi08g00819\_t001 |  | \| \| |  | \| \| |  |  |  |  |  |  |  |  |
| 2 | transcript:Vitvi08g00820\_t001 |  | transcript:Vitvi13g00139\_t002 |  | transcript:Vitvi06g00763\_t001 |  |  |  |  |  |  |  |  |
| 2 | transcript:Vitvi08g00821\_t001 |  | transcript:Vitvi13g00143\_t001 |  | transcript:Vitvi06g00768\_t001 |  |  |  |  |  |  |  |  |
| 2 | transcript:Vitvi08g00822\_t001 |  | \| \| |  | \| \| |  |  |  |  |  |  |  |  |
| 2 | transcript:Vitvi08g00823\_t001 |  | \| \| |  | transcript:Vitvi06g00769\_t001 |  |  |  |  |  |  |  |  |
| 2 | transcript:Vitvi08g00824\_t001 |  | \| \| |  | \| \| |  |  |  |  |  |  |  |  |
| 2 | transcript:Vitvi08g02089\_t001 |  | transcript:Vitvi13g01903\_t001 |  | transcript:Vitvi06g01776\_t001 |  |  |  |  |  |  |  |  |
| 2 | transcript:Vitvi08g00825\_t002 |  | transcript:Vitvi13g00145\_t001 |  | \| \| |  |  |  |  |  |  |  |  |
| 2 | transcript:Vitvi08g00826\_t002 |  | \| \| |  | \| \| |  |  |  |  |  |  |  |  |
| 2 | transcript:Vitvi08g00827\_t001 |  | \| \| |  | \| \| |  |  |  |  |  |  |  |  |
| 2 | transcript:Vitvi08g00828\_t001 |  | transcript:Vitvi13g00149\_t001 |  | transcript:Vitvi06g00775\_t003 |  |  |  |  |  |  |  |  |
| 2 | transcript:Vitvi08g00829\_t001 |  | \| \| |  | \| \| |  |  |  |  |  |  |  |  |
| 2 | transcript:Vitvi08g04133\_t001 |  | \| \| |  | \| \| |  |  |  |  |  |  |  |  |
| 2 | transcript:Vitvi08g00833\_t001 |  | \| \| |  | \| \| |  |  |  |  |  |  |  |  |
| 2 | transcript:Vitvi08g00834\_t001 |  | transcript:Vitvi13g00150\_t001 |  | \| \| |  |  |  |  |  |  |  |  |
| 2 | transcript:Vitvi08g04134\_t001 |  | \| \| |  | \| \| |  |  |  |  |  |  |  |  |
| 2 | transcript:Vitvi08g00835\_t001 |  | \| \| |  | transcript:Vitvi06g00779\_t001 |  |  |  |  |  |  |  |  |
| 2 | transcript:Vitvi08g00836\_t001 |  | \| \| |  | \| \| |  |  |  |  |  |  |  |  |
| 2 | transcript:Vitvi08g00838\_t001 |  | \| \| |  | \| \| |  |  |  |  |  |  |  |  |
| 2 | transcript:Vitvi08g00839\_t001 |  | \| \| |  | \| \| |  |  |  |  |  |  |  |  |
| 2 | transcript:Vitvi08g00840\_t001 |  | \| \| |  | transcript:Vitvi06g00783\_t001 |  |  |  |  |  |  |  |  |
| 2 | transcript:Vitvi08g00841\_t001 |  | \| \| |  | \| \| |  |  |  |  |  |  |  |  |
| 2 | transcript:Vitvi08g00843\_t001 |  | \| \| |  | transcript:Vitvi06g00784\_t001 |  |  |  |  |  |  |  |  |
| 2 | transcript:Vitvi08g00844\_t001 |  | transcript:Vitvi13g00158\_t001 |  | transcript:Vitvi06g00785\_t001 |  |  |  |  |  |  |  |  |
| 2 | transcript:Vitvi08g00845\_t001 |  | \| \| |  | \| \| |  |  |  |  |  |  |  |  |
| 2 | transcript:Vitvi08g00846\_t001 |  | \| \| |  | \| \| |  |  |  |  |  |  |  |  |
| 2 | transcript:Vitvi08g00847\_t001 |  | \| \| |  | \| \| |  |  |  |  |  |  |  |  |
| 2 | transcript:Vitvi08g00849\_t001 |  | \| \| |  | \| \| |  |  |  |  |  |  |  |  |
| 2 | transcript:Vitvi08g00850\_t001 |  | \| \| |  | \| \| |  |  |  |  |  |  |  |  |
| 2 | transcript:Vitvi08g00851\_t001 |  | \| \| |  | \| \| |  |  |  |  |  |  |  |  |
| 2 | transcript:Vitvi08g00852\_t001 |  | \| \| |  | \| \| |  |  |  |  |  |  |  |  |
| 2 | transcript:Vitvi08g00853\_t001 |  | \| \| |  | transcript:Vitvi06g00799\_t001 |  |  |  |  |  |  |  |  |
| 2 | transcript:Vitvi08g02093\_t001 |  | \| \| |  | \| \| |  |  |  |  |  |  |  |  |
| 2 | transcript:Vitvi08g00854\_t001 |  | transcript:Vitvi13g01913\_t001 |  | \| \| |  |  |  |  |  |  |  |  |
| 2 | transcript:Vitvi08g02094\_t001 |  | \| \| |  | \| \| |  |  |  |  |  |  |  |  |
| 2 | transcript:Vitvi08g04135\_t001 |  | \| \| |  | \| \| |  |  |  |  |  |  |  |  |
| 2 | transcript:Vitvi08g00857\_t001 |  | \| \| |  | transcript:Vitvi06g00801\_t001 |  |  |  |  |  |  |  |  |
| 2 | transcript:Vitvi08g02095\_t001 |  | \| \| |  | \| \| |  |  |  |  |  |  |  |  |
| 2 | transcript:Vitvi08g00858\_t001 |  | \| \| |  | \| \| |  |  |  |  |  |  |  |  |
| 2 | transcript:Vitvi08g00859\_t001 |  | \| \| |  | transcript:Vitvi06g04253\_t001 |  |  |  |  |  |  |  |  |
| 2 | transcript:Vitvi08g00860\_t001 |  | \| \| |  | \| \| |  |  |  |  |  |  |  |  |
| 2 | transcript:Vitvi08g00861\_t001 |  | \| \| |  | \| \| |  |  |  |  |  |  |  |  |
| 2 | transcript:Vitvi08g04136\_t001 |  | \| \| |  | \| \| |  |  |  |  |  |  |  |  |
| 2 | transcript:Vitvi08g00862\_t001 |  | transcript:Vitvi13g00181\_t001 |  | \| \| |  |  |  |  |  |  |  |  |
| 2 | transcript:Vitvi08g00863\_t001 |  | transcript:Vitvi13g00182\_t001 |  | \| \| |  |  |  |  |  |  |  |  |
| 2 | transcript:Vitvi08g00864\_t001 |  | \| \| |  | \| \| |  |  |  |  |  |  |  |  |
| 2 | transcript:Vitvi08g00865\_t001 |  | \| \| |  | \| \| |  |  |  |  |  |  |  |  |
| 2 | transcript:Vitvi08g00866\_t001 |  | transcript:Vitvi13g00187\_t001 |  | \| \| |  |  |  |  |  |  |  |  |
| 2 | transcript:Vitvi08g00867\_t001 |  | \| \| |  | \| \| |  |  |  |  |  |  |  |  |
| 2 | transcript:Vitvi08g00868\_t001 |  | transcript:Vitvi13g01916\_t001 |  | \| \| |  |  |  |  |  |  |  |  |
| 2 | transcript:Vitvi08g00869\_t001 |  | \| \| |  | \| \| |  |  |  |  |  |  |  |  |
| 2 | transcript:Vitvi08g04137\_t001 |  | \| \| |  | \| \| |  |  |  |  |  |  |  |  |
| 2 | transcript:Vitvi08g00870\_t001 |  | transcript:Vitvi13g00191\_t001 |  | \| \| |  |  |  |  |  |  |  |  |
| 1 | transcript:Vitvi08g00871\_t001 |  |  |  | \| \| |  |  |  |  |  |  |  |  |
| 1 | transcript:Vitvi08g00872\_t001 |  |  |  | \| \| |  |  |  |  |  |  |  |  |
| 1 | transcript:Vitvi08g04138\_t001 |  |  |  | \| \| |  |  |  |  |  |  |  |  |
| 1 | transcript:Vitvi08g04139\_t001 |  |  |  | \| \| |  |  |  |  |  |  |  |  |
| 1 | transcript:Vitvi08g00874\_t001 |  |  |  | \| \| |  |  |  |  |  |  |  |  |
| 1 | transcript:Vitvi08g02097\_t001 |  |  |  | \| \| |  |  |  |  |  |  |  |  |
| 1 | transcript:Vitvi08g00877\_t001 |  |  |  | \| \| |  |  |  |  |  |  |  |  |
| 1 | transcript:Vitvi08g04140\_t001 |  |  |  | \| \| |  |  |  |  |  |  |  |  |
| 1 | transcript:Vitvi08g04141\_t001 |  |  |  | \| \| |  |  |  |  |  |  |  |  |
| 1 | transcript:Vitvi08g04142\_t002 |  |  |  | transcript:Vitvi06g00811\_t001 |  |  |  |  |  |  |  |  |
| 1 | transcript:Vitvi08g00881\_t001 |  |  |  | \| \| |  |  |  |  |  |  |  |  |
| 1 | transcript:Vitvi08g00883\_t004 |  |  |  | transcript:Vitvi06g00812\_t001 |  |  |  |  |  |  |  |  |
| 0 | transcript:Vitvi08g00884\_t001 |  |  |  |  |  |  |  |  |  |  |  |  |
| 0 | transcript:Vitvi08g04143\_t001 |  |  |  |  |  |  |  |  |  |  |  |  |
| 0 | transcript:Vitvi08g02099\_t001 |  |  |  |  |  |  |  |  |  |  |  |  |
| 0 | transcript:Vitvi08g02100\_t001 |  |  |  |  |  |  |  |  |  |  |  |  |
| 0 | transcript:Vitvi08g00885\_t001 |  |  |  |  |  |  |  |  |  |  |  |  |
| 0 | transcript:Vitvi08g00886\_t001 |  |  |  |  |  |  |  |  |  |  |  |  |
| 0 | transcript:Vitvi08g00887\_t001 |  |  |  |  |  |  |  |  |  |  |  |  |
| 0 | transcript:Vitvi08g04144\_t001 |  |  |  |  |  |  |  |  |  |  |  |  |
| 0 | transcript:Vitvi08g00888\_t001 |  |  |  |  |  |  |  |  |  |  |  |  |
| 0 | transcript:Vitvi08g00890\_t001 |  |  |  |  |  |  |  |  |  |  |  |  |
| 0 | transcript:Vitvi08g00891\_t001 |  |  |  |  |  |  |  |  |  |  |  |  |
| 0 | transcript:Vitvi08g04145\_t001 |  |  |  |  |  |  |  |  |  |  |  |  |
| 0 | transcript:Vitvi08g02101\_t001 |  |  |  |  |  |  |  |  |  |  |  |  |
| 0 | transcript:Vitvi08g00892\_t002 |  |  |  |  |  |  |  |  |  |  |  |  |
| 0 | transcript:Vitvi08g00893\_t001 |  |  |  |  |  |  |  |  |  |  |  |  |
| 0 | transcript:Vitvi08g04146\_t001 |  |  |  |  |  |  |  |  |  |  |  |  |
| 0 | transcript:Vitvi08g00894\_t001 |  |  |  |  |  |  |  |  |  |  |  |  |
| 0 | transcript:Vitvi08g00895\_t002 |  |  |  |  |  |  |  |  |  |  |  |  |
| 0 | transcript:Vitvi08g02102\_t001 |  |  |  |  |  |  |  |  |  |  |  |  |
| 0 | transcript:Vitvi08g02103\_t001 |  |  |  |  |  |  |  |  |  |  |  |  |
| 0 | transcript:Vitvi08g04147\_t001 |  |  |  |  |  |  |  |  |  |  |  |  |
| 0 | transcript:Vitvi08g00896\_t001 |  |  |  |  |  |  |  |  |  |  |  |  |
| 0 | transcript:Vitvi08g00897\_t001 |  |  |  |  |  |  |  |  |  |  |  |  |
| 0 | transcript:Vitvi08g04148\_t001 |  |  |  |  |  |  |  |  |  |  |  |  |
| 0 | transcript:Vitvi08g04149\_t001 |  |  |  |  |  |  |  |  |  |  |  |  |
| 0 | transcript:Vitvi08g04150\_t001 |  |  |  |  |  |  |  |  |  |  |  |  |
| 0 | transcript:Vitvi08g04151\_t001 |  |  |  |  |  |  |  |  |  |  |  |  |
| 0 | transcript:Vitvi08g00900\_t001 |  |  |  |  |  |  |  |  |  |  |  |  |
| 0 | transcript:Vitvi08g00901\_t001 |  |  |  |  |  |  |  |  |  |  |  |  |
| 0 | transcript:Vitvi08g00902\_t001 |  |  |  |  |  |  |  |  |  |  |  |  |
| 0 | transcript:Vitvi08g00903\_t001 |  |  |  |  |  |  |  |  |  |  |  |  |
| 0 | transcript:Vitvi08g00904\_t001 |  |  |  |  |  |  |  |  |  |  |  |  |
| 0 | transcript:Vitvi08g00905\_t001 |  |  |  |  |  |  |  |  |  |  |  |  |
| 0 | transcript:Vitvi08g02105\_t001 |  |  |  |  |  |  |  |  |  |  |  |  |
| 0 | transcript:Vitvi08g00906\_t001 |  |  |  |  |  |  |  |  |  |  |  |  |
| 0 | transcript:Vitvi08g02106\_t001 |  |  |  |  |  |  |  |  |  |  |  |  |
| 0 | transcript:Vitvi08g02107\_t001 |  |  |  |  |  |  |  |  |  |  |  |  |
| 0 | transcript:Vitvi08g00908\_t003 |  |  |  |  |  |  |  |  |  |  |  |  |
| 0 | transcript:Vitvi08g04152\_t001 |  |  |  |  |  |  |  |  |  |  |  |  |
| 0 | transcript:Vitvi08g00910\_t001 |  |  |  |  |  |  |  |  |  |  |  |  |
| 0 | transcript:Vitvi08g00911\_t001 |  |  |  |  |  |  |  |  |  |  |  |  |
| 0 | transcript:Vitvi08g00912\_t001 |  |  |  |  |  |  |  |  |  |  |  |  |
| 0 | transcript:Vitvi08g00913\_t001 |  |  |  |  |  |  |  |  |  |  |  |  |
| 0 | transcript:Vitvi08g04153\_t001 |  |  |  |  |  |  |  |  |  |  |  |  |
| 0 | transcript:Vitvi08g00914\_t001 |  |  |  |  |  |  |  |  |  |  |  |  |
| 0 | transcript:Vitvi08g00915\_t001 |  |  |  |  |  |  |  |  |  |  |  |  |
| 0 | transcript:Vitvi08g00916\_t001 |  |  |  |  |  |  |  |  |  |  |  |  |
| 0 | transcript:Vitvi08g00917\_t001 |  |  |  |  |  |  |  |  |  |  |  |  |
| 0 | transcript:Vitvi08g00921\_t001 |  |  |  |  |  |  |  |  |  |  |  |  |
| 0 | transcript:Vitvi08g00922\_t001 |  |  |  |  |  |  |  |  |  |  |  |  |
| 0 | transcript:Vitvi08g04154\_t001 |  |  |  |  |  |  |  |  |  |  |  |  |
| 0 | transcript:Vitvi08g00923\_t001 |  |  |  |  |  |  |  |  |  |  |  |  |
| 0 | transcript:Vitvi08g00924\_t001 |  |  |  |  |  |  |  |  |  |  |  |  |
| 0 | transcript:Vitvi08g00925\_t001 |  |  |  |  |  |  |  |  |  |  |  |  |
| 0 | transcript:Vitvi08g00926\_t001 |  |  |  |  |  |  |  |  |  |  |  |  |
| 0 | transcript:Vitvi08g02109\_t001 |  |  |  |  |  |  |  |  |  |  |  |  |
| 0 | transcript:Vitvi08g00928\_t001 |  |  |  |  |  |  |  |  |  |  |  |  |
| 0 | transcript:Vitvi08g02111\_t001 |  |  |  |  |  |  |  |  |  |  |  |  |
| 0 | transcript:Vitvi08g00929\_t001 |  |  |  |  |  |  |  |  |  |  |  |  |
| 0 | transcript:Vitvi08g00930\_t003 |  |  |  |  |  |  |  |  |  |  |  |  |
| 0 | transcript:Vitvi08g04155\_t001 |  |  |  |  |  |  |  |  |  |  |  |  |
| 0 | transcript:Vitvi08g00931\_t001 |  |  |  |  |  |  |  |  |  |  |  |  |
| 0 | transcript:Vitvi08g02112\_t001 |  |  |  |  |  |  |  |  |  |  |  |  |
| 0 | transcript:Vitvi08g00932\_t001 |  |  |  |  |  |  |  |  |  |  |  |  |
| 0 | transcript:Vitvi08g00933\_t001 |  |  |  |  |  |  |  |  |  |  |  |  |
| 0 | transcript:Vitvi08g00934\_t001 |  |  |  |  |  |  |  |  |  |  |  |  |
| 0 | transcript:Vitvi08g00935\_t001 |  |  |  |  |  |  |  |  |  |  |  |  |
| 0 | transcript:Vitvi08g00936\_t001 |  |  |  |  |  |  |  |  |  |  |  |  |
| 0 | transcript:Vitvi08g04156\_t001 |  |  |  |  |  |  |  |  |  |  |  |  |
| 0 | transcript:Vitvi08g04157\_t001 |  |  |  |  |  |  |  |  |  |  |  |  |
| 0 | transcript:Vitvi08g02113\_t001 |  |  |  |  |  |  |  |  |  |  |  |  |
| 0 | transcript:Vitvi08g00937\_t001 |  |  |  |  |  |  |  |  |  |  |  |  |
| 0 | transcript:Vitvi08g00939\_t001 |  |  |  |  |  |  |  |  |  |  |  |  |
| 0 | transcript:Vitvi08g00940\_t001 |  |  |  |  |  |  |  |  |  |  |  |  |
| 0 | transcript:Vitvi08g00941\_t001 |  |  |  |  |  |  |  |  |  |  |  |  |
| 0 | transcript:Vitvi08g00942\_t001 |  |  |  |  |  |  |  |  |  |  |  |  |
| 0 | transcript:Vitvi08g04158\_t001 |  |  |  |  |  |  |  |  |  |  |  |  |
| 0 | transcript:Vitvi08g00943\_t001 |  |  |  |  |  |  |  |  |  |  |  |  |
| 0 | transcript:Vitvi08g00944\_t001 |  |  |  |  |  |  |  |  |  |  |  |  |
| 0 | transcript:Vitvi08g00945\_t001 |  |  |  |  |  |  |  |  |  |  |  |  |
| 0 | transcript:Vitvi08g00946\_t001 |  |  |  |  |  |  |  |  |  |  |  |  |
| 0 | transcript:Vitvi08g00947\_t001 |  |  |  |  |  |  |  |  |  |  |  |  |
| 0 | transcript:Vitvi08g04159\_t001 |  |  |  |  |  |  |  |  |  |  |  |  |
| 0 | transcript:Vitvi08g00948\_t001 |  |  |  |  |  |  |  |  |  |  |  |  |
| 0 | transcript:Vitvi08g02114\_t001 |  |  |  |  |  |  |  |  |  |  |  |  |
| 0 | transcript:Vitvi08g04160\_t001 |  |  |  |  |  |  |  |  |  |  |  |  |
| 0 | transcript:Vitvi08g00949\_t001 |  |  |  |  |  |  |  |  |  |  |  |  |
| 0 | transcript:Vitvi08g04161\_t001 |  |  |  |  |  |  |  |  |  |  |  |  |
| 0 | transcript:Vitvi08g00950\_t001 |  |  |  |  |  |  |  |  |  |  |  |  |
| 0 | transcript:Vitvi08g04162\_t001 |  |  |  |  |  |  |  |  |  |  |  |  |
| 0 | transcript:Vitvi08g00953\_t001 |  |  |  |  |  |  |  |  |  |  |  |  |
| 0 | transcript:Vitvi08g00954\_t003 |  |  |  |  |  |  |  |  |  |  |  |  |
| 0 | transcript:Vitvi08g00955\_t001 |  |  |  |  |  |  |  |  |  |  |  |  |
| 0 | transcript:Vitvi08g00956\_t001 |  |  |  |  |  |  |  |  |  |  |  |  |
| 0 | transcript:Vitvi08g00957\_t001 |  |  |  |  |  |  |  |  |  |  |  |  |
| 0 | transcript:Vitvi08g00958\_t001 |  |  |  |  |  |  |  |  |  |  |  |  |
| 0 | transcript:Vitvi08g04163\_t001 |  |  |  |  |  |  |  |  |  |  |  |  |
| 0 | transcript:Vitvi08g00959\_t001 |  |  |  |  |  |  |  |  |  |  |  |  |
| 0 | transcript:Vitvi08g00960\_t001 |  |  |  |  |  |  |  |  |  |  |  |  |
| 0 | transcript:Vitvi08g00961\_t001 |  |  |  |  |  |  |  |  |  |  |  |  |
| 0 | transcript:Vitvi08g00962\_t001 |  |  |  |  |  |  |  |  |  |  |  |  |
| 0 | transcript:Vitvi08g00963\_t001 |  |  |  |  |  |  |  |  |  |  |  |  |
| 0 | transcript:Vitvi08g00965\_t001 |  |  |  |  |  |  |  |  |  |  |  |  |
| 0 | transcript:Vitvi08g00967\_t001 |  |  |  |  |  |  |  |  |  |  |  |  |
| 0 | transcript:Vitvi08g04164\_t001 |  |  |  |  |  |  |  |  |  |  |  |  |
| 0 | transcript:Vitvi08g02126\_t001 |  |  |  |  |  |  |  |  |  |  |  |  |
| 0 | transcript:Vitvi08g04165\_t003 |  |  |  |  |  |  |  |  |  |  |  |  |
| 0 | transcript:Vitvi08g02128\_t001 |  |  |  |  |  |  |  |  |  |  |  |  |
| 0 | transcript:Vitvi08g02129\_t001 |  |  |  |  |  |  |  |  |  |  |  |  |
| 0 | transcript:Vitvi08g04166\_t001 |  |  |  |  |  |  |  |  |  |  |  |  |
| 0 | transcript:Vitvi08g00970\_t001 |  |  |  |  |  |  |  |  |  |  |  |  |
| 0 | transcript:Vitvi08g00971\_t001 |  |  |  |  |  |  |  |  |  |  |  |  |
| 0 | transcript:Vitvi08g04167\_t001 |  |  |  |  |  |  |  |  |  |  |  |  |
| 0 | transcript:Vitvi08g00972\_t001 |  |  |  |  |  |  |  |  |  |  |  |  |
| 0 | transcript:Vitvi08g04168\_t001 |  |  |  |  |  |  |  |  |  |  |  |  |
| 0 | transcript:Vitvi08g02131\_t002 |  |  |  |  |  |  |  |  |  |  |  |  |
| 0 | transcript:Vitvi08g00974\_t001 |  |  |  |  |  |  |  |  |  |  |  |  |
| 0 | transcript:Vitvi08g00976\_t001 |  |  |  |  |  |  |  |  |  |  |  |  |
| 0 | transcript:Vitvi08g00977\_t001 |  |  |  |  |  |  |  |  |  |  |  |  |
| 0 | transcript:Vitvi08g00978\_t001 |  |  |  |  |  |  |  |  |  |  |  |  |
| 0 | transcript:Vitvi08g04169\_t001 |  |  |  |  |  |  |  |  |  |  |  |  |
| 0 | transcript:Vitvi08g00979\_t001 |  |  |  |  |  |  |  |  |  |  |  |  |
| 0 | transcript:Vitvi08g00981\_t001 |  |  |  |  |  |  |  |  |  |  |  |  |
| 0 | transcript:Vitvi08g00982\_t001 |  |  |  |  |  |  |  |  |  |  |  |  |
| 0 | transcript:Vitvi08g00983\_t001 |  |  |  |  |  |  |  |  |  |  |  |  |
| 0 | transcript:Vitvi08g00984\_t001 |  |  |  |  |  |  |  |  |  |  |  |  |
| 0 | transcript:Vitvi08g00986\_t001 |  |  |  |  |  |  |  |  |  |  |  |  |
| 0 | transcript:Vitvi08g00987\_t001 |  |  |  |  |  |  |  |  |  |  |  |  |
| 0 | transcript:Vitvi08g00988\_t001 |  |  |  |  |  |  |  |  |  |  |  |  |
| 0 | transcript:Vitvi08g04170\_t001 |  |  |  |  |  |  |  |  |  |  |  |  |
| 0 | transcript:Vitvi08g04171\_t001 |  |  |  |  |  |  |  |  |  |  |  |  |
| 0 | transcript:Vitvi08g00989\_t001 |  |  |  |  |  |  |  |  |  |  |  |  |
| 1 | transcript:Vitvi08g00992\_t001 |  | transcript:Vitvi06g01315\_t001 |  |  |  |  |  |  |  |  |  |  |
| 1 | transcript:Vitvi08g00993\_t001 |  | \| \| |  |  |  |  |  |  |  |  |  |  |
| 1 | transcript:Vitvi08g02133\_t001 |  | transcript:Vitvi06g01310\_t001 |  |  |  |  |  |  |  |  |  |  |
| 1 | transcript:Vitvi08g00994\_t001 |  | \| \| |  |  |  |  |  |  |  |  |  |  |
| 1 | transcript:Vitvi08g00995\_t001 |  | \| \| |  |  |  |  |  |  |  |  |  |  |
| 1 | transcript:Vitvi08g00996\_t001 |  | \| \| |  |  |  |  |  |  |  |  |  |  |
| 1 | transcript:Vitvi08g00998\_t001 |  | \| \| |  |  |  |  |  |  |  |  |  |  |
| 1 | transcript:Vitvi08g02134\_t001 |  | \| \| |  |  |  |  |  |  |  |  |  |  |
| 1 | transcript:Vitvi08g02135\_t001 |  | \| \| |  |  |  |  |  |  |  |  |  |  |
| 1 | transcript:Vitvi08g04172\_t001 |  | \| \| |  |  |  |  |  |  |  |  |  |  |
| 1 | transcript:Vitvi08g00999\_t001 |  | \| \| |  |  |  |  |  |  |  |  |  |  |
| 1 | transcript:Vitvi08g04173\_t001 |  | \| \| |  |  |  |  |  |  |  |  |  |  |
| 1 | transcript:Vitvi08g02136\_t001 |  | \| \| |  |  |  |  |  |  |  |  |  |  |
| 1 | transcript:Vitvi08g01001\_t001 |  | transcript:Vitvi06g01307\_t001 |  |  |  |  |  |  |  |  |  |  |
| 1 | transcript:Vitvi08g01002\_t001 |  | transcript:Vitvi06g01303\_t001 |  |  |  |  |  |  |  |  |  |  |
| 1 | transcript:Vitvi08g02137\_t001 |  | \| \| |  |  |  |  |  |  |  |  |  |  |
| 1 | transcript:Vitvi08g01004\_t001 |  | transcript:Vitvi06g01299\_t001 |  |  |  |  |  |  |  |  |  |  |
| 1 | transcript:Vitvi08g04174\_t001 |  | \| \| |  |  |  |  |  |  |  |  |  |  |
| 1 | transcript:Vitvi08g01006\_t001 |  | \| \| |  |  |  |  |  |  |  |  |  |  |
| 1 | transcript:Vitvi08g01007\_t001 |  | \| \| |  |  |  |  |  |  |  |  |  |  |
| 1 | transcript:Vitvi08g02139\_t001 |  | \| \| |  |  |  |  |  |  |  |  |  |  |
| 1 | transcript:Vitvi08g01008\_t001 |  | \| \| |  |  |  |  |  |  |  |  |  |  |
| 1 | transcript:Vitvi08g01009\_t001 |  | \| \| |  |  |  |  |  |  |  |  |  |  |
| 2 | transcript:Vitvi08g01010\_t005 |  | \| \| |  | transcript:Vitvi13g00640\_t001 |  |  |  |  |  |  |  |  |
| 2 | transcript:Vitvi08g04175\_t001 |  | \| \| |  | \| \| |  |  |  |  |  |  |  |  |
| 3 | transcript:Vitvi08g01011\_t001 |  | \| \| |  | \| \| |  | transcript:Vitvi06g01646\_t001 |  |  |  |  |  |  |
| 3 | transcript:Vitvi08g01012\_t002 |  | transcript:Vitvi06g01297\_t001 |  | \| \| |  | \| \| |  |  |  |  |  |  |
| 3 | transcript:Vitvi08g01013\_t001 |  | transcript:Vitvi06g01289\_t001 |  | transcript:Vitvi13g00638\_t001 |  | transcript:Vitvi06g00242\_t001 |  |  |  |  |  |  |
| 3 | transcript:Vitvi08g01014\_t001 |  | \| \| |  | \| \| |  | \| \| |  |  |  |  |  |  |
| 3 | transcript:Vitvi08g02140\_t003 |  | \| \| |  | \| \| |  | transcript:Vitvi06g00245\_t001 |  |  |  |  |  |  |
| 3 | transcript:Vitvi08g01015\_t001 |  | \| \| |  | transcript:Vitvi13g00632\_t001 |  | transcript:Vitvi06g00246\_t001 |  |  |  |  |  |  |
| 3 | transcript:Vitvi08g01016\_t001 |  | \| \| |  | \| \| |  | \| \| |  |  |  |  |  |  |
| 3 | transcript:Vitvi08g01018\_t001 |  | \| \| |  | \| \| |  | \| \| |  |  |  |  |  |  |
| 3 | transcript:Vitvi08g01019\_t001 |  | \| \| |  | \| \| |  | \| \| |  |  |  |  |  |  |
| 3 | transcript:Vitvi08g04176\_t001 |  | \| \| |  | \| \| |  | \| \| |  |  |  |  |  |  |
| 3 | transcript:Vitvi08g01021\_t001 |  | \| \| |  | \| \| |  | \| \| |  |  |  |  |  |  |
| 3 | transcript:Vitvi08g04177\_t001 |  | \| \| |  | \| \| |  | \| \| |  |  |  |  |  |  |
| 3 | transcript:Vitvi08g01023\_t001 |  | transcript:Vitvi06g01273\_t001 |  | \| \| |  | \| \| |  |  |  |  |  |  |
| 2 | transcript:Vitvi08g01024\_t001 |  |  |  | transcript:Vitvi13g00621\_t001 |  | \| \| |  |  |  |  |  |  |
| 2 | transcript:Vitvi08g01026\_t001 |  |  |  | \| \| |  | \| \| |  |  |  |  |  |  |
| 2 | transcript:Vitvi08g01027\_t001 |  |  |  | \| \| |  | \| \| |  |  |  |  |  |  |
| 2 | transcript:Vitvi08g01028\_t001 |  |  |  | \| \| |  | transcript:Vitvi06g00262\_t001 |  |  |  |  |  |  |
| 2 | transcript:Vitvi08g01029\_t001 |  |  |  | \| \| |  | \| \| |  |  |  |  |  |  |
| 2 | transcript:Vitvi08g01030\_t001 |  |  |  | \| \| |  | transcript:Vitvi06g00263\_t001 |  |  |  |  |  |  |
| 2 | transcript:Vitvi08g01031\_t001 |  |  |  | \| \| |  | \| \| |  |  |  |  |  |  |
| 2 | transcript:Vitvi08g01032\_t001 |  |  |  | transcript:Vitvi13g00618\_t001 |  | \| \| |  |  |  |  |  |  |
| 2 | transcript:Vitvi08g01033\_t001 |  |  |  | transcript:Vitvi13g02058\_t001 |  | transcript:Vitvi06g00272\_t002 |  |  |  |  |  |  |
| 2 | transcript:Vitvi08g02143\_t001 |  |  |  | \| \| |  | \| \| |  |  |  |  |  |  |
| 2 | transcript:Vitvi08g01035\_t001 |  |  |  | transcript:Vitvi13g00610\_t001 |  | transcript:Vitvi06g00275\_t001 |  |  |  |  |  |  |
| 2 | transcript:Vitvi08g02145\_t001 |  |  |  | \| \| |  | \| \| |  |  |  |  |  |  |
| 2 | transcript:Vitvi08g01036\_t001 |  |  |  | \| \| |  | transcript:Vitvi06g00279\_t001 |  |  |  |  |  |  |
| 2 | transcript:Vitvi08g04178\_t001 |  |  |  | \| \| |  | \| \| |  |  |  |  |  |  |
| 2 | transcript:Vitvi08g01037\_t001 |  |  |  | transcript:Vitvi13g00606\_t001 |  | transcript:Vitvi06g00280\_t001 |  |  |  |  |  |  |
| 2 | transcript:Vitvi08g01038\_t001 |  |  |  | transcript:Vitvi13g00605\_t001 |  | transcript:Vitvi06g00281\_t001 |  |  |  |  |  |  |
| 2 | transcript:Vitvi08g01039\_t002 |  |  |  | transcript:Vitvi13g00601\_t001 |  | \| \| |  |  |  |  |  |  |
| 2 | transcript:Vitvi08g04179\_t001 |  |  |  | \| \| |  | \| \| |  |  |  |  |  |  |
| 2 | transcript:Vitvi08g01040\_t001 |  |  |  | \| \| |  | \| \| |  |  |  |  |  |  |
| 2 | transcript:Vitvi08g01041\_t001 |  |  |  | transcript:Vitvi13g00592\_t001 |  | \| \| |  |  |  |  |  |  |
| 1 | transcript:Vitvi08g01042\_t001 |  |  |  |  |  | transcript:Vitvi06g00294\_t001 |  |  |  |  |  |  |
| 1 | transcript:Vitvi08g01043\_t001 |  |  |  |  |  | \| \| |  |  |  |  |  |  |
| 1 | transcript:Vitvi08g01044\_t001 |  |  |  |  |  | transcript:Vitvi06g00302\_t001 |  |  |  |  |  |  |
| 1 | transcript:Vitvi08g02147\_t001 |  |  |  |  |  | \| \| |  |  |  |  |  |  |
| 1 | transcript:Vitvi08g02148\_t001 |  |  |  |  |  | \| \| |  |  |  |  |  |  |
| 1 | transcript:Vitvi08g02149\_t001 |  |  |  |  |  | \| \| |  |  |  |  |  |  |
| 1 | transcript:Vitvi08g02150\_t001 |  |  |  |  |  | \| \| |  |  |  |  |  |  |
| 1 | transcript:Vitvi08g02151\_t001 |  |  |  |  |  | \| \| |  |  |  |  |  |  |
| 1 | transcript:Vitvi08g04180\_t001 |  |  |  |  |  | \| \| |  |  |  |  |  |  |
| 1 | transcript:Vitvi08g02152\_t001 |  |  |  |  |  | \| \| |  |  |  |  |  |  |
| 1 | transcript:Vitvi08g02153\_t001 |  |  |  |  |  | \| \| |  |  |  |  |  |  |
| 1 | transcript:Vitvi08g02154\_t001 |  |  |  |  |  | \| \| |  |  |  |  |  |  |
| 1 | transcript:Vitvi08g02155\_t001 |  |  |  |  |  | \| \| |  |  |  |  |  |  |
| 1 | transcript:Vitvi08g04181\_t001 |  |  |  |  |  | \| \| |  |  |  |  |  |  |
| 1 | transcript:Vitvi08g02156\_t001 |  |  |  |  |  | transcript:Vitvi06g00303\_t001 |  |  |  |  |  |  |
| 1 | transcript:Vitvi08g02157\_t001 |  |  |  |  |  | \| \| |  |  |  |  |  |  |
| 1 | transcript:Vitvi08g04182\_t001 |  |  |  |  |  | \| \| |  |  |  |  |  |  |
| 1 | transcript:Vitvi08g02158\_t001 |  |  |  |  |  | \| \| |  |  |  |  |  |  |
| 1 | transcript:Vitvi08g02159\_t001 |  |  |  |  |  | \| \| |  |  |  |  |  |  |
| 1 | transcript:Vitvi08g02160\_t001 |  |  |  |  |  | \| \| |  |  |  |  |  |  |
| 1 | transcript:Vitvi08g04183\_t001 |  |  |  |  |  | \| \| |  |  |  |  |  |  |
| 1 | transcript:Vitvi08g01047\_t001 |  |  |  |  |  | \| \| |  |  |  |  |  |  |
| 1 | transcript:Vitvi08g01049\_t001 |  |  |  |  |  | \| \| |  |  |  |  |  |  |
| 1 | transcript:Vitvi08g01050\_t003 |  |  |  |  |  | transcript:Vitvi06g00304\_t001 |  |  |  |  |  |  |
| 1 | transcript:Vitvi08g01052\_t001 |  |  |  |  |  | transcript:Vitvi06g00305\_t001 |  |  |  |  |  |  |
| 1 | transcript:Vitvi08g01053\_t001 |  |  |  |  |  | transcript:Vitvi06g00306\_t001 |  |  |  |  |  |  |
| 1 | transcript:Vitvi08g04184\_t001 |  |  |  |  |  | \| \| |  |  |  |  |  |  |
| 2 | transcript:Vitvi08g01054\_t001 |  | transcript:Vitvi13g00585\_t001 |  |  |  | \| \| |  |  |  |  |  |  |
| 2 | transcript:Vitvi08g01055\_t001 |  | transcript:Vitvi13g00578\_t001 |  |  |  | transcript:Vitvi06g00308\_t001 |  |  |  |  |  |  |
| 2 | transcript:Vitvi08g01056\_t001 |  | \| \| |  |  |  | \| \| |  |  |  |  |  |  |
| 2 | transcript:Vitvi08g01057\_t002 |  | \| \| |  |  |  | \| \| |  |  |  |  |  |  |
| 2 | transcript:Vitvi08g01058\_t001 |  | \| \| |  |  |  | \| \| |  |  |  |  |  |  |
| 2 | transcript:Vitvi08g04185\_t001 |  | \| \| |  |  |  | \| \| |  |  |  |  |  |  |
| 2 | transcript:Vitvi08g01059\_t001 |  | \| \| |  |  |  | \| \| |  |  |  |  |  |  |
| 2 | transcript:Vitvi08g02161\_t003 |  | \| \| |  |  |  | transcript:Vitvi06g01662\_t001 |  |  |  |  |  |  |
| 2 | transcript:Vitvi08g01060\_t002 |  | \| \| |  |  |  | \| \| |  |  |  |  |  |  |
| 2 | transcript:Vitvi08g02162\_t001 |  | \| \| |  |  |  | \| \| |  |  |  |  |  |  |
| 2 | transcript:Vitvi08g01061\_t001 |  | \| \| |  |  |  | \| \| |  |  |  |  |  |  |
| 2 | transcript:Vitvi08g01062\_t001 |  | \| \| |  |  |  | \| \| |  |  |  |  |  |  |
| 2 | transcript:Vitvi08g01063\_t001 |  | \| \| |  |  |  | \| \| |  |  |  |  |  |  |
| 2 | transcript:Vitvi08g01065\_t001 |  | \| \| |  |  |  | \| \| |  |  |  |  |  |  |
| 2 | transcript:Vitvi08g01067\_t001 |  | \| \| |  |  |  | \| \| |  |  |  |  |  |  |
| 2 | transcript:Vitvi08g02163\_t001 |  | \| \| |  |  |  | \| \| |  |  |  |  |  |  |
| 2 | transcript:Vitvi08g01068\_t001 |  | \| \| |  |  |  | \| \| |  |  |  |  |  |  |
| 2 | transcript:Vitvi08g01069\_t001 |  | transcript:Vitvi13g02054\_t001 |  |  |  | transcript:Vitvi06g04107\_t001 |  |  |  |  |  |  |
| 2 | transcript:Vitvi08g01070\_t001 |  | \| \| |  |  |  | \| \| |  |  |  |  |  |  |
| 2 | transcript:Vitvi08g01071\_t001 |  | transcript:Vitvi13g00563\_t002 |  |  |  | \| \| |  |  |  |  |  |  |
| 2 | transcript:Vitvi08g01072\_t001 |  | transcript:Vitvi13g02052\_t001 |  |  |  | \| \| |  |  |  |  |  |  |
| 2 | transcript:Vitvi08g01073\_t001 |  | \| \| |  |  |  | transcript:Vitvi06g00331\_t001 |  |  |  |  |  |  |
| 2 | transcript:Vitvi08g04186\_t001 |  | \| \| |  |  |  | \| \| |  |  |  |  |  |  |
| 2 | transcript:Vitvi08g01075\_t003 |  | \| \| |  |  |  | transcript:Vitvi06g01665\_t004 |  |  |  |  |  |  |
| 2 | transcript:Vitvi08g02164\_t001 |  | \| \| |  |  |  | \| \| |  |  |  |  |  |  |
| 2 | transcript:Vitvi08g01076\_t001 |  | transcript:Vitvi13g04156\_t001 |  |  |  | transcript:Vitvi06g04112\_t001 |  |  |  |  |  |  |
| 1 | transcript:Vitvi08g01077\_t001 |  | transcript:Vitvi13g00538\_t001 |  |  |  |  |  |  |  |  |  |  |
| 1 | transcript:Vitvi08g01078\_t001 |  | transcript:Vitvi13g00537\_t004 |  |  |  |  |  |  |  |  |  |  |
| 1 | transcript:Vitvi08g01079\_t001 |  | transcript:Vitvi06g00421\_t001 |  |  |  |  |  |  |  |  |  |  |
| 2 | transcript:Vitvi08g04187\_t001 |  | \| \| |  | transcript:Vitvi13g04080\_t001 |  |  |  |  |  |  |  |  |
| 2 | transcript:Vitvi08g01081\_t001 |  | \| \| |  | \| \| |  |  |  |  |  |  |  |  |
| 2 | transcript:Vitvi08g01082\_t004 |  | transcript:Vitvi06g00420\_t001 |  | transcript:Vitvi13g00266\_t001 |  |  |  |  |  |  |  |  |
| 2 | transcript:Vitvi08g01083\_t001 |  | \| \| |  | transcript:Vitvi13g00264\_t001 |  |  |  |  |  |  |  |  |
| 2 | transcript:Vitvi08g04188\_t001 |  | transcript:Vitvi06g00419\_t001 |  | transcript:Vitvi13g00263\_t001 |  |  |  |  |  |  |  |  |
| 2 | transcript:Vitvi08g04189\_t001 |  | \| \| |  | \| \| |  |  |  |  |  |  |  |  |
| 2 | transcript:Vitvi08g01085\_t001 |  | \| \| |  | \| \| |  |  |  |  |  |  |  |  |
| 2 | transcript:Vitvi08g01087\_t001 |  | \| \| |  | \| \| |  |  |  |  |  |  |  |  |
| 2 | transcript:Vitvi08g04190\_t001 |  | \| \| |  | \| \| |  |  |  |  |  |  |  |  |
| 2 | transcript:Vitvi08g02166\_t001 |  | \| \| |  | transcript:Vitvi13g00258\_t001 |  |  |  |  |  |  |  |  |
| 2 | transcript:Vitvi08g02167\_t001 |  | \| \| |  | transcript:Vitvi13g01953\_t001 |  |  |  |  |  |  |  |  |
| 2 | transcript:Vitvi08g02168\_t002 |  | transcript:Vitvi06g00411\_t001 |  | \| \| |  |  |  |  |  |  |  |  |
| 2 | transcript:Vitvi08g01089\_t001 |  | \| \| |  | \| \| |  |  |  |  |  |  |  |  |
| 2 | transcript:Vitvi08g04191\_t001 |  | \| \| |  | \| \| |  |  |  |  |  |  |  |  |
| 2 | transcript:Vitvi08g01090\_t001 |  | transcript:Vitvi06g00408\_t001 |  | transcript:Vitvi13g00249\_t001 |  |  |  |  |  |  |  |  |
| 1 | transcript:Vitvi08g01091\_t002 |  | \| \| |  |  |  |  |  |  |  |  |  |  |
| 2 | transcript:Vitvi08g02169\_t001 |  | \| \| |  | transcript:Vitvi13g01932\_t001 |  |  |  |  |  |  |  |  |
| 2 | transcript:Vitvi08g02170\_t001 |  | \| \| |  | \| \| |  |  |  |  |  |  |  |  |
| 2 | transcript:Vitvi08g01095\_t001 |  | \| \| |  | \| \| |  |  |  |  |  |  |  |  |
| 2 | transcript:Vitvi08g01096\_t001 |  | \| \| |  | transcript:Vitvi13g00240\_t001 |  |  |  |  |  |  |  |  |
| 2 | transcript:Vitvi08g02171\_t001 |  | \| \| |  | \| \| |  |  |  |  |  |  |  |  |
| 2 | transcript:Vitvi08g02172\_t001 |  | \| \| |  | \| \| |  |  |  |  |  |  |  |  |
| 2 | transcript:Vitvi08g04192\_t001 |  | \| \| |  | \| \| |  |  |  |  |  |  |  |  |
| 2 | transcript:Vitvi08g01097\_t001 |  | transcript:Vitvi06g00399\_t001 |  | \| \| |  |  |  |  |  |  |  |  |
| 2 | transcript:Vitvi08g01098\_t001 |  | \| \| |  | \| \| |  |  |  |  |  |  |  |  |
| 2 | transcript:Vitvi08g01099\_t001 |  | \| \| |  | \| \| |  |  |  |  |  |  |  |  |
| 2 | transcript:Vitvi08g04193\_t001 |  | \| \| |  | \| \| |  |  |  |  |  |  |  |  |
| 2 | transcript:Vitvi08g04194\_t001 |  | \| \| |  | \| \| |  |  |  |  |  |  |  |  |
| 2 | transcript:Vitvi08g04195\_t001 |  | \| \| |  | \| \| |  |  |  |  |  |  |  |  |
| 2 | transcript:Vitvi08g01101\_t001 |  | transcript:Vitvi06g00397\_t001 |  | transcript:Vitvi13g00233\_t001 |  |  |  |  |  |  |  |  |
| 2 | transcript:Vitvi08g01102\_t001 |  | \| \| |  | transcript:Vitvi13g00232\_t001 |  |  |  |  |  |  |  |  |
| 2 | transcript:Vitvi08g01103\_t001 |  | transcript:Vitvi06g00393\_t001 |  | transcript:Vitvi13g00229\_t001 |  |  |  |  |  |  |  |  |
| 2 | transcript:Vitvi08g01104\_t003 |  | \| \| |  | \| \| |  |  |  |  |  |  |  |  |
| 2 | transcript:Vitvi08g01105\_t001 |  | \| \| |  | transcript:Vitvi13g01929\_t001 |  |  |  |  |  |  |  |  |
| 2 | transcript:Vitvi08g01107\_t001 |  | \| \| |  | transcript:Vitvi13g00227\_t002 |  |  |  |  |  |  |  |  |
| 2 | transcript:Vitvi08g04196\_t001 |  | \| \| |  | \| \| |  |  |  |  |  |  |  |  |
| 2 | transcript:Vitvi08g01108\_t001 |  | \| \| |  | \| \| |  |  |  |  |  |  |  |  |
| 2 | transcript:Vitvi08g04197\_t001 |  | \| \| |  | \| \| |  |  |  |  |  |  |  |  |
| 2 | transcript:Vitvi08g01109\_t001 |  | \| \| |  | transcript:Vitvi13g00226\_t001 |  |  |  |  |  |  |  |  |
| 2 | transcript:Vitvi08g04198\_t001 |  | transcript:Vitvi06g00387\_t001 |  | \| \| |  |  |  |  |  |  |  |  |
| 2 | transcript:Vitvi08g01111\_t001 |  | transcript:Vitvi06g00386\_t002 |  | \| \| |  |  |  |  |  |  |  |  |
| 2 | transcript:Vitvi08g01112\_t001 |  | \| \| |  | transcript:Vitvi13g00221\_t001 |  |  |  |  |  |  |  |  |
| 2 | transcript:Vitvi08g01113\_t001 |  | \| \| |  | transcript:Vitvi13g00214\_t001 |  |  |  |  |  |  |  |  |
| 2 | transcript:Vitvi08g01114\_t001 |  | \| \| |  | \| \| |  |  |  |  |  |  |  |  |
| 2 | transcript:Vitvi08g04199\_t001 |  | \| \| |  | \| \| |  |  |  |  |  |  |  |  |
| 2 | transcript:Vitvi08g04200\_t001 |  | \| \| |  | \| \| |  |  |  |  |  |  |  |  |
| 2 | transcript:Vitvi08g01116\_t001 |  | \| \| |  | \| \| |  |  |  |  |  |  |  |  |
| 2 | transcript:Vitvi08g01117\_t001 |  | \| \| |  | \| \| |  |  |  |  |  |  |  |  |
| 2 | transcript:Vitvi08g04201\_t001 |  | \| \| |  | \| \| |  |  |  |  |  |  |  |  |
| 2 | transcript:Vitvi08g01118\_t001 |  | \| \| |  | \| \| |  |  |  |  |  |  |  |  |
| 2 | transcript:Vitvi08g01119\_t001 |  | \| \| |  | \| \| |  |  |  |  |  |  |  |  |
| 2 | transcript:Vitvi08g01120\_t001 |  | transcript:Vitvi06g00382\_t001 |  | \| \| |  |  |  |  |  |  |  |  |
| 2 | transcript:Vitvi08g01121\_t001 |  | \| \| |  | \| \| |  |  |  |  |  |  |  |  |
| 2 | transcript:Vitvi08g01122\_t001 |  | \| \| |  | \| \| |  |  |  |  |  |  |  |  |
| 2 | transcript:Vitvi08g01123\_t001 |  | \| \| |  | \| \| |  |  |  |  |  |  |  |  |
| 2 | transcript:Vitvi08g02176\_t001 |  | \| \| |  | \| \| |  |  |  |  |  |  |  |  |
| 2 | transcript:Vitvi08g01124\_t001 |  | \| \| |  | transcript:Vitvi13g00210\_t002 |  |  |  |  |  |  |  |  |
| 2 | transcript:Vitvi08g04202\_t001 |  | \| \| |  | \| \| |  |  |  |  |  |  |  |  |
| 2 | transcript:Vitvi08g01125\_t001 |  | \| \| |  | \| \| |  |  |  |  |  |  |  |  |
| 2 | transcript:Vitvi08g02177\_t001 |  | \| \| |  | \| \| |  |  |  |  |  |  |  |  |
| 2 | transcript:Vitvi08g04203\_t001 |  | \| \| |  | \| \| |  |  |  |  |  |  |  |  |
| 2 | transcript:Vitvi08g01127\_t002 |  | \| \| |  | \| \| |  |  |  |  |  |  |  |  |
| 2 | transcript:Vitvi08g01129\_t001 |  | transcript:Vitvi06g00372\_t001 |  | transcript:Vitvi13g00208\_t001 |  |  |  |  |  |  |  |  |
| 2 | transcript:Vitvi08g01130\_t001 |  | \| \| |  | transcript:Vitvi13g00206\_t001 |  |  |  |  |  |  |  |  |
| 2 | transcript:Vitvi08g01133\_t002 |  | transcript:Vitvi06g00369\_t001 |  | transcript:Vitvi13g00205\_t001 |  |  |  |  |  |  |  |  |
| 2 | transcript:Vitvi08g01134\_t001 |  | \| \| |  | \| \| |  |  |  |  |  |  |  |  |
| 2 | transcript:Vitvi08g04204\_t001 |  | \| \| |  | \| \| |  |  |  |  |  |  |  |  |
| 2 | transcript:Vitvi08g04205\_t001 |  | \| \| |  | \| \| |  |  |  |  |  |  |  |  |
| 2 | transcript:Vitvi08g04206\_t001 |  | \| \| |  | \| \| |  |  |  |  |  |  |  |  |
| 2 | transcript:Vitvi08g01137\_t001 |  | \| \| |  | \| \| |  |  |  |  |  |  |  |  |
| 2 | transcript:Vitvi08g04207\_t001 |  | \| \| |  | \| \| |  |  |  |  |  |  |  |  |
| 2 | transcript:Vitvi08g01139\_t001 |  | transcript:Vitvi06g00368\_t001 |  | transcript:Vitvi13g00203\_t004 |  |  |  |  |  |  |  |  |
| 1 | transcript:Vitvi08g04208\_t001 |  | \| \| |  |  |  |  |  |  |  |  |  |  |
| 1 | transcript:Vitvi08g01140\_t002 |  | \| \| |  |  |  |  |  |  |  |  |  |  |
| 1 | transcript:Vitvi08g01142\_t003 |  | \| \| |  |  |  |  |  |  |  |  |  |  |
| 1 | transcript:Vitvi08g04209\_t001 |  | \| \| |  |  |  |  |  |  |  |  |  |  |
| 1 | transcript:Vitvi08g01143\_t002 |  | \| \| |  |  |  |  |  |  |  |  |  |  |
| 1 | transcript:Vitvi08g02180\_t001 |  | \| \| |  |  |  |  |  |  |  |  |  |  |
| 1 | transcript:Vitvi08g04210\_t001 |  | \| \| |  |  |  |  |  |  |  |  |  |  |
| 1 | transcript:Vitvi08g01144\_t001 |  | \| \| |  |  |  |  |  |  |  |  |  |  |
| 1 | transcript:Vitvi08g01146\_t001 |  | transcript:Vitvi06g00360\_t001 |  |  |  |  |  |  |  |  |  |  |
| 1 | transcript:Vitvi08g01147\_t001 |  | \| \| |  |  |  |  |  |  |  |  |  |  |
| 1 | transcript:Vitvi08g01148\_t001 |  | \| \| |  |  |  |  |  |  |  |  |  |  |
| 1 | transcript:Vitvi08g01149\_t002 |  | \| \| |  |  |  |  |  |  |  |  |  |  |
| 1 | transcript:Vitvi08g04211\_t001 |  | \| \| |  |  |  |  |  |  |  |  |  |  |
| 1 | transcript:Vitvi08g04212\_t001 |  | \| \| |  |  |  |  |  |  |  |  |  |  |
| 1 | transcript:Vitvi08g01150\_t001 |  | \| \| |  |  |  |  |  |  |  |  |  |  |
| 1 | transcript:Vitvi08g01152\_t003 |  | \| \| |  |  |  |  |  |  |  |  |  |  |
| 1 | transcript:Vitvi08g01153\_t001 |  | \| \| |  |  |  |  |  |  |  |  |  |  |
| 1 | transcript:Vitvi08g01154\_t001 |  | transcript:Vitvi06g00351\_t001 |  |  |  |  |  |  |  |  |  |  |
| 0 | transcript:Vitvi08g01155\_t001 |  |  |  |  |  |  |  |  |  |  |  |  |
| 0 | transcript:Vitvi08g01156\_t001 |  |  |  |  |  |  |  |  |  |  |  |  |
| 0 | transcript:Vitvi08g01158\_t001 |  |  |  |  |  |  |  |  |  |  |  |  |
| 0 | transcript:Vitvi08g01159\_t001 |  |  |  |  |  |  |  |  |  |  |  |  |
| 0 | transcript:Vitvi08g01161\_t003 |  |  |  |  |  |  |  |  |  |  |  |  |
| 0 | transcript:Vitvi08g01162\_t001 |  |  |  |  |  |  |  |  |  |  |  |  |
| 1 | transcript:Vitvi08g01163\_t002 |  | transcript:Vitvi06g01688\_t001 |  |  |  |  |  |  |  |  |  |  |
| 2 | transcript:Vitvi08g01164\_t001 |  | \| \| |  | transcript:Vitvi13g00263\_t001 |  |  |  |  |  |  |  |  |
| 2 | transcript:Vitvi08g01165\_t001 |  | transcript:Vitvi06g00424\_t001 |  | transcript:Vitvi13g00269\_t003 |  |  |  |  |  |  |  |  |
| 2 | transcript:Vitvi08g01166\_t001 |  | \| \| |  | \| \| |  |  |  |  |  |  |  |  |
| 2 | transcript:Vitvi08g02183\_t001 |  | \| \| |  | \| \| |  |  |  |  |  |  |  |  |
| 2 | transcript:Vitvi08g01167\_t001 |  | \| \| |  | \| \| |  |  |  |  |  |  |  |  |
| 2 | transcript:Vitvi08g02184\_t001 |  | \| \| |  | \| \| |  |  |  |  |  |  |  |  |
| 2 | transcript:Vitvi08g01168\_t001 |  | \| \| |  | \| \| |  |  |  |  |  |  |  |  |
| 2 | transcript:Vitvi08g01171\_t001 |  | \| \| |  | \| \| |  |  |  |  |  |  |  |  |
| 2 | transcript:Vitvi08g01172\_t001 |  | \| \| |  | \| \| |  |  |  |  |  |  |  |  |
| 2 | transcript:Vitvi08g01174\_t001 |  | \| \| |  | \| \| |  |  |  |  |  |  |  |  |
| 2 | transcript:Vitvi08g04213\_t001 |  | transcript:Vitvi06g04151\_t001 |  | transcript:Vitvi13g04084\_t001 |  |  |  |  |  |  |  |  |
| 2 | transcript:Vitvi08g01176\_t001 |  | transcript:Vitvi06g00434\_t001 |  | \| \| |  |  |  |  |  |  |  |  |
| 2 | transcript:Vitvi08g02185\_t001 |  | \| \| |  | \| \| |  |  |  |  |  |  |  |  |
| 2 | transcript:Vitvi08g01177\_t001 |  | \| \| |  | \| \| |  |  |  |  |  |  |  |  |
| 2 | transcript:Vitvi08g02186\_t001 |  | \| \| |  | \| \| |  |  |  |  |  |  |  |  |
| 2 | transcript:Vitvi08g01178\_t001 |  | \| \| |  | \| \| |  |  |  |  |  |  |  |  |
| 2 | transcript:Vitvi08g04214\_t001 |  | \| \| |  | \| \| |  |  |  |  |  |  |  |  |
| 2 | transcript:Vitvi08g01179\_t001 |  | transcript:Vitvi06g00436\_t001 |  | \| \| |  |  |  |  |  |  |  |  |
| 2 | transcript:Vitvi08g02188\_t001 |  | \| \| |  | \| \| |  |  |  |  |  |  |  |  |
| 2 | transcript:Vitvi08g01180\_t001 |  | transcript:Vitvi06g00439\_t003 |  | transcript:Vitvi13g00280\_t001 |  |  |  |  |  |  |  |  |
| 2 | transcript:Vitvi08g02189\_t001 |  | transcript:Vitvi06g00443\_t001 |  | transcript:Vitvi13g00281\_t001 |  |  |  |  |  |  |  |  |
| 2 | transcript:Vitvi08g01182\_t001 |  | transcript:Vitvi06g00444\_t001 |  | transcript:Vitvi13g00282\_t002 |  |  |  |  |  |  |  |  |
| 2 | transcript:Vitvi08g04215\_t001 |  | \| \| |  | \| \| |  |  |  |  |  |  |  |  |
| 2 | transcript:Vitvi08g04216\_t001 |  | \| \| |  | transcript:Vitvi13g01969\_t001 |  |  |  |  |  |  |  |  |
| 2 | transcript:Vitvi08g01184\_t001 |  | \| \| |  | \| \| |  |  |  |  |  |  |  |  |
| 2 | transcript:Vitvi08g01185\_t001 |  | \| \| |  | \| \| |  |  |  |  |  |  |  |  |
| 3 | transcript:Vitvi08g01186\_t001 |  | transcript:Vitvi06g00449\_t001 |  | \| \| |  | transcript:Vitvi13g00298\_t001 |  |  |  |  |  |  |
| 3 | transcript:Vitvi08g02190\_t001 |  | \| \| |  | \| \| |  | \| \| |  |  |  |  |  |  |
| 3 | transcript:Vitvi08g01188\_t001 |  | transcript:Vitvi06g00451\_t001 |  | \| \| |  | \| \| |  |  |  |  |  |  |
| 3 | transcript:Vitvi08g02191\_t001 |  | transcript:Vitvi06g00452\_t001 |  | \| \| |  | \| \| |  |  |  |  |  |  |
| 3 | transcript:Vitvi08g01189\_t001 |  | \| \| |  | \| \| |  | \| \| |  |  |  |  |  |  |
| 3 | transcript:Vitvi08g04217\_t001 |  | \| \| |  | \| \| |  | \| \| |  |  |  |  |  |  |
| 3 | transcript:Vitvi08g02192\_t001 |  | \| \| |  | \| \| |  | \| \| |  |  |  |  |  |  |
| 3 | transcript:Vitvi08g04218\_t001 |  | \| \| |  | \| \| |  | \| \| |  |  |  |  |  |  |
| 3 | transcript:Vitvi08g01192\_t001 |  | \| \| |  | \| \| |  | \| \| |  |  |  |  |  |  |
| 3 | transcript:Vitvi08g01193\_t001 |  | \| \| |  | \| \| |  | \| \| |  |  |  |  |  |  |
| 3 | transcript:Vitvi08g01194\_t001 |  | transcript:Vitvi06g00456\_t001 |  | \| \| |  | \| \| |  |  |  |  |  |  |
| 3 | transcript:Vitvi08g01195\_t001 |  | transcript:Vitvi06g00460\_t001 |  | \| \| |  | \| \| |  |  |  |  |  |  |
| 3 | transcript:Vitvi08g04219\_t001 |  | \| \| |  | \| \| |  | \| \| |  |  |  |  |  |  |
| 3 | transcript:Vitvi08g01196\_t001 |  | transcript:Vitvi06g00463\_t001 |  | \| \| |  | \| \| |  |  |  |  |  |  |
| 3 | transcript:Vitvi08g01197\_t001 |  | \| \| |  | \| \| |  | \| \| |  |  |  |  |  |  |
| 3 | transcript:Vitvi08g02195\_t001 |  | transcript:Vitvi06g00466\_t001 |  | \| \| |  | \| \| |  |  |  |  |  |  |
| 3 | transcript:Vitvi08g02196\_t001 |  | \| \| |  | \| \| |  | \| \| |  |  |  |  |  |  |
| 3 | transcript:Vitvi08g01201\_t003 |  | \| \| |  | \| \| |  | \| \| |  |  |  |  |  |  |
| 3 | transcript:Vitvi08g04220\_t001 |  | \| \| |  | \| \| |  | \| \| |  |  |  |  |  |  |
| 3 | transcript:Vitvi08g04221\_t001 |  | \| \| |  | \| \| |  | \| \| |  |  |  |  |  |  |
| 3 | transcript:Vitvi08g04222\_t001 |  | \| \| |  | \| \| |  | \| \| |  |  |  |  |  |  |
| 3 | transcript:Vitvi08g01204\_t004 |  | transcript:Vitvi06g00473\_t001 |  | transcript:Vitvi13g00287\_t001 |  | \| \| |  |  |  |  |  |  |
| 2 | transcript:Vitvi08g01205\_t001 |  | \| \| |  |  |  | transcript:Vitvi13g00305\_t001 |  |  |  |  |  |  |
| 2 | transcript:Vitvi08g01206\_t001 |  | transcript:Vitvi06g00481\_t001 |  |  |  | transcript:Vitvi13g00309\_t001 |  |  |  |  |  |  |
| 2 | transcript:Vitvi08g01208\_t001 |  | \| \| |  |  |  | \| \| |  |  |  |  |  |  |
| 2 | transcript:Vitvi08g01209\_t001 |  | transcript:Vitvi06g00483\_t001 |  |  |  | \| \| |  |  |  |  |  |  |
| 2 | transcript:Vitvi08g01211\_t001 |  | transcript:Vitvi06g00487\_t001 |  |  |  | \| \| |  |  |  |  |  |  |
| 2 | transcript:Vitvi08g01212\_t001 |  | \| \| |  |  |  | \| \| |  |  |  |  |  |  |
| 2 | transcript:Vitvi08g01213\_t002 |  | transcript:Vitvi06g00488\_t001 |  |  |  | transcript:Vitvi13g00310\_t001 |  |  |  |  |  |  |
| 2 | transcript:Vitvi08g04223\_t001 |  | \| \| |  |  |  | \| \| |  |  |  |  |  |  |
| 2 | transcript:Vitvi08g01214\_t001 |  | transcript:Vitvi06g01569\_t001 |  |  |  | transcript:Vitvi13g00311\_t001 |  |  |  |  |  |  |
| 2 | transcript:Vitvi08g01215\_t001 |  | \| \| |  |  |  | \| \| |  |  |  |  |  |  |
| 2 | transcript:Vitvi08g01216\_t001 |  | \| \| |  |  |  | \| \| |  |  |  |  |  |  |
| 2 | transcript:Vitvi08g01217\_t001 |  | transcript:Vitvi06g00495\_t001 |  |  |  | \| \| |  |  |  |  |  |  |
| 2 | transcript:Vitvi08g01218\_t001 |  | transcript:Vitvi06g00496\_t001 |  |  |  | \| \| |  |  |  |  |  |  |
| 2 | transcript:Vitvi08g04224\_t001 |  | \| \| |  |  |  | \| \| |  |  |  |  |  |  |
| 2 | transcript:Vitvi08g04225\_t001 |  | \| \| |  |  |  | \| \| |  |  |  |  |  |  |
| 2 | transcript:Vitvi08g04226\_t001 |  | \| \| |  |  |  | \| \| |  |  |  |  |  |  |
| 2 | transcript:Vitvi08g01221\_t001 |  | \| \| |  |  |  | \| \| |  |  |  |  |  |  |
| 2 | transcript:Vitvi08g01222\_t001 |  | \| \| |  |  |  | \| \| |  |  |  |  |  |  |
| 2 | transcript:Vitvi08g01223\_t001 |  | \| \| |  |  |  | \| \| |  |  |  |  |  |  |
| 2 | transcript:Vitvi08g04227\_t001 |  | \| \| |  |  |  | \| \| |  |  |  |  |  |  |
| 2 | transcript:Vitvi08g02198\_t001 |  | \| \| |  |  |  | \| \| |  |  |  |  |  |  |
| 2 | transcript:Vitvi08g01224\_t001 |  | \| \| |  |  |  | \| \| |  |  |  |  |  |  |
| 2 | transcript:Vitvi08g04228\_t001 |  | \| \| |  |  |  | \| \| |  |  |  |  |  |  |
| 2 | transcript:Vitvi08g04229\_t001 |  | \| \| |  |  |  | \| \| |  |  |  |  |  |  |
| 2 | transcript:Vitvi08g04230\_t001 |  | \| \| |  |  |  | \| \| |  |  |  |  |  |  |
| 2 | transcript:Vitvi08g04231\_t001 |  | \| \| |  |  |  | \| \| |  |  |  |  |  |  |
| 2 | transcript:Vitvi08g04232\_t001 |  | \| \| |  |  |  | \| \| |  |  |  |  |  |  |
| 2 | transcript:Vitvi08g02200\_t001 |  | \| \| |  |  |  | \| \| |  |  |  |  |  |  |
| 2 | transcript:Vitvi08g04233\_t001 |  | \| \| |  |  |  | transcript:Vitvi13g00322\_t001 |  |  |  |  |  |  |
| 2 | transcript:Vitvi08g02201\_t001 |  | \| \| |  |  |  | transcript:Vitvi13g00323\_t001 |  |  |  |  |  |  |
| 2 | transcript:Vitvi08g01228\_t001 |  | \| \| |  |  |  | \| \| |  |  |  |  |  |  |
| 2 | transcript:Vitvi08g04234\_t001 |  | \| \| |  |  |  | \| \| |  |  |  |  |  |  |
| 2 | transcript:Vitvi08g01229\_t001 |  | \| \| |  |  |  | \| \| |  |  |  |  |  |  |
| 2 | transcript:Vitvi08g01230\_t001 |  | \| \| |  |  |  | transcript:Vitvi13g00325\_t001 |  |  |  |  |  |  |
| 2 | transcript:Vitvi08g01231\_t001 |  | \| \| |  |  |  | \| \| |  |  |  |  |  |  |
| 2 | transcript:Vitvi08g01232\_t001 |  | transcript:Vitvi06g00502\_t001 |  |  |  | transcript:Vitvi13g00326\_t001 |  |  |  |  |  |  |
| 2 | transcript:Vitvi08g01233\_t001 |  | \| \| |  |  |  | \| \| |  |  |  |  |  |  |
| 2 | transcript:Vitvi08g01234\_t001 |  | \| \| |  |  |  | \| \| |  |  |  |  |  |  |
| 2 | transcript:Vitvi08g01235\_t001 |  | \| \| |  |  |  | transcript:Vitvi13g00329\_t001 |  |  |  |  |  |  |
| 2 | transcript:Vitvi08g01236\_t001 |  | \| \| |  |  |  | \| \| |  |  |  |  |  |  |
| 2 | transcript:Vitvi08g01237\_t001 |  | \| \| |  |  |  | transcript:Vitvi13g00330\_t001 |  |  |  |  |  |  |
| 2 | transcript:Vitvi08g01238\_t001 |  | \| \| |  |  |  | \| \| |  |  |  |  |  |  |
| 2 | transcript:Vitvi08g04235\_t001 |  | \| \| |  |  |  | \| \| |  |  |  |  |  |  |
| 2 | transcript:Vitvi08g01239\_t001 |  | \| \| |  |  |  | \| \| |  |  |  |  |  |  |
| 2 | transcript:Vitvi08g02202\_t001 |  | \| \| |  |  |  | \| \| |  |  |  |  |  |  |
| 2 | transcript:Vitvi08g01240\_t001 |  | \| \| |  |  |  | \| \| |  |  |  |  |  |  |
| 2 | transcript:Vitvi08g01241\_t001 |  | transcript:Vitvi06g00512\_t001 |  |  |  | transcript:Vitvi13g00332\_t001 |  |  |  |  |  |  |
| 2 | transcript:Vitvi08g01242\_t001 |  | \| \| |  |  |  | \| \| |  |  |  |  |  |  |
| 2 | transcript:Vitvi08g01243\_t001 |  | \| \| |  |  |  | \| \| |  |  |  |  |  |  |
| 2 | transcript:Vitvi08g04236\_t001 |  | \| \| |  |  |  | \| \| |  |  |  |  |  |  |
| 2 | transcript:Vitvi08g01245\_t001 |  | transcript:Vitvi06g00513\_t001 |  |  |  | transcript:Vitvi13g01994\_t001 |  |  |  |  |  |  |
| 2 | transcript:Vitvi08g01246\_t001 |  | \| \| |  |  |  | \| \| |  |  |  |  |  |  |
| 2 | transcript:Vitvi08g01247\_t001 |  | \| \| |  |  |  | \| \| |  |  |  |  |  |  |
| 2 | transcript:Vitvi08g01248\_t001 |  | transcript:Vitvi06g00520\_t001 |  |  |  | transcript:Vitvi13g01996\_t001 |  |  |  |  |  |  |
| 2 | transcript:Vitvi08g02203\_t001 |  | transcript:Vitvi06g01710\_t001 |  |  |  | transcript:Vitvi13g00340\_t001 |  |  |  |  |  |  |
| 2 | transcript:Vitvi08g01249\_t001 |  | transcript:Vitvi06g00521\_t001 |  |  |  | \| \| |  |  |  |  |  |  |
| 2 | transcript:Vitvi08g01250\_t001 |  | transcript:Vitvi06g01718\_t001 |  |  |  | \| \| |  |  |  |  |  |  |
| 2 | transcript:Vitvi08g04237\_t001 |  | \| \| |  |  |  | \| \| |  |  |  |  |  |  |
| 2 | transcript:Vitvi08g01252\_t001 |  | \| \| |  |  |  | \| \| |  |  |  |  |  |  |
| 2 | transcript:Vitvi08g04238\_t001 |  | transcript:Vitvi06g04172\_t001 |  |  |  | \| \| |  |  |  |  |  |  |
| 2 | transcript:Vitvi08g02204\_t001 |  | \| \| |  |  |  | transcript:Vitvi13g01999\_t001 |  |  |  |  |  |  |
| 2 | transcript:Vitvi08g01253\_t001 |  | \| \| |  |  |  | \| \| |  |  |  |  |  |  |
| 2 | transcript:Vitvi08g02205\_t001 |  | \| \| |  |  |  | \| \| |  |  |  |  |  |  |
| 2 | transcript:Vitvi08g01254\_t002 |  | \| \| |  |  |  | \| \| |  |  |  |  |  |  |
| 2 | transcript:Vitvi08g04239\_t001 |  | \| \| |  |  |  | \| \| |  |  |  |  |  |  |
| 2 | transcript:Vitvi08g02206\_t001 |  | transcript:Vitvi06g04177\_t001 |  |  |  | transcript:Vitvi13g04110\_t001 |  |  |  |  |  |  |
| 2 | transcript:Vitvi08g01255\_t001 |  | \| \| |  |  |  | transcript:Vitvi13g02001\_t001 |  |  |  |  |  |  |
| 2 | transcript:Vitvi08g04240\_t001 |  | \| \| |  |  |  | \| \| |  |  |  |  |  |  |
| 2 | transcript:Vitvi08g01256\_t001 |  | transcript:Vitvi06g00535\_t001 |  |  |  | transcript:Vitvi13g02003\_t001 |  |  |  |  |  |  |
| 1 | transcript:Vitvi08g01257\_t001 |  |  |  |  |  | transcript:Vitvi13g00353\_t001 |  |  |  |  |  |  |
| 1 | transcript:Vitvi08g04241\_t001 |  |  |  |  |  | \| \| |  |  |  |  |  |  |
| 1 | transcript:Vitvi08g01259\_t001 |  |  |  |  |  | \| \| |  |  |  |  |  |  |
| 1 | transcript:Vitvi08g02209\_t001 |  |  |  |  |  | \| \| |  |  |  |  |  |  |
| 1 | transcript:Vitvi08g02210\_t001 |  |  |  |  |  | \| \| |  |  |  |  |  |  |
| 1 | transcript:Vitvi08g01260\_t001 |  |  |  |  |  | \| \| |  |  |  |  |  |  |
| 1 | transcript:Vitvi08g01261\_t001 |  |  |  |  |  | transcript:Vitvi13g00355\_t001 |  |  |  |  |  |  |
| 1 | transcript:Vitvi08g01262\_t001 |  |  |  |  |  | \| \| |  |  |  |  |  |  |
| 1 | transcript:Vitvi08g04242\_t001 |  |  |  |  |  | \| \| |  |  |  |  |  |  |
| 1 | transcript:Vitvi08g01264\_t001 |  |  |  |  |  | \| \| |  |  |  |  |  |  |
| 1 | transcript:Vitvi08g04243\_t001 |  |  |  |  |  | \| \| |  |  |  |  |  |  |
| 1 | transcript:Vitvi08g01265\_t001 |  |  |  |  |  | \| \| |  |  |  |  |  |  |
| 1 | transcript:Vitvi08g02211\_t001 |  |  |  |  |  | \| \| |  |  |  |  |  |  |
| 1 | transcript:Vitvi08g02212\_t001 |  |  |  |  |  | \| \| |  |  |  |  |  |  |
| 1 | transcript:Vitvi08g04244\_t001 |  |  |  |  |  | \| \| |  |  |  |  |  |  |
| 1 | transcript:Vitvi08g01266\_t002 |  |  |  |  |  | \| \| |  |  |  |  |  |  |
| 1 | transcript:Vitvi08g01267\_t001 |  |  |  |  |  | \| \| |  |  |  |  |  |  |
| 1 | transcript:Vitvi08g02214\_t001 |  |  |  |  |  | \| \| |  |  |  |  |  |  |
| 1 | transcript:Vitvi08g04245\_t001 |  |  |  |  |  | \| \| |  |  |  |  |  |  |
| 1 | transcript:Vitvi08g04246\_t001 |  |  |  |  |  | \| \| |  |  |  |  |  |  |
| 1 | transcript:Vitvi08g02216\_t001 |  |  |  |  |  | \| \| |  |  |  |  |  |  |
| 1 | transcript:Vitvi08g04247\_t001 |  |  |  |  |  | \| \| |  |  |  |  |  |  |
| 1 | transcript:Vitvi08g04248\_t001 |  |  |  |  |  | \| \| |  |  |  |  |  |  |
| 1 | transcript:Vitvi08g02217\_t001 |  |  |  |  |  | \| \| |  |  |  |  |  |  |
| 1 | transcript:Vitvi08g02218\_t001 |  |  |  |  |  | \| \| |  |  |  |  |  |  |
| 1 | transcript:Vitvi08g04249\_t001 |  |  |  |  |  | \| \| |  |  |  |  |  |  |
| 1 | transcript:Vitvi08g04250\_t001 |  |  |  |  |  | \| \| |  |  |  |  |  |  |
| 1 | transcript:Vitvi08g02220\_t001 |  |  |  |  |  | \| \| |  |  |  |  |  |  |
| 1 | transcript:Vitvi08g02221\_t001 |  |  |  |  |  | \| \| |  |  |  |  |  |  |
| 1 | transcript:Vitvi08g01270\_t001 |  |  |  |  |  | \| \| |  |  |  |  |  |  |
| 1 | transcript:Vitvi08g01272\_t001 |  |  |  |  |  | \| \| |  |  |  |  |  |  |
| 1 | transcript:Vitvi08g02222\_t001 |  |  |  |  |  | \| \| |  |  |  |  |  |  |
| 1 | transcript:Vitvi08g01273\_t001 |  |  |  |  |  | transcript:Vitvi13g00365\_t001 |  |  |  |  |  |  |
| 1 | transcript:Vitvi08g01274\_t001 |  |  |  |  |  | transcript:Vitvi13g00366\_t001 |  |  |  |  |  |  |
| 1 | transcript:Vitvi08g01276\_t001 |  |  |  |  |  | \| \| |  |  |  |  |  |  |
| 1 | transcript:Vitvi08g04251\_t001 |  |  |  |  |  | transcript:Vitvi13g00367\_t001 |  |  |  |  |  |  |
| 0 | transcript:Vitvi08g01278\_t001 |  |  |  |  |  |  |  |  |  |  |  |  |
| 0 | transcript:Vitvi08g02223\_t001 |  |  |  |  |  |  |  |  |  |  |  |  |
| 0 | transcript:Vitvi08g01280\_t001 |  |  |  |  |  |  |  |  |  |  |  |  |
| 0 | transcript:Vitvi08g04252\_t001 |  |  |  |  |  |  |  |  |  |  |  |  |
| 1 | transcript:Vitvi08g04253\_t006 |  | transcript:Vitvi06g01723\_t001 |  |  |  |  |  |  |  |  |  |  |
| 1 | transcript:Vitvi08g01281\_t001 |  | \| \| |  |  |  |  |  |  |  |  |  |  |
| 1 | transcript:Vitvi08g01282\_t001 |  | \| \| |  |  |  |  |  |  |  |  |  |  |
| 1 | transcript:Vitvi08g01283\_t001 |  | \| \| |  |  |  |  |  |  |  |  |  |  |
| 1 | transcript:Vitvi08g02226\_t002 |  | transcript:Vitvi06g01724\_t001 |  |  |  |  |  |  |  |  |  |  |
| 1 | transcript:Vitvi08g02228\_t001 |  | transcript:Vitvi06g04187\_t001 |  |  |  |  |  |  |  |  |  |  |
| 1 | transcript:Vitvi08g04254\_t001 |  | \| \| |  |  |  |  |  |  |  |  |  |  |
| 1 | transcript:Vitvi08g01285\_t001 |  | transcript:Vitvi06g00556\_t001 |  |  |  |  |  |  |  |  |  |  |
| 1 | transcript:Vitvi08g01287\_t001 |  | transcript:Vitvi06g00558\_t001 |  |  |  |  |  |  |  |  |  |  |
| 1 | transcript:Vitvi08g01288\_t002 |  | \| \| |  |  |  |  |  |  |  |  |  |  |
| 1 | transcript:Vitvi08g02230\_t001 |  | transcript:Vitvi06g01729\_t001 |  |  |  |  |  |  |  |  |  |  |
| 1 | transcript:Vitvi08g04255\_t001 |  | \| \| |  |  |  |  |  |  |  |  |  |  |
| 1 | transcript:Vitvi08g01290\_t001 |  | transcript:Vitvi06g00559\_t001 |  |  |  |  |  |  |  |  |  |  |
| 1 | transcript:Vitvi08g01291\_t001 |  | \| \| |  |  |  |  |  |  |  |  |  |  |
| 1 | transcript:Vitvi08g04256\_t001 |  | \| \| |  |  |  |  |  |  |  |  |  |  |
| 2 | transcript:Vitvi08g01292\_t001 |  | \| \| |  | transcript:Vitvi13g04138\_t001 |  |  |  |  |  |  |  |  |
| 2 | transcript:Vitvi08g04257\_t001 |  | \| \| |  | \| \| |  |  |  |  |  |  |  |  |
| 2 | transcript:Vitvi08g04258\_t001 |  | \| \| |  | \| \| |  |  |  |  |  |  |  |  |
| 2 | transcript:Vitvi08g01293\_t001 |  | transcript:Vitvi06g00563\_t001 |  | \| \| |  |  |  |  |  |  |  |  |
| 2 | transcript:Vitvi08g01294\_t001 |  | \| \| |  | \| \| |  |  |  |  |  |  |  |  |
| 2 | transcript:Vitvi08g01295\_t001 |  | transcript:Vitvi06g00564\_t001 |  | \| \| |  |  |  |  |  |  |  |  |
| 2 | transcript:Vitvi08g01296\_t001 |  | transcript:Vitvi06g00565\_t001 |  | \| \| |  |  |  |  |  |  |  |  |
| 2 | transcript:Vitvi08g01297\_t001 |  | transcript:Vitvi06g00567\_t001 |  | transcript:Vitvi13g04140\_t003 |  |  |  |  |  |  |  |  |
| 2 | transcript:Vitvi08g04259\_t001 |  | \| \| |  | \| \| |  |  |  |  |  |  |  |  |
| 2 | transcript:Vitvi08g01298\_t001 |  | \| \| |  | transcript:Vitvi13g00494\_t001 |  |  |  |  |  |  |  |  |
| 2 | transcript:Vitvi08g01299\_t001 |  | \| \| |  | \| \| |  |  |  |  |  |  |  |  |
| 2 | transcript:Vitvi08g01300\_t001 |  | transcript:Vitvi06g01734\_t001 |  | \| \| |  |  |  |  |  |  |  |  |
| 2 | transcript:Vitvi08g01301\_t001 |  | \| \| |  | \| \| |  |  |  |  |  |  |  |  |
| 2 | transcript:Vitvi08g04260\_t001 |  | \| \| |  | \| \| |  |  |  |  |  |  |  |  |
| 2 | transcript:Vitvi08g01303\_t001 |  | \| \| |  | \| \| |  |  |  |  |  |  |  |  |
| 2 | transcript:Vitvi08g01304\_t001 |  | \| \| |  | \| \| |  |  |  |  |  |  |  |  |
| 2 | transcript:Vitvi08g04261\_t001 |  | \| \| |  | \| \| |  |  |  |  |  |  |  |  |
| 2 | transcript:Vitvi08g01305\_t001 |  | \| \| |  | transcript:Vitvi13g00495\_t001 |  |  |  |  |  |  |  |  |
| 2 | transcript:Vitvi08g02232\_t002 |  | transcript:Vitvi06g00569\_t001 |  | \| \| |  |  |  |  |  |  |  |  |
| 2 | transcript:Vitvi08g01307\_t001 |  | \| \| |  | \| \| |  |  |  |  |  |  |  |  |
| 2 | transcript:Vitvi08g02233\_t001 |  | \| \| |  | \| \| |  |  |  |  |  |  |  |  |
| 2 | transcript:Vitvi08g04262\_t001 |  | \| \| |  | \| \| |  |  |  |  |  |  |  |  |
| 2 | transcript:Vitvi08g01308\_t001 |  | \| \| |  | transcript:Vitvi13g00499\_t001 |  |  |  |  |  |  |  |  |
| 2 | transcript:Vitvi08g01309\_t002 |  | \| \| |  | \| \| |  |  |  |  |  |  |  |  |
| 2 | transcript:Vitvi08g04263\_t001 |  | \| \| |  | transcript:Vitvi13g04142\_t001 |  |  |  |  |  |  |  |  |
| 2 | transcript:Vitvi08g04264\_t001 |  | \| \| |  | \| \| |  |  |  |  |  |  |  |  |
| 2 | transcript:Vitvi08g01311\_t001 |  | transcript:Vitvi06g00573\_t001 |  | \| \| |  |  |  |  |  |  |  |  |
| 2 | transcript:Vitvi08g04265\_t001 |  | \| \| |  | \| \| |  |  |  |  |  |  |  |  |
| 2 | transcript:Vitvi08g01312\_t001 |  | transcript:Vitvi06g00575\_t001 |  | transcript:Vitvi13g00501\_t001 |  |  |  |  |  |  |  |  |
| 2 | transcript:Vitvi08g01313\_t001 |  | transcript:Vitvi06g00577\_t001.1.6037826e |  | \| \| |  |  |  |  |  |  |  |  |
| 2 | transcript:Vitvi08g04266\_t001 |  | \| \| |  | \| \| |  |  |  |  |  |  |  |  |
| 2 | transcript:Vitvi08g01314\_t001 |  | \| \| |  | \| \| |  |  |  |  |  |  |  |  |
| 2 | transcript:Vitvi08g01315\_t001 |  | \| \| |  | \| \| |  |  |  |  |  |  |  |  |
| 2 | transcript:Vitvi08g01316\_t001 |  | \| \| |  | \| \| |  |  |  |  |  |  |  |  |
| 2 | transcript:Vitvi08g01318\_t001 |  | \| \| |  | \| \| |  |  |  |  |  |  |  |  |
| 2 | transcript:Vitvi08g01319\_t001 |  | \| \| |  | \| \| |  |  |  |  |  |  |  |  |
| 2 | transcript:Vitvi08g01320\_t001 |  | \| \| |  | \| \| |  |  |  |  |  |  |  |  |
| 2 | transcript:Vitvi08g01321\_t001 |  | \| \| |  | \| \| |  |  |  |  |  |  |  |  |
| 2 | transcript:Vitvi08g01322\_t001 |  | \| \| |  | \| \| |  |  |  |  |  |  |  |  |
| 2 | transcript:Vitvi08g01323\_t001 |  | \| \| |  | \| \| |  |  |  |  |  |  |  |  |
| 2 | transcript:Vitvi08g04267\_t001 |  | \| \| |  | \| \| |  |  |  |  |  |  |  |  |
| 2 | transcript:Vitvi08g01325\_t001 |  | transcript:Vitvi06g00585\_t001 |  | \| \| |  |  |  |  |  |  |  |  |
| 2 | transcript:Vitvi08g01326\_t001 |  | \| \| |  | \| \| |  |  |  |  |  |  |  |  |
| 2 | transcript:Vitvi08g01327\_t001 |  | transcript:Vitvi06g00587\_t001 |  | \| \| |  |  |  |  |  |  |  |  |
| 2 | transcript:Vitvi08g04268\_t001 |  | \| \| |  | \| \| |  |  |  |  |  |  |  |  |
| 2 | transcript:Vitvi08g01329\_t001 |  | transcript:Vitvi06g00588\_t001 |  | \| \| |  |  |  |  |  |  |  |  |
| 2 | transcript:Vitvi08g04269\_t001 |  | \| \| |  | \| \| |  |  |  |  |  |  |  |  |
| 2 | transcript:Vitvi08g04270\_t001 |  | \| \| |  | \| \| |  |  |  |  |  |  |  |  |
| 2 | transcript:Vitvi08g04271\_t001 |  | \| \| |  | \| \| |  |  |  |  |  |  |  |  |
| 2 | transcript:Vitvi08g04272\_t001 |  | \| \| |  | \| \| |  |  |  |  |  |  |  |  |
| 2 | transcript:Vitvi08g01332\_t001 |  | \| \| |  | \| \| |  |  |  |  |  |  |  |  |
| 2 | transcript:Vitvi08g01333\_t001 |  | transcript:Vitvi06g00590\_t001 |  | \| \| |  |  |  |  |  |  |  |  |
| 2 | transcript:Vitvi08g04273\_t001 |  | \| \| |  | \| \| |  |  |  |  |  |  |  |  |
| 2 | transcript:Vitvi08g01334\_t001 |  | \| \| |  | \| \| |  |  |  |  |  |  |  |  |
| 2 | transcript:Vitvi08g01335\_t001 |  | transcript:Vitvi06g00591\_t001 |  | transcript:Vitvi13g00509\_t001 |  |  |  |  |  |  |  |  |
| 2 | transcript:Vitvi08g01336\_t001 |  | transcript:Vitvi06g00592\_t001 |  | transcript:Vitvi13g00510\_t001 |  |  |  |  |  |  |  |  |
| 2 | transcript:Vitvi08g01337\_t001 |  | \| \| |  | transcript:Vitvi13g00517\_t001 |  |  |  |  |  |  |  |  |
| 2 | transcript:Vitvi08g01338\_t001 |  | \| \| |  | \| \| |  |  |  |  |  |  |  |  |
| 2 | transcript:Vitvi08g02235\_t001 |  | transcript:Vitvi06g04202\_t001 |  | \| \| |  |  |  |  |  |  |  |  |
| 1 | transcript:Vitvi08g01339\_t001 |  |  |  | \| \| |  |  |  |  |  |  |  |  |
| 1 | transcript:Vitvi08g02236\_t001 |  |  |  | \| \| |  |  |  |  |  |  |  |  |
| 1 | transcript:Vitvi08g01340\_t001 |  |  |  | transcript:Vitvi13g00522\_t001 |  |  |  |  |  |  |  |  |
| 0 | transcript:Vitvi08g04274\_t001 |  |  |  |  |  |  |  |  |  |  |  |  |
| 0 | transcript:Vitvi08g04275\_t001 |  |  |  |  |  |  |  |  |  |  |  |  |
| 0 | transcript:Vitvi08g01341\_t001 |  |  |  |  |  |  |  |  |  |  |  |  |
| 0 | transcript:Vitvi08g01342\_t001 |  |  |  |  |  |  |  |  |  |  |  |  |
| 0 | transcript:Vitvi08g04276\_t001 |  |  |  |  |  |  |  |  |  |  |  |  |
| 0 | transcript:Vitvi08g04277\_t001 |  |  |  |  |  |  |  |  |  |  |  |  |
| 0 | transcript:Vitvi08g02238\_t001 |  |  |  |  |  |  |  |  |  |  |  |  |
| 0 | transcript:Vitvi08g04278\_t001 |  |  |  |  |  |  |  |  |  |  |  |  |
| 1 | transcript:Vitvi08g01346\_t001 |  | transcript:Vitvi13g00019\_t001 |  |  |  |  |  |  |  |  |  |  |
| 1 | transcript:Vitvi08g01347\_t001 |  | \| \| |  |  |  |  |  |  |  |  |  |  |
| 1 | transcript:Vitvi08g01348\_t001 |  | \| \| |  |  |  |  |  |  |  |  |  |  |
| 1 | transcript:Vitvi08g02239\_t001 |  | \| \| |  |  |  |  |  |  |  |  |  |  |
| 1 | transcript:Vitvi08g04279\_t001 |  | \| \| |  |  |  |  |  |  |  |  |  |  |
| 1 | transcript:Vitvi08g02241\_t001 |  | \| \| |  |  |  |  |  |  |  |  |  |  |
| 1 | transcript:Vitvi08g02242\_t001 |  | \| \| |  |  |  |  |  |  |  |  |  |  |
| 1 | transcript:Vitvi08g02243\_t002 |  | \| \| |  |  |  |  |  |  |  |  |  |  |
| 1 | transcript:Vitvi08g01349\_t001 |  | \| \| |  |  |  |  |  |  |  |  |  |  |
| 1 | transcript:Vitvi08g01351\_t001 |  | transcript:Vitvi13g00025\_t001 |  |  |  |  |  |  |  |  |  |  |
| 1 | transcript:Vitvi08g01352\_t001 |  | transcript:Vitvi13g04003\_t001 |  |  |  |  |  |  |  |  |  |  |
| 1 | transcript:Vitvi08g01353\_t001 |  | \| \| |  |  |  |  |  |  |  |  |  |  |
| 1 | transcript:Vitvi08g01354\_t001 |  | \| \| |  |  |  |  |  |  |  |  |  |  |
| 1 | transcript:Vitvi08g01355\_t001 |  | transcript:Vitvi13g00027\_t001 |  |  |  |  |  |  |  |  |  |  |
| 1 | transcript:Vitvi08g01356\_t001 |  | \| \| |  |  |  |  |  |  |  |  |  |  |
| 1 | transcript:Vitvi08g01357\_t001 |  | transcript:Vitvi13g00028\_t001 |  |  |  |  |  |  |  |  |  |  |
| 1 | transcript:Vitvi08g04280\_t001 |  | \| \| |  |  |  |  |  |  |  |  |  |  |
| 1 | transcript:Vitvi08g01358\_t001 |  | transcript:Vitvi13g01886\_t002 |  |  |  |  |  |  |  |  |  |  |
| 1 | transcript:Vitvi08g01359\_t003 |  | \| \| |  |  |  |  |  |  |  |  |  |  |
| 1 | transcript:Vitvi08g01360\_t001 |  | \| \| |  |  |  |  |  |  |  |  |  |  |
| 1 | transcript:Vitvi08g04281\_t001 |  | \| \| |  |  |  |  |  |  |  |  |  |  |
| 1 | transcript:Vitvi08g02244\_t003 |  | transcript:Vitvi13g00031\_t001 |  |  |  |  |  |  |  |  |  |  |
| 1 | transcript:Vitvi08g01361\_t006 |  | \| \| |  |  |  |  |  |  |  |  |  |  |
| 1 | transcript:Vitvi08g01363\_t001 |  | \| \| |  |  |  |  |  |  |  |  |  |  |
| 1 | transcript:Vitvi08g01364\_t001 |  | \| \| |  |  |  |  |  |  |  |  |  |  |
| 1 | transcript:Vitvi08g02245\_t002 |  | \| \| |  |  |  |  |  |  |  |  |  |  |
| 1 | transcript:Vitvi08g04282\_t001 |  | \| \| |  |  |  |  |  |  |  |  |  |  |
| 1 | transcript:Vitvi08g01365\_t001 |  | \| \| |  |  |  |  |  |  |  |  |  |  |
| 1 | transcript:Vitvi08g01366\_t001 |  | transcript:Vitvi13g00037\_t001 |  |  |  |  |  |  |  |  |  |  |
| 1 | transcript:Vitvi08g01367\_t001 |  | \| \| |  |  |  |  |  |  |  |  |  |  |
| 1 | transcript:Vitvi08g01369\_t001 |  | transcript:Vitvi13g00047\_t001 |  |  |  |  |  |  |  |  |  |  |
| 0 | transcript:Vitvi08g01370\_t001 |  |  |  |  |  |  |  |  |  |  |  |  |
| 0 | transcript:Vitvi08g01371\_t001 |  |  |  |  |  |  |  |  |  |  |  |  |
| 0 | transcript:Vitvi08g01372\_t001 |  |  |  |  |  |  |  |  |  |  |  |  |
| 0 | transcript:Vitvi08g01373\_t001 |  |  |  |  |  |  |  |  |  |  |  |  |
| 0 | transcript:Vitvi08g01374\_t001 |  |  |  |  |  |  |  |  |  |  |  |  |
| 0 | transcript:Vitvi08g01375\_t001 |  |  |  |  |  |  |  |  |  |  |  |  |
| 0 | transcript:Vitvi08g04283\_t001 |  |  |  |  |  |  |  |  |  |  |  |  |
| 1 | transcript:Vitvi08g01376\_t001 |  | transcript:Vitvi06g01559\_t001 |  |  |  |  |  |  |  |  |  |  |
| 1 | transcript:Vitvi08g01377\_t001 |  | \| \| |  |  |  |  |  |  |  |  |  |  |
| 1 | transcript:Vitvi08g02246\_t001 |  | \| \| |  |  |  |  |  |  |  |  |  |  |
| 1 | transcript:Vitvi08g01378\_t001 |  | \| \| |  |  |  |  |  |  |  |  |  |  |
| 1 | transcript:Vitvi08g01379\_t001 |  | \| \| |  |  |  |  |  |  |  |  |  |  |
| 1 | transcript:Vitvi08g04284\_t001 |  | transcript:Vitvi06g01556\_t001 |  |  |  |  |  |  |  |  |  |  |
| 1 | transcript:Vitvi08g04285\_t001 |  | \| \| |  |  |  |  |  |  |  |  |  |  |
| 1 | transcript:Vitvi08g04286\_t001 |  | \| \| |  |  |  |  |  |  |  |  |  |  |
| 1 | transcript:Vitvi08g04287\_t001 |  | \| \| |  |  |  |  |  |  |  |  |  |  |
| 1 | transcript:Vitvi08g04288\_t001 |  | \| \| |  |  |  |  |  |  |  |  |  |  |
| 1 | transcript:Vitvi08g02249\_t001 |  | \| \| |  |  |  |  |  |  |  |  |  |  |
| 1 | transcript:Vitvi08g04289\_t001 |  | \| \| |  |  |  |  |  |  |  |  |  |  |
| 1 | transcript:Vitvi08g04290\_t001 |  | \| \| |  |  |  |  |  |  |  |  |  |  |
| 1 | transcript:Vitvi08g01382\_t001 |  | \| \| |  |  |  |  |  |  |  |  |  |  |
| 1 | transcript:Vitvi08g04291\_t001 |  | \| \| |  |  |  |  |  |  |  |  |  |  |
| 1 | transcript:Vitvi08g01384\_t001 |  | \| \| |  |  |  |  |  |  |  |  |  |  |
| 1 | transcript:Vitvi08g01385\_t001 |  | \| \| |  |  |  |  |  |  |  |  |  |  |
| 1 | transcript:Vitvi08g04292\_t001 |  | \| \| |  |  |  |  |  |  |  |  |  |  |
| 1 | transcript:Vitvi08g01386\_t001 |  | \| \| |  |  |  |  |  |  |  |  |  |  |
| 1 | transcript:Vitvi08g01387\_t001 |  | \| \| |  |  |  |  |  |  |  |  |  |  |
| 1 | transcript:Vitvi08g01388\_t001 |  | transcript:Vitvi06g01552\_t001 |  |  |  |  |  |  |  |  |  |  |
| 1 | transcript:Vitvi08g01389\_t001 |  | \| \| |  |  |  |  |  |  |  |  |  |  |
| 1 | transcript:Vitvi08g02251\_t001.1.6037826f |  | \| \| |  |  |  |  |  |  |  |  |  |  |
| 1 | transcript:Vitvi08g01390\_t001 |  | \| \| |  |  |  |  |  |  |  |  |  |  |
| 1 | transcript:Vitvi08g01391\_t001 |  | transcript:Vitvi06g01549\_t001 |  |  |  |  |  |  |  |  |  |  |
| 1 | transcript:Vitvi08g01392\_t001 |  | transcript:Vitvi06g01548\_t001 |  |  |  |  |  |  |  |  |  |  |
| 1 | transcript:Vitvi08g01393\_t001 |  | transcript:Vitvi06g01546\_t001 |  |  |  |  |  |  |  |  |  |  |
| 1 | transcript:Vitvi08g01394\_t001 |  | transcript:Vitvi06g01545\_t001 |  |  |  |  |  |  |  |  |  |  |
| 1 | transcript:Vitvi08g02252\_t001 |  | \| \| |  |  |  |  |  |  |  |  |  |  |
| 1 | transcript:Vitvi08g01395\_t001 |  | transcript:Vitvi06g01543\_t001 |  |  |  |  |  |  |  |  |  |  |
| 1 | transcript:Vitvi08g01396\_t001 |  | \| \| |  |  |  |  |  |  |  |  |  |  |
| 1 | transcript:Vitvi08g01397\_t001 |  | \| \| |  |  |  |  |  |  |  |  |  |  |
| 1 | transcript:Vitvi08g02253\_t001 |  | \| \| |  |  |  |  |  |  |  |  |  |  |
| 1 | transcript:Vitvi08g02254\_t001 |  | \| \| |  |  |  |  |  |  |  |  |  |  |
| 1 | transcript:Vitvi08g04293\_t001 |  | \| \| |  |  |  |  |  |  |  |  |  |  |
| 1 | transcript:Vitvi08g04294\_t001 |  | \| \| |  |  |  |  |  |  |  |  |  |  |
| 1 | transcript:Vitvi08g04295\_t001 |  | \| \| |  |  |  |  |  |  |  |  |  |  |
| 1 | transcript:Vitvi08g01399\_t001 |  | \| \| |  |  |  |  |  |  |  |  |  |  |
| 1 | transcript:Vitvi08g02255\_t001 |  | transcript:Vitvi06g01537\_t001 |  |  |  |  |  |  |  |  |  |  |
| 1 | transcript:Vitvi08g01401\_t001 |  | \| \| |  |  |  |  |  |  |  |  |  |  |
| 1 | transcript:Vitvi08g04296\_t001 |  | \| \| |  |  |  |  |  |  |  |  |  |  |
| 1 | transcript:Vitvi08g01402\_t001 |  | \| \| |  |  |  |  |  |  |  |  |  |  |
| 1 | transcript:Vitvi08g01403\_t001 |  | \| \| |  |  |  |  |  |  |  |  |  |  |
| 1 | transcript:Vitvi08g04297\_t001 |  | \| \| |  |  |  |  |  |  |  |  |  |  |
| 1 | transcript:Vitvi08g04298\_t001 |  | \| \| |  |  |  |  |  |  |  |  |  |  |
| 1 | transcript:Vitvi08g04299\_t001 |  | \| \| |  |  |  |  |  |  |  |  |  |  |
| 1 | transcript:Vitvi08g01406\_t002 |  | \| \| |  |  |  |  |  |  |  |  |  |  |
| 1 | transcript:Vitvi08g01408\_t001 |  | \| \| |  |  |  |  |  |  |  |  |  |  |
| 1 | transcript:Vitvi08g01409\_t001 |  | \| \| |  |  |  |  |  |  |  |  |  |  |
| 1 | transcript:Vitvi08g01410\_t002 |  | transcript:Vitvi06g01532\_t001 |  |  |  |  |  |  |  |  |  |  |
| 1 | transcript:Vitvi08g01411\_t001 |  | \| \| |  |  |  |  |  |  |  |  |  |  |
| 1 | transcript:Vitvi08g04300\_t001 |  | \| \| |  |  |  |  |  |  |  |  |  |  |
| 1 | transcript:Vitvi08g01412\_t001 |  | \| \| |  |  |  |  |  |  |  |  |  |  |
| 1 | transcript:Vitvi08g01413\_t001 |  | \| \| |  |  |  |  |  |  |  |  |  |  |
| 1 | transcript:Vitvi08g04301\_t001 |  | \| \| |  |  |  |  |  |  |  |  |  |  |
| 1 | transcript:Vitvi08g01414\_t001 |  | \| \| |  |  |  |  |  |  |  |  |  |  |
| 1 | transcript:Vitvi08g01415\_t001 |  | \| \| |  |  |  |  |  |  |  |  |  |  |
| 1 | transcript:Vitvi08g01416\_t001 |  | transcript:Vitvi06g01530\_t001 |  |  |  |  |  |  |  |  |  |  |
| 1 | transcript:Vitvi08g02257\_t001 |  | transcript:Vitvi06g01974\_t001 |  |  |  |  |  |  |  |  |  |  |
| 1 | transcript:Vitvi08g04302\_t001 |  | \| \| |  |  |  |  |  |  |  |  |  |  |
| 1 | transcript:Vitvi08g01417\_t001 |  | transcript:Vitvi06g01524\_t002 |  |  |  |  |  |  |  |  |  |  |
| 1 | transcript:Vitvi08g01418\_t001 |  | transcript:Vitvi06g01522\_t001 |  |  |  |  |  |  |  |  |  |  |
| 1 | transcript:Vitvi08g01419\_t001 |  | transcript:Vitvi06g01521\_t001 |  |  |  |  |  |  |  |  |  |  |
| 1 | transcript:Vitvi08g04303\_t001 |  | \| \| |  |  |  |  |  |  |  |  |  |  |
| 1 | transcript:Vitvi08g01421\_t001 |  | \| \| |  |  |  |  |  |  |  |  |  |  |
| 1 | transcript:Vitvi08g01422\_t001 |  | \| \| |  |  |  |  |  |  |  |  |  |  |
| 1 | transcript:Vitvi08g01423\_t001 |  | \| \| |  |  |  |  |  |  |  |  |  |  |
| 1 | transcript:Vitvi08g01425\_t001 |  | \| \| |  |  |  |  |  |  |  |  |  |  |
| 1 | transcript:Vitvi08g01426\_t001 |  | transcript:Vitvi06g01515\_t001 |  |  |  |  |  |  |  |  |  |  |
| 1 | transcript:Vitvi08g04304\_t001 |  | \| \| |  |  |  |  |  |  |  |  |  |  |
| 1 | transcript:Vitvi08g01427\_t001 |  | transcript:Vitvi06g01512\_t001 |  |  |  |  |  |  |  |  |  |  |
| 1 | transcript:Vitvi08g01428\_t001 |  | \| \| |  |  |  |  |  |  |  |  |  |  |
| 1 | transcript:Vitvi08g02258\_t001 |  | \| \| |  |  |  |  |  |  |  |  |  |  |
| 1 | transcript:Vitvi08g02259\_t001 |  | \| \| |  |  |  |  |  |  |  |  |  |  |
| 1 | transcript:Vitvi08g02260\_t001 |  | \| \| |  |  |  |  |  |  |  |  |  |  |
| 1 | transcript:Vitvi08g01429\_t001 |  | \| \| |  |  |  |  |  |  |  |  |  |  |
| 1 | transcript:Vitvi08g01430\_t001 |  | \| \| |  |  |  |  |  |  |  |  |  |  |
| 1 | transcript:Vitvi08g04305\_t001 |  | transcript:Vitvi06g04445\_t001 |  |  |  |  |  |  |  |  |  |  |
| 1 | transcript:Vitvi08g01432\_t001 |  | \| \| |  |  |  |  |  |  |  |  |  |  |
| 1 | transcript:Vitvi08g02261\_t001 |  | \| \| |  |  |  |  |  |  |  |  |  |  |
| 1 | transcript:Vitvi08g01433\_t001 |  | \| \| |  |  |  |  |  |  |  |  |  |  |
| 1 | transcript:Vitvi08g01434\_t001 |  | \| \| |  |  |  |  |  |  |  |  |  |  |
| 1 | transcript:Vitvi08g02262\_t001 |  | \| \| |  |  |  |  |  |  |  |  |  |  |
| 1 | transcript:Vitvi08g01436\_t001 |  | \| \| |  |  |  |  |  |  |  |  |  |  |
| 1 | transcript:Vitvi08g01437\_t001 |  | \| \| |  |  |  |  |  |  |  |  |  |  |
| 1 | transcript:Vitvi08g01438\_t001 |  | \| \| |  |  |  |  |  |  |  |  |  |  |
| 1 | transcript:Vitvi08g01439\_t001 |  | \| \| |  |  |  |  |  |  |  |  |  |  |
| 1 | transcript:Vitvi08g01441\_t001 |  | \| \| |  |  |  |  |  |  |  |  |  |  |
| 1 | transcript:Vitvi08g01442\_t001 |  | transcript:Vitvi06g01504\_t001 |  |  |  |  |  |  |  |  |  |  |
| 1 | transcript:Vitvi08g01443\_t001 |  | \| \| |  |  |  |  |  |  |  |  |  |  |
| 1 | transcript:Vitvi08g01444\_t001 |  | \| \| |  |  |  |  |  |  |  |  |  |  |
| 1 | transcript:Vitvi08g01445\_t001 |  | \| \| |  |  |  |  |  |  |  |  |  |  |
| 1 | transcript:Vitvi08g01448\_t001 |  | \| \| |  |  |  |  |  |  |  |  |  |  |
| 1 | transcript:Vitvi08g04306\_t001 |  | \| \| |  |  |  |  |  |  |  |  |  |  |
| 1 | transcript:Vitvi08g01450\_t002 |  | transcript:Vitvi06g01493\_t003 |  |  |  |  |  |  |  |  |  |  |
| 1 | transcript:Vitvi08g04307\_t001 |  | \| \| |  |  |  |  |  |  |  |  |  |  |
| 1 | transcript:Vitvi08g01451\_t001 |  | \| \| |  |  |  |  |  |  |  |  |  |  |
| 1 | transcript:Vitvi08g01452\_t001 |  | \| \| |  |  |  |  |  |  |  |  |  |  |
| 1 | transcript:Vitvi08g01454\_t001 |  | \| \| |  |  |  |  |  |  |  |  |  |  |
| 1 | transcript:Vitvi08g01455\_t001 |  | \| \| |  |  |  |  |  |  |  |  |  |  |
| 1 | transcript:Vitvi08g02264\_t001 |  | \| \| |  |  |  |  |  |  |  |  |  |  |
| 1 | transcript:Vitvi08g01456\_t001 |  | \| \| |  |  |  |  |  |  |  |  |  |  |
| 1 | transcript:Vitvi08g01457\_t001 |  | \| \| |  |  |  |  |  |  |  |  |  |  |
| 1 | transcript:Vitvi08g01458\_t001 |  | \| \| |  |  |  |  |  |  |  |  |  |  |
| 1 | transcript:Vitvi08g01459\_t001 |  | \| \| |  |  |  |  |  |  |  |  |  |  |
| 1 | transcript:Vitvi08g02265\_t001 |  | \| \| |  |  |  |  |  |  |  |  |  |  |
| 1 | transcript:Vitvi08g01460\_t002 |  | \| \| |  |  |  |  |  |  |  |  |  |  |
| 1 | transcript:Vitvi08g01461\_t001 |  | \| \| |  |  |  |  |  |  |  |  |  |  |
| 1 | transcript:Vitvi08g01462\_t001 |  | transcript:Vitvi06g01486\_t002 |  |  |  |  |  |  |  |  |  |  |
| 1 | transcript:Vitvi08g01463\_t001 |  | \| \| |  |  |  |  |  |  |  |  |  |  |
| 1 | transcript:Vitvi08g01464\_t001 |  | \| \| |  |  |  |  |  |  |  |  |  |  |
| 1 | transcript:Vitvi08g01465\_t001 |  | transcript:Vitvi06g01959\_t001 |  |  |  |  |  |  |  |  |  |  |
| 1 | transcript:Vitvi08g01466\_t001 |  | \| \| |  |  |  |  |  |  |  |  |  |  |
| 1 | transcript:Vitvi08g01467\_t001 |  | \| \| |  |  |  |  |  |  |  |  |  |  |
| 1 | transcript:Vitvi08g01468\_t001 |  | \| \| |  |  |  |  |  |  |  |  |  |  |
| 1 | transcript:Vitvi08g04308\_t001 |  | \| \| |  |  |  |  |  |  |  |  |  |  |
| 1 | transcript:Vitvi08g04309\_t001 |  | \| \| |  |  |  |  |  |  |  |  |  |  |
| 1 | transcript:Vitvi08g01469\_t001 |  | transcript:Vitvi06g01481\_t001 |  |  |  |  |  |  |  |  |  |  |
| 1 | transcript:Vitvi08g04310\_t001 |  | \| \| |  |  |  |  |  |  |  |  |  |  |
| 1 | transcript:Vitvi08g01470\_t001 |  | transcript:Vitvi06g01478\_t001 |  |  |  |  |  |  |  |  |  |  |
| 1 | transcript:Vitvi08g01471\_t001 |  | \| \| |  |  |  |  |  |  |  |  |  |  |
| 1 | transcript:Vitvi08g01472\_t001 |  | transcript:Vitvi06g04438\_t001 |  |  |  |  |  |  |  |  |  |  |
| 1 | transcript:Vitvi08g01473\_t001 |  | transcript:Vitvi06g01473\_t001 |  |  |  |  |  |  |  |  |  |  |
| 1 | transcript:Vitvi08g04311\_t001 |  | \| \| |  |  |  |  |  |  |  |  |  |  |
| 1 | transcript:Vitvi08g01474\_t001 |  | \| \| |  |  |  |  |  |  |  |  |  |  |
| 1 | transcript:Vitvi08g01475\_t001 |  | \| \| |  |  |  |  |  |  |  |  |  |  |
| 1 | transcript:Vitvi08g02266\_t001 |  | \| \| |  |  |  |  |  |  |  |  |  |  |
| 1 | transcript:Vitvi08g04312\_t001 |  | \| \| |  |  |  |  |  |  |  |  |  |  |
| 1 | transcript:Vitvi08g02267\_t001 |  | \| \| |  |  |  |  |  |  |  |  |  |  |
| 1 | transcript:Vitvi08g02268\_t001 |  | \| \| |  |  |  |  |  |  |  |  |  |  |
| 1 | transcript:Vitvi08g04313\_t001 |  | \| \| |  |  |  |  |  |  |  |  |  |  |
| 1 | transcript:Vitvi08g01476\_t001 |  | \| \| |  |  |  |  |  |  |  |  |  |  |
| 1 | transcript:Vitvi08g02270\_t001 |  | \| \| |  |  |  |  |  |  |  |  |  |  |
| 1 | transcript:Vitvi08g04314\_t001 |  | \| \| |  |  |  |  |  |  |  |  |  |  |
| 1 | transcript:Vitvi08g01478\_t001 |  | \| \| |  |  |  |  |  |  |  |  |  |  |
| 1 | transcript:Vitvi08g01480\_t001 |  | \| \| |  |  |  |  |  |  |  |  |  |  |
| 1 | transcript:Vitvi08g04315\_t001 |  | \| \| |  |  |  |  |  |  |  |  |  |  |
| 1 | transcript:Vitvi08g01481\_t001 |  | \| \| |  |  |  |  |  |  |  |  |  |  |
| 1 | transcript:Vitvi08g01482\_t001 |  | \| \| |  |  |  |  |  |  |  |  |  |  |
| 1 | transcript:Vitvi08g01483\_t001 |  | \| \| |  |  |  |  |  |  |  |  |  |  |
| 1 | transcript:Vitvi08g02271\_t001 |  | \| \| |  |  |  |  |  |  |  |  |  |  |
| 1 | transcript:Vitvi08g01484\_t001 |  | \| \| |  |  |  |  |  |  |  |  |  |  |
| 1 | transcript:Vitvi08g02272\_t004 |  | \| \| |  |  |  |  |  |  |  |  |  |  |
| 1 | transcript:Vitvi08g01485\_t001 |  | transcript:Vitvi06g01452\_t001 |  |  |  |  |  |  |  |  |  |  |
| 1 | transcript:Vitvi08g04316\_t001 |  | \| \| |  |  |  |  |  |  |  |  |  |  |
| 1 | transcript:Vitvi08g01487\_t001 |  | \| \| |  |  |  |  |  |  |  |  |  |  |
| 1 | transcript:Vitvi08g04317\_t001 |  | \| \| |  |  |  |  |  |  |  |  |  |  |
| 1 | transcript:Vitvi08g01488\_t001 |  | \| \| |  |  |  |  |  |  |  |  |  |  |
| 1 | transcript:Vitvi08g01489\_t001 |  | \| \| |  |  |  |  |  |  |  |  |  |  |
| 1 | transcript:Vitvi08g01490\_t001 |  | \| \| |  |  |  |  |  |  |  |  |  |  |
| 1 | transcript:Vitvi08g01492\_t001 |  | \| \| |  |  |  |  |  |  |  |  |  |  |
| 1 | transcript:Vitvi08g01493\_t001 |  | transcript:Vitvi06g01447\_t001 |  |  |  |  |  |  |  |  |  |  |
| 1 | transcript:Vitvi08g01494\_t001 |  | \| \| |  |  |  |  |  |  |  |  |  |  |
| 1 | transcript:Vitvi08g04318\_t001 |  | \| \| |  |  |  |  |  |  |  |  |  |  |
| 1 | transcript:Vitvi08g01497\_t001 |  | \| \| |  |  |  |  |  |  |  |  |  |  |
| 1 | transcript:Vitvi08g01498\_t001 |  | \| \| |  |  |  |  |  |  |  |  |  |  |
| 1 | transcript:Vitvi08g01499\_t001 |  | transcript:Vitvi06g01418\_t001 |  |  |  |  |  |  |  |  |  |  |
| 1 | transcript:Vitvi08g04319\_t001 |  | \| \| |  |  |  |  |  |  |  |  |  |  |
| 1 | transcript:Vitvi08g01500\_t001 |  | \| \| |  |  |  |  |  |  |  |  |  |  |
| 1 | transcript:Vitvi08g01501\_t001 |  | transcript:Vitvi06g01411\_t001 |  |  |  |  |  |  |  |  |  |  |
| 1 | transcript:Vitvi08g01502\_t001 |  | \| \| |  |  |  |  |  |  |  |  |  |  |
| 1 | transcript:Vitvi08g04320\_t001 |  | \| \| |  |  |  |  |  |  |  |  |  |  |
| 1 | transcript:Vitvi08g01505\_t001 |  | \| \| |  |  |  |  |  |  |  |  |  |  |
| 1 | transcript:Vitvi08g01506\_t001 |  | \| \| |  |  |  |  |  |  |  |  |  |  |
| 1 | transcript:Vitvi08g04321\_t001 |  | \| \| |  |  |  |  |  |  |  |  |  |  |
| 1 | transcript:Vitvi08g01507\_t001 |  | transcript:Vitvi06g01408\_t001 |  |  |  |  |  |  |  |  |  |  |
| 1 | transcript:Vitvi08g04322\_t001 |  | \| \| |  |  |  |  |  |  |  |  |  |  |
| 1 | transcript:Vitvi08g04323\_t001 |  | \| \| |  |  |  |  |  |  |  |  |  |  |
| 1 | transcript:Vitvi08g01508\_t002 |  | transcript:Vitvi06g01406\_t001 |  |  |  |  |  |  |  |  |  |  |
| 1 | transcript:Vitvi08g01509\_t001 |  | \| \| |  |  |  |  |  |  |  |  |  |  |
| 1 | transcript:Vitvi08g01510\_t001 |  | transcript:Vitvi06g01945\_t001 |  |  |  |  |  |  |  |  |  |  |
| 1 | transcript:Vitvi08g01511\_t001 |  | \| \| |  |  |  |  |  |  |  |  |  |  |
| 1 | transcript:Vitvi08g04324\_t001 |  | \| \| |  |  |  |  |  |  |  |  |  |  |
| 1 | transcript:Vitvi08g01512\_t001 |  | \| \| |  |  |  |  |  |  |  |  |  |  |
| 1 | transcript:Vitvi08g01513\_t001 |  | \| \| |  |  |  |  |  |  |  |  |  |  |
| 1 | transcript:Vitvi08g04325\_t001 |  | \| \| |  |  |  |  |  |  |  |  |  |  |
| 1 | transcript:Vitvi08g01514\_t001 |  | \| \| |  |  |  |  |  |  |  |  |  |  |
| 1 | transcript:Vitvi08g01515\_t001 |  | transcript:Vitvi06g01402\_t001 |  |  |  |  |  |  |  |  |  |  |
| 1 | transcript:Vitvi08g04326\_t001 |  | \| \| |  |  |  |  |  |  |  |  |  |  |
| 1 | transcript:Vitvi08g04327\_t001 |  | \| \| |  |  |  |  |  |  |  |  |  |  |
| 1 | transcript:Vitvi08g01517\_t001 |  | \| \| |  |  |  |  |  |  |  |  |  |  |
| 1 | transcript:Vitvi08g01518\_t002 |  | transcript:Vitvi06g01399\_t001 |  |  |  |  |  |  |  |  |  |  |
| 1 | transcript:Vitvi08g01519\_t003 |  | \| \| |  |  |  |  |  |  |  |  |  |  |
| 1 | transcript:Vitvi08g01520\_t002 |  | \| \| |  |  |  |  |  |  |  |  |  |  |
| 1 | transcript:Vitvi08g01521\_t001 |  | \| \| |  |  |  |  |  |  |  |  |  |  |
| 1 | transcript:Vitvi08g01522\_t001 |  | \| \| |  |  |  |  |  |  |  |  |  |  |
| 1 | transcript:Vitvi08g01523\_t002 |  | transcript:Vitvi06g01398\_t001 |  |  |  |  |  |  |  |  |  |  |
| 1 | transcript:Vitvi08g02274\_t001 |  | \| \| |  |  |  |  |  |  |  |  |  |  |
| 1 | transcript:Vitvi08g01524\_t001 |  | \| \| |  |  |  |  |  |  |  |  |  |  |
| 1 | transcript:Vitvi08g01525\_t001 |  | transcript:Vitvi06g01944\_t001 |  |  |  |  |  |  |  |  |  |  |
| 0 | transcript:Vitvi08g04328\_t001 |  |  |  |  |  |  |  |  |  |  |  |  |
| 0 | transcript:Vitvi08g01527\_t001 |  |  |  |  |  |  |  |  |  |  |  |  |
| 0 | transcript:Vitvi08g01528\_t001 |  |  |  |  |  |  |  |  |  |  |  |  |
| 0 | transcript:Vitvi08g02275\_t001 |  |  |  |  |  |  |  |  |  |  |  |  |
| 0 | transcript:Vitvi08g04329\_t001 |  |  |  |  |  |  |  |  |  |  |  |  |
| 0 | transcript:Vitvi08g01529\_t001 |  |  |  |  |  |  |  |  |  |  |  |  |
| 0 | transcript:Vitvi08g02277\_t001 |  |  |  |  |  |  |  |  |  |  |  |  |
| 0 | transcript:Vitvi08g04330\_t001 |  |  |  |  |  |  |  |  |  |  |  |  |
| 0 | transcript:Vitvi08g01531\_t001 |  |  |  |  |  |  |  |  |  |  |  |  |
| 0 | transcript:Vitvi08g04331\_t001 |  |  |  |  |  |  |  |  |  |  |  |  |
| 0 | transcript:Vitvi08g04332\_t001 |  |  |  |  |  |  |  |  |  |  |  |  |
| 0 | transcript:Vitvi08g04333\_t001 |  |  |  |  |  |  |  |  |  |  |  |  |
| 0 | transcript:Vitvi08g01534\_t001 |  |  |  |  |  |  |  |  |  |  |  |  |
| 0 | transcript:Vitvi08g04334\_t001 |  |  |  |  |  |  |  |  |  |  |  |  |
| 0 | transcript:Vitvi08g02280\_t001 |  |  |  |  |  |  |  |  |  |  |  |  |
| 0 | transcript:Vitvi08g01535\_t001 |  |  |  |  |  |  |  |  |  |  |  |  |
| 0 | transcript:Vitvi08g01536\_t001 |  |  |  |  |  |  |  |  |  |  |  |  |
| 0 | transcript:Vitvi08g02281\_t001 |  |  |  |  |  |  |  |  |  |  |  |  |
| 0 | transcript:Vitvi08g01537\_t001 |  |  |  |  |  |  |  |  |  |  |  |  |
| 0 | transcript:Vitvi08g01538\_t002 |  |  |  |  |  |  |  |  |  |  |  |  |
| 0 | transcript:Vitvi08g01539\_t001 |  |  |  |  |  |  |  |  |  |  |  |  |
| 0 | transcript:Vitvi08g01540\_t001 |  |  |  |  |  |  |  |  |  |  |  |  |
| 0 | transcript:Vitvi08g04335\_t002 |  |  |  |  |  |  |  |  |  |  |  |  |
| 0 | transcript:Vitvi08g01542\_t001 |  |  |  |  |  |  |  |  |  |  |  |  |
| 0 | transcript:Vitvi08g04336\_t001 |  |  |  |  |  |  |  |  |  |  |  |  |
| 0 | transcript:Vitvi08g01543\_t001 |  |  |  |  |  |  |  |  |  |  |  |  |
| 1 | transcript:Vitvi08g01544\_t001 |  | transcript:Vitvi06g01406\_t001 |  |  |  |  |  |  |  |  |  |  |
| 1 | transcript:Vitvi08g01545\_t001 |  | \| \| |  |  |  |  |  |  |  |  |  |  |
| 1 | transcript:Vitvi08g01546\_t001 |  | \| \| |  |  |  |  |  |  |  |  |  |  |
| 1 | transcript:Vitvi08g04337\_t001 |  | \| \| |  |  |  |  |  |  |  |  |  |  |
| 1 | transcript:Vitvi08g01547\_t001 |  | \| \| |  |  |  |  |  |  |  |  |  |  |
| 1 | transcript:Vitvi08g01548\_t001 |  | \| \| |  |  |  |  |  |  |  |  |  |  |
| 1 | transcript:Vitvi08g01549\_t001 |  | \| \| |  |  |  |  |  |  |  |  |  |  |
| 1 | transcript:Vitvi08g01550\_t001 |  | \| \| |  |  |  |  |  |  |  |  |  |  |
| 1 | transcript:Vitvi08g01551\_t001.1.6037826f |  | \| \| |  |  |  |  |  |  |  |  |  |  |
| 1 | transcript:Vitvi08g01553\_t001 |  | transcript:Vitvi06g01387\_t001 |  |  |  |  |  |  |  |  |  |  |
| 1 | transcript:Vitvi08g01554\_t001 |  | \| \| |  |  |  |  |  |  |  |  |  |  |
| 1 | transcript:Vitvi08g02282\_t001 |  | \| \| |  |  |  |  |  |  |  |  |  |  |
| 1 | transcript:Vitvi08g01555\_t001 |  | \| \| |  |  |  |  |  |  |  |  |  |  |
| 1 | transcript:Vitvi08g01556\_t001 |  | \| \| |  |  |  |  |  |  |  |  |  |  |
| 1 | transcript:Vitvi08g01558\_t001 |  | \| \| |  |  |  |  |  |  |  |  |  |  |
| 1 | transcript:Vitvi08g01559\_t001 |  | transcript:Vitvi06g01378\_t001 |  |  |  |  |  |  |  |  |  |  |
| 1 | transcript:Vitvi08g01560\_t001 |  | \| \| |  |  |  |  |  |  |  |  |  |  |
| 1 | transcript:Vitvi08g01561\_t001 |  | \| \| |  |  |  |  |  |  |  |  |  |  |
| 1 | transcript:Vitvi08g01562\_t001 |  | transcript:Vitvi06g01377\_t001 |  |  |  |  |  |  |  |  |  |  |
| 1 | transcript:Vitvi08g04338\_t001 |  | \| \| |  |  |  |  |  |  |  |  |  |  |
| 1 | transcript:Vitvi08g04339\_t001 |  | \| \| |  |  |  |  |  |  |  |  |  |  |
| 1 | transcript:Vitvi08g02283\_t002 |  | \| \| |  |  |  |  |  |  |  |  |  |  |
| 1 | transcript:Vitvi08g02286\_t001 |  | \| \| |  |  |  |  |  |  |  |  |  |  |
| 1 | transcript:Vitvi08g04340\_t001 |  | \| \| |  |  |  |  |  |  |  |  |  |  |
| 1 | transcript:Vitvi08g01567\_t001 |  | \| \| |  |  |  |  |  |  |  |  |  |  |
| 1 | transcript:Vitvi08g01568\_t001 |  | \| \| |  |  |  |  |  |  |  |  |  |  |
| 1 | transcript:Vitvi08g01569\_t001 |  | transcript:Vitvi06g01362\_t001 |  |  |  |  |  |  |  |  |  |  |
| 1 | transcript:Vitvi08g01570\_t001 |  | \| \| |  |  |  |  |  |  |  |  |  |  |
| 1 | transcript:Vitvi08g01571\_t001 |  | \| \| |  |  |  |  |  |  |  |  |  |  |
| 1 | transcript:Vitvi08g01572\_t001 |  | \| \| |  |  |  |  |  |  |  |  |  |  |
| 1 | transcript:Vitvi08g01573\_t001 |  | \| \| |  |  |  |  |  |  |  |  |  |  |
| 1 | transcript:Vitvi08g01574\_t001 |  | \| \| |  |  |  |  |  |  |  |  |  |  |
| 1 | transcript:Vitvi08g04341\_t001 |  | \| \| |  |  |  |  |  |  |  |  |  |  |
| 1 | transcript:Vitvi08g01575\_t001 |  | \| \| |  |  |  |  |  |  |  |  |  |  |
| 1 | transcript:Vitvi08g01577\_t001 |  | \| \| |  |  |  |  |  |  |  |  |  |  |
| 1 | transcript:Vitvi08g01578\_t001 |  | \| \| |  |  |  |  |  |  |  |  |  |  |
| 1 | transcript:Vitvi08g01579\_t002 |  | transcript:Vitvi06g01361\_t001 |  |  |  |  |  |  |  |  |  |  |
| 1 | transcript:Vitvi08g01580\_t001 |  | \| \| |  |  |  |  |  |  |  |  |  |  |
| 1 | transcript:Vitvi08g02288\_t001 |  | \| \| |  |  |  |  |  |  |  |  |  |  |
| 1 | transcript:Vitvi08g04342\_t001 |  | \| \| |  |  |  |  |  |  |  |  |  |  |
| 1 | transcript:Vitvi08g01581\_t001 |  | \| \| |  |  |  |  |  |  |  |  |  |  |
| 1 | transcript:Vitvi08g02289\_t001 |  | \| \| |  |  |  |  |  |  |  |  |  |  |
| 1 | transcript:Vitvi08g01582\_t001 |  | \| \| |  |  |  |  |  |  |  |  |  |  |
| 1 | transcript:Vitvi08g01583\_t001 |  | \| \| |  |  |  |  |  |  |  |  |  |  |
| 1 | transcript:Vitvi08g02291\_t001 |  | \| \| |  |  |  |  |  |  |  |  |  |  |
| 1 | transcript:Vitvi08g04343\_t001 |  | \| \| |  |  |  |  |  |  |  |  |  |  |
| 1 | transcript:Vitvi08g01584\_t001 |  | \| \| |  |  |  |  |  |  |  |  |  |  |
| 1 | transcript:Vitvi08g01585\_t001 |  | \| \| |  |  |  |  |  |  |  |  |  |  |
| 1 | transcript:Vitvi08g02292\_t001 |  | \| \| |  |  |  |  |  |  |  |  |  |  |
| 1 | transcript:Vitvi08g01586\_t001 |  | \| \| |  |  |  |  |  |  |  |  |  |  |
| 1 | transcript:Vitvi08g01587\_t001 |  | \| \| |  |  |  |  |  |  |  |  |  |  |
| 1 | transcript:Vitvi08g01588\_t001 |  | \| \| |  |  |  |  |  |  |  |  |  |  |
| 1 | transcript:Vitvi08g04344\_t001 |  | \| \| |  |  |  |  |  |  |  |  |  |  |
| 1 | transcript:Vitvi08g01589\_t001 |  | \| \| |  |  |  |  |  |  |  |  |  |  |
| 1 | transcript:Vitvi08g01591\_t001 |  | \| \| |  |  |  |  |  |  |  |  |  |  |
| 1 | transcript:Vitvi08g04345\_t001 |  | \| \| |  |  |  |  |  |  |  |  |  |  |
| 1 | transcript:Vitvi08g01596\_t001 |  | \| \| |  |  |  |  |  |  |  |  |  |  |
| 1 | transcript:Vitvi08g01597\_t001 |  | \| \| |  |  |  |  |  |  |  |  |  |  |
| 1 | transcript:Vitvi08g01599\_t001 |  | \| \| |  |  |  |  |  |  |  |  |  |  |
| 1 | transcript:Vitvi08g01600\_t001 |  | transcript:Vitvi06g01350\_t001 |  |  |  |  |  |  |  |  |  |  |
| 1 | transcript:Vitvi08g01602\_t001 |  | transcript:Vitvi06g01346\_t001 |  |  |  |  |  |  |  |  |  |  |
| 1 | transcript:Vitvi08g01603\_t004 |  | transcript:Vitvi06g01344\_t001 |  |  |  |  |  |  |  |  |  |  |
| 1 | transcript:Vitvi08g01604\_t001 |  | \| \| |  |  |  |  |  |  |  |  |  |  |
| 1 | transcript:Vitvi08g02295\_t001 |  | \| \| |  |  |  |  |  |  |  |  |  |  |
| 2 | transcript:Vitvi08g01606\_t001 |  | \| \| |  | transcript:Vitvi13g00667\_t001 |  |  |  |  |  |  |  |  |
| 2 | transcript:Vitvi08g01607\_t001 |  | \| \| |  | \| \| |  |  |  |  |  |  |  |  |
| 2 | transcript:Vitvi08g01608\_t001 |  | \| \| |  | \| \| |  |  |  |  |  |  |  |  |
| 2 | transcript:Vitvi08g01609\_t001 |  | \| \| |  | transcript:Vitvi13g00670\_t001 |  |  |  |  |  |  |  |  |
| 2 | transcript:Vitvi08g01611\_t001 |  | transcript:Vitvi06g01334\_t001 |  | transcript:Vitvi13g00675\_t001 |  |  |  |  |  |  |  |  |
| 2 | transcript:Vitvi08g01612\_t001 |  | \| \| |  | transcript:Vitvi13g00684\_t001 |  |  |  |  |  |  |  |  |
| 2 | transcript:Vitvi08g01613\_t001 |  | \| \| |  | \| \| |  |  |  |  |  |  |  |  |
| 2 | transcript:Vitvi08g01614\_t001 |  | \| \| |  | transcript:Vitvi13g00686\_t001 |  |  |  |  |  |  |  |  |
| 2 | transcript:Vitvi08g02296\_t002 |  | \| \| |  | \| \| |  |  |  |  |  |  |  |  |
| 2 | transcript:Vitvi08g01615\_t001 |  | \| \| |  | \| \| |  |  |  |  |  |  |  |  |
| 2 | transcript:Vitvi08g01616\_t001 |  | \| \| |  | \| \| |  |  |  |  |  |  |  |  |
| 2 | transcript:Vitvi08g04346\_t001 |  | \| \| |  | \| \| |  |  |  |  |  |  |  |  |
| 2 | transcript:Vitvi08g04347\_t001 |  | \| \| |  | \| \| |  |  |  |  |  |  |  |  |
| 2 | transcript:Vitvi08g01617\_t001 |  | transcript:Vitvi06g01329\_t001 |  | \| \| |  |  |  |  |  |  |  |  |
| 2 | transcript:Vitvi08g04348\_t001 |  | \| \| |  | transcript:Vitvi13g04196\_t001 |  |  |  |  |  |  |  |  |
| 1 | transcript:Vitvi08g02299\_t001 |  | \| \| |  |  |  |  |  |  |  |  |  |  |
| 1 | transcript:Vitvi08g01618\_t001 |  | \| \| |  |  |  |  |  |  |  |  |  |  |
| 1 | transcript:Vitvi08g01619\_t001 |  | \| \| |  |  |  |  |  |  |  |  |  |  |
| 1 | transcript:Vitvi08g04349\_t001 |  | transcript:Vitvi06g01325\_t001 |  |  |  |  |  |  |  |  |  |  |
| 1 | transcript:Vitvi08g01621\_t001 |  | \| \| |  |  |  |  |  |  |  |  |  |  |
| 1 | transcript:Vitvi08g01622\_t001 |  | \| \| |  |  |  |  |  |  |  |  |  |  |
| 1 | transcript:Vitvi08g01623\_t001 |  | transcript:Vitvi06g01321\_t001 |  |  |  |  |  |  |  |  |  |  |
| 1 | transcript:Vitvi08g01624\_t001 |  | \| \| |  |  |  |  |  |  |  |  |  |  |
| 1 | transcript:Vitvi08g04350\_t001 |  | \| \| |  |  |  |  |  |  |  |  |  |  |
| 1 | transcript:Vitvi08g04351\_t001 |  | transcript:Vitvi06g01318\_t001 |  |  |  |  |  |  |  |  |  |  |
| 0 | transcript:Vitvi08g01627\_t001 |  |  |  |  |  |  |  |  |  |  |  |  |
| 0 | transcript:Vitvi08g04352\_t001 |  |  |  |  |  |  |  |  |  |  |  |  |
| 0 | transcript:Vitvi08g01629\_t002 |  |  |  |  |  |  |  |  |  |  |  |  |
| 0 | transcript:Vitvi08g01630\_t001 |  |  |  |  |  |  |  |  |  |  |  |  |
| 0 | transcript:Vitvi08g01631\_t001 |  |  |  |  |  |  |  |  |  |  |  |  |
| 0 | transcript:Vitvi08g02301\_t001 |  |  |  |  |  |  |  |  |  |  |  |  |
| 0 | transcript:Vitvi08g01634\_t001 |  |  |  |  |  |  |  |  |  |  |  |  |
| 0 | transcript:Vitvi08g01635\_t001 |  |  |  |  |  |  |  |  |  |  |  |  |
| 0 | transcript:Vitvi08g04353\_t001 |  |  |  |  |  |  |  |  |  |  |  |  |
| 0 | transcript:Vitvi08g04354\_t001 |  |  |  |  |  |  |  |  |  |  |  |  |
| 0 | transcript:Vitvi08g04355\_t001 |  |  |  |  |  |  |  |  |  |  |  |  |
| 0 | transcript:Vitvi08g01638\_t001 |  |  |  |  |  |  |  |  |  |  |  |  |
| 0 | transcript:Vitvi08g01639\_t001 |  |  |  |  |  |  |  |  |  |  |  |  |
| 0 | transcript:Vitvi08g01640\_t001 |  |  |  |  |  |  |  |  |  |  |  |  |
| 0 | transcript:Vitvi08g01641\_t001 |  |  |  |  |  |  |  |  |  |  |  |  |
| 0 | transcript:Vitvi08g02304\_t001 |  |  |  |  |  |  |  |  |  |  |  |  |
| 0 | transcript:Vitvi08g01642\_t001 |  |  |  |  |  |  |  |  |  |  |  |  |
| 0 | transcript:Vitvi08g01644\_t001 |  |  |  |  |  |  |  |  |  |  |  |  |
| 0 | transcript:Vitvi08g04356\_t001 |  |  |  |  |  |  |  |  |  |  |  |  |
| 0 | transcript:Vitvi08g01645\_t001 |  |  |  |  |  |  |  |  |  |  |  |  |
| 0 | transcript:Vitvi08g01646\_t001 |  |  |  |  |  |  |  |  |  |  |  |  |
| 0 | transcript:Vitvi08g04357\_t001 |  |  |  |  |  |  |  |  |  |  |  |  |
| 0 | transcript:Vitvi08g04358\_t001 |  |  |  |  |  |  |  |  |  |  |  |  |
| 0 | transcript:Vitvi08g01649\_t001 |  |  |  |  |  |  |  |  |  |  |  |  |
| 0 | transcript:Vitvi08g01651\_t001 |  |  |  |  |  |  |  |  |  |  |  |  |
| 0 | transcript:Vitvi08g01652\_t001 |  |  |  |  |  |  |  |  |  |  |  |  |
| 0 | transcript:Vitvi08g01653\_t001 |  |  |  |  |  |  |  |  |  |  |  |  |
| 0 | transcript:Vitvi08g01655\_t002 |  |  |  |  |  |  |  |  |  |  |  |  |
| 0 | transcript:Vitvi08g01656\_t001 |  |  |  |  |  |  |  |  |  |  |  |  |
| 0 | transcript:Vitvi08g01657\_t001 |  |  |  |  |  |  |  |  |  |  |  |  |
| 0 | transcript:Vitvi08g02307\_t001 |  |  |  |  |  |  |  |  |  |  |  |  |
| 0 | transcript:Vitvi08g04359\_t001 |  |  |  |  |  |  |  |  |  |  |  |  |
| 0 | transcript:Vitvi08g01659\_t001 |  |  |  |  |  |  |  |  |  |  |  |  |
| 0 | transcript:Vitvi08g01660\_t001 |  |  |  |  |  |  |  |  |  |  |  |  |
| 0 | transcript:Vitvi08g01661\_t002 |  |  |  |  |  |  |  |  |  |  |  |  |
| 0 | transcript:Vitvi08g01662\_t001 |  |  |  |  |  |  |  |  |  |  |  |  |
| 0 | transcript:Vitvi08g02308\_t001 |  |  |  |  |  |  |  |  |  |  |  |  |
| 0 | transcript:Vitvi08g02309\_t001 |  |  |  |  |  |  |  |  |  |  |  |  |
| 0 | transcript:Vitvi08g04360\_t001 |  |  |  |  |  |  |  |  |  |  |  |  |
| 0 | transcript:Vitvi08g04361\_t001 |  |  |  |  |  |  |  |  |  |  |  |  |
| 0 | transcript:Vitvi08g04362\_t001 |  |  |  |  |  |  |  |  |  |  |  |  |
| 0 | transcript:Vitvi08g01664\_t001 |  |  |  |  |  |  |  |  |  |  |  |  |
| 0 | transcript:Vitvi08g04363\_t001 |  |  |  |  |  |  |  |  |  |  |  |  |
| 0 | transcript:Vitvi08g01666\_t001 |  |  |  |  |  |  |  |  |  |  |  |  |
| 0 | transcript:Vitvi08g04364\_t001 |  |  |  |  |  |  |  |  |  |  |  |  |
| 0 | transcript:Vitvi08g01667\_t001 |  |  |  |  |  |  |  |  |  |  |  |  |
| 0 | transcript:Vitvi08g01668\_t001 |  |  |  |  |  |  |  |  |  |  |  |  |
| 0 | transcript:Vitvi08g02313\_t001 |  |  |  |  |  |  |  |  |  |  |  |  |
| 0 | transcript:Vitvi08g02314\_t001 |  |  |  |  |  |  |  |  |  |  |  |  |
| 0 | transcript:Vitvi08g02315\_t001 |  |  |  |  |  |  |  |  |  |  |  |  |
| 0 | transcript:Vitvi08g01669\_t001 |  |  |  |  |  |  |  |  |  |  |  |  |
| 0 | transcript:Vitvi08g02317\_t001 |  |  |  |  |  |  |  |  |  |  |  |  |
| 0 | transcript:Vitvi08g01670\_t001 |  |  |  |  |  |  |  |  |  |  |  |  |
| 0 | transcript:Vitvi08g01671\_t001 |  |  |  |  |  |  |  |  |  |  |  |  |
| 0 | transcript:Vitvi08g04365\_t001 |  |  |  |  |  |  |  |  |  |  |  |  |
| 0 | transcript:Vitvi08g01672\_t001 |  |  |  |  |  |  |  |  |  |  |  |  |
| 0 | transcript:Vitvi08g01673\_t001 |  |  |  |  |  |  |  |  |  |  |  |  |
| 0 | transcript:Vitvi08g04366\_t001 |  |  |  |  |  |  |  |  |  |  |  |  |
| 0 | transcript:Vitvi08g04367\_t001 |  |  |  |  |  |  |  |  |  |  |  |  |
| 0 | transcript:Vitvi08g04368\_t001 |  |  |  |  |  |  |  |  |  |  |  |  |
| 0 | transcript:Vitvi08g04369\_t001 |  |  |  |  |  |  |  |  |  |  |  |  |
| 0 | transcript:Vitvi08g02318\_t001 |  |  |  |  |  |  |  |  |  |  |  |  |
| 0 | transcript:Vitvi08g04370\_t001 |  |  |  |  |  |  |  |  |  |  |  |  |
| 0 | transcript:Vitvi08g04371\_t001 |  |  |  |  |  |  |  |  |  |  |  |  |
| 0 | transcript:Vitvi08g02320\_t003 |  |  |  |  |  |  |  |  |  |  |  |  |
| 0 | transcript:Vitvi08g04372\_t001 |  |  |  |  |  |  |  |  |  |  |  |  |
| 0 | transcript:Vitvi08g04373\_t001 |  |  |  |  |  |  |  |  |  |  |  |  |
| 0 | transcript:Vitvi08g04374\_t001 |  |  |  |  |  |  |  |  |  |  |  |  |
| 0 | transcript:Vitvi08g04375\_t001 |  |  |  |  |  |  |  |  |  |  |  |  |
| 0 | transcript:Vitvi08g02323\_t001 |  |  |  |  |  |  |  |  |  |  |  |  |
| 0 | transcript:Vitvi08g04376\_t001 |  |  |  |  |  |  |  |  |  |  |  |  |
| 0 | transcript:Vitvi08g04377\_t001 |  |  |  |  |  |  |  |  |  |  |  |  |
| 0 | transcript:Vitvi08g02324\_t001 |  |  |  |  |  |  |  |  |  |  |  |  |
| 0 | transcript:Vitvi08g04378\_t001 |  |  |  |  |  |  |  |  |  |  |  |  |
| 0 | transcript:Vitvi08g02326\_t001 |  |  |  |  |  |  |  |  |  |  |  |  |
| 0 | transcript:Vitvi08g01674\_t001 |  |  |  |  |  |  |  |  |  |  |  |  |
| 0 | transcript:Vitvi08g04379\_t001 |  |  |  |  |  |  |  |  |  |  |  |  |
| 0 | transcript:Vitvi08g01675\_t002 |  |  |  |  |  |  |  |  |  |  |  |  |
| 0 | transcript:Vitvi08g01676\_t001 |  |  |  |  |  |  |  |  |  |  |  |  |
| 0 | transcript:Vitvi08g04380\_t001 |  |  |  |  |  |  |  |  |  |  |  |  |
| 0 | transcript:Vitvi08g01677\_t001 |  |  |  |  |  |  |  |  |  |  |  |  |
| 0 | transcript:Vitvi08g01678\_t001 |  |  |  |  |  |  |  |  |  |  |  |  |
| 0 | transcript:Vitvi08g01679\_t001 |  |  |  |  |  |  |  |  |  |  |  |  |
| 0 | transcript:Vitvi08g01680\_t001 |  |  |  |  |  |  |  |  |  |  |  |  |
| 0 | transcript:Vitvi08g01681\_t001 |  |  |  |  |  |  |  |  |  |  |  |  |
| 0 | transcript:Vitvi08g01682\_t001 |  |  |  |  |  |  |  |  |  |  |  |  |
| 0 | transcript:Vitvi08g01683\_t001 |  |  |  |  |  |  |  |  |  |  |  |  |
| 0 | transcript:Vitvi08g01684\_t001 |  |  |  |  |  |  |  |  |  |  |  |  |
| 0 | transcript:Vitvi08g01685\_t001 |  |  |  |  |  |  |  |  |  |  |  |  |
| 0 | transcript:Vitvi08g02328\_t001 |  |  |  |  |  |  |  |  |  |  |  |  |
| 0 | transcript:Vitvi08g04381\_t001 |  |  |  |  |  |  |  |  |  |  |  |  |
| 0 | transcript:Vitvi08g02329\_t001 |  |  |  |  |  |  |  |  |  |  |  |  |
| 0 | transcript:Vitvi08g04382\_t001 |  |  |  |  |  |  |  |  |  |  |  |  |
| 0 | transcript:Vitvi08g01686\_t001 |  |  |  |  |  |  |  |  |  |  |  |  |
| 0 | transcript:Vitvi08g01687\_t001 |  |  |  |  |  |  |  |  |  |  |  |  |
| 0 | transcript:Vitvi08g04383\_t001 |  |  |  |  |  |  |  |  |  |  |  |  |
| 0 | transcript:Vitvi08g01689\_t001 |  |  |  |  |  |  |  |  |  |  |  |  |
| 0 | transcript:Vitvi08g01690\_t001 |  |  |  |  |  |  |  |  |  |  |  |  |
| 0 | transcript:Vitvi08g01691\_t001 |  |  |  |  |  |  |  |  |  |  |  |  |
| 0 | transcript:Vitvi08g01692\_t001 |  |  |  |  |  |  |  |  |  |  |  |  |
| 0 | transcript:Vitvi08g01693\_t001 |  |  |  |  |  |  |  |  |  |  |  |  |
| 0 | transcript:Vitvi08g01694\_t001 |  |  |  |  |  |  |  |  |  |  |  |  |
| 0 | transcript:Vitvi08g01968\_t001 |  |  |  |  |  |  |  |  |  |  |  |  |
| 0 | transcript:Vitvi08g01695\_t001 |  |  |  |  |  |  |  |  |  |  |  |  |
| 0 | transcript:Vitvi08g01696\_t001 |  |  |  |  |  |  |  |  |  |  |  |  |
| 0 | transcript:Vitvi08g01697\_t001 |  |  |  |  |  |  |  |  |  |  |  |  |
| 0 | transcript:Vitvi08g01698\_t001 |  |  |  |  |  |  |  |  |  |  |  |  |
| 0 | transcript:Vitvi08g02330\_t001 |  |  |  |  |  |  |  |  |  |  |  |  |
| 0 | transcript:Vitvi08g02331\_t001 |  |  |  |  |  |  |  |  |  |  |  |  |
| 0 | transcript:Vitvi08g02332\_t002 |  |  |  |  |  |  |  |  |  |  |  |  |
| 0 | transcript:Vitvi08g01699\_t001 |  |  |  |  |  |  |  |  |  |  |  |  |
| 0 | transcript:Vitvi08g02333\_t001 |  |  |  |  |  |  |  |  |  |  |  |  |
| 0 | transcript:Vitvi08g02334\_t001 |  |  |  |  |  |  |  |  |  |  |  |  |
| 0 | transcript:Vitvi08g02335\_t001 |  |  |  |  |  |  |  |  |  |  |  |  |
| 0 | transcript:Vitvi08g02336\_t001 |  |  |  |  |  |  |  |  |  |  |  |  |
| 0 | transcript:Vitvi08g01701\_t001 |  |  |  |  |  |  |  |  |  |  |  |  |
| 0 | transcript:Vitvi08g04384\_t001 |  |  |  |  |  |  |  |  |  |  |  |  |
| 0 | transcript:Vitvi08g01702\_t001 |  |  |  |  |  |  |  |  |  |  |  |  |
| 0 | transcript:Vitvi08g04385\_t001 |  |  |  |  |  |  |  |  |  |  |  |  |
| 0 | transcript:Vitvi08g04386\_t001 |  |  |  |  |  |  |  |  |  |  |  |  |
| 0 | transcript:Vitvi08g01707\_t001 |  |  |  |  |  |  |  |  |  |  |  |  |
| 0 | transcript:Vitvi08g04387\_t001 |  |  |  |  |  |  |  |  |  |  |  |  |
| 1 | transcript:Vitvi08g01708\_t001 |  | transcript:Vitvi06g00162\_t001 |  |  |  |  |  |  |  |  |  |  |
| 1 | transcript:Vitvi08g01709\_t001 |  | \| \| |  |  |  |  |  |  |  |  |  |  |
| 1 | transcript:Vitvi08g01710\_t001 |  | \| \| |  |  |  |  |  |  |  |  |  |  |
| 1 | transcript:Vitvi08g01711\_t001 |  | \| \| |  |  |  |  |  |  |  |  |  |  |
| 1 | transcript:Vitvi08g04388\_t001 |  | \| \| |  |  |  |  |  |  |  |  |  |  |
| 2 | transcript:Vitvi08g04389\_t001 |  | \| \| |  | transcript:Vitvi13g02566\_t001 |  |  |  |  |  |  |  |  |
| 2 | transcript:Vitvi08g01713\_t001 |  | \| \| |  | \| \| |  |  |  |  |  |  |  |  |
| 2 | transcript:Vitvi08g01714\_t001 |  | \| \| |  | \| \| |  |  |  |  |  |  |  |  |
| 2 | transcript:Vitvi08g04390\_t001 |  | \| \| |  | \| \| |  |  |  |  |  |  |  |  |
| 2 | transcript:Vitvi08g01716\_t002 |  | \| \| |  | \| \| |  |  |  |  |  |  |  |  |
| 2 | transcript:Vitvi08g01717\_t001 |  | \| \| |  | \| \| |  |  |  |  |  |  |  |  |
| 2 | transcript:Vitvi08g01718\_t001 |  | \| \| |  | \| \| |  |  |  |  |  |  |  |  |
| 2 | transcript:Vitvi08g01719\_t001 |  | \| \| |  | \| \| |  |  |  |  |  |  |  |  |
| 2 | transcript:Vitvi08g01720\_t001 |  | \| \| |  | \| \| |  |  |  |  |  |  |  |  |
| 2 | transcript:Vitvi08g02342\_t001 |  | transcript:Vitvi06g01615\_t001 |  | \| \| |  |  |  |  |  |  |  |  |
| 2 | transcript:Vitvi08g04391\_t001 |  | \| \| |  | \| \| |  |  |  |  |  |  |  |  |
| 2 | transcript:Vitvi08g01721\_t001 |  | \| \| |  | \| \| |  |  |  |  |  |  |  |  |
| 2 | transcript:Vitvi08g01722\_t001 |  | \| \| |  | \| \| |  |  |  |  |  |  |  |  |
| 2 | transcript:Vitvi08g01723\_t002 |  | \| \| |  | transcript:Vitvi13g02568\_t001 |  |  |  |  |  |  |  |  |
| 2 | transcript:Vitvi08g01724\_t001 |  | \| \| |  | \| \| |  |  |  |  |  |  |  |  |
| 2 | transcript:Vitvi08g04392\_t001 |  | \| \| |  | \| \| |  |  |  |  |  |  |  |  |
| 2 | transcript:Vitvi08g01725\_t001 |  | transcript:Vitvi06g00130\_t001 |  | transcript:Vitvi13g01816\_t001 |  |  |  |  |  |  |  |  |
| 2 | transcript:Vitvi08g04393\_t001 |  | \| \| |  | \| \| |  |  |  |  |  |  |  |  |
| 2 | transcript:Vitvi08g01728\_t001 |  | \| \| |  | transcript:Vitvi13g01818\_t001 |  |  |  |  |  |  |  |  |
| 2 | transcript:Vitvi08g04394\_t001 |  | \| \| |  | \| \| |  |  |  |  |  |  |  |  |
| 2 | transcript:Vitvi08g01729\_t001 |  | \| \| |  | \| \| |  |  |  |  |  |  |  |  |
| 2 | transcript:Vitvi08g01730\_t001 |  | transcript:Vitvi06g00126\_t001 |  | \| \| |  |  |  |  |  |  |  |  |
| 2 | transcript:Vitvi08g01731\_t001 |  | transcript:Vitvi06g00125\_t001 |  | \| \| |  |  |  |  |  |  |  |  |
| 2 | transcript:Vitvi08g01732\_t001 |  | transcript:Vitvi06g00123\_t001 |  | transcript:Vitvi13g01819\_t001 |  |  |  |  |  |  |  |  |
| 2 | transcript:Vitvi08g04395\_t001 |  | \| \| |  | \| \| |  |  |  |  |  |  |  |  |
| 2 | transcript:Vitvi08g01733\_t001 |  | \| \| |  | \| \| |  |  |  |  |  |  |  |  |
| 2 | transcript:Vitvi08g02343\_t001 |  | \| \| |  | \| \| |  |  |  |  |  |  |  |  |
| 2 | transcript:Vitvi08g02344\_t001 |  | \| \| |  | \| \| |  |  |  |  |  |  |  |  |
| 2 | transcript:Vitvi08g01734\_t001 |  | \| \| |  | \| \| |  |  |  |  |  |  |  |  |
| 2 | transcript:Vitvi08g04396\_t001 |  | \| \| |  | \| \| |  |  |  |  |  |  |  |  |
| 2 | transcript:Vitvi08g02345\_t001 |  | transcript:Vitvi06g00119\_t001 |  | \| \| |  |  |  |  |  |  |  |  |
| 2 | transcript:Vitvi08g01735\_t001 |  | \| \| |  | \| \| |  |  |  |  |  |  |  |  |
| 2 | transcript:Vitvi08g01736\_t001 |  | \| \| |  | transcript:Vitvi13g02571\_t001 |  |  |  |  |  |  |  |  |
| 1 | transcript:Vitvi08g02346\_t001 |  | \| \| |  |  |  |  |  |  |  |  |  |  |
| 1 | transcript:Vitvi08g01737\_t002 |  | \| \| |  |  |  |  |  |  |  |  |  |  |
| 1 | transcript:Vitvi08g01738\_t001 |  | \| \| |  |  |  |  |  |  |  |  |  |  |
| 1 | transcript:Vitvi08g01739\_t002 |  | \| \| |  |  |  |  |  |  |  |  |  |  |
| 1 | transcript:Vitvi08g01740\_t001 |  | \| \| |  |  |  |  |  |  |  |  |  |  |
| 1 | transcript:Vitvi08g01741\_t001 |  | \| \| |  |  |  |  |  |  |  |  |  |  |
| 1 | transcript:Vitvi08g01742\_t001 |  | \| \| |  |  |  |  |  |  |  |  |  |  |
| 1 | transcript:Vitvi08g01743\_t001 |  | \| \| |  |  |  |  |  |  |  |  |  |  |
| 1 | transcript:Vitvi08g01744\_t001 |  | \| \| |  |  |  |  |  |  |  |  |  |  |
| 1 | transcript:Vitvi08g02347\_t001 |  | \| \| |  |  |  |  |  |  |  |  |  |  |
| 1 | transcript:Vitvi08g04397\_t001 |  | \| \| |  |  |  |  |  |  |  |  |  |  |
| 1 | transcript:Vitvi08g04398\_t001 |  | \| \| |  |  |  |  |  |  |  |  |  |  |
| 1 | transcript:Vitvi08g01746\_t001 |  | \| \| |  |  |  |  |  |  |  |  |  |  |
| 1 | transcript:Vitvi08g04399\_t001 |  | \| \| |  |  |  |  |  |  |  |  |  |  |
| 1 | transcript:Vitvi08g01747\_t001 |  | \| \| |  |  |  |  |  |  |  |  |  |  |
| 1 | transcript:Vitvi08g02349\_t001 |  | transcript:Vitvi06g00116\_t001 |  |  |  |  |  |  |  |  |  |  |
| 1 | transcript:Vitvi08g02350\_t001 |  | \| \| |  |  |  |  |  |  |  |  |  |  |
| 1 | transcript:Vitvi08g01748\_t001 |  | \| \| |  |  |  |  |  |  |  |  |  |  |
| 1 | transcript:Vitvi08g01749\_t001 |  | \| \| |  |  |  |  |  |  |  |  |  |  |
| 1 | transcript:Vitvi08g01750\_t001 |  | transcript:Vitvi06g00111\_t001 |  |  |  |  |  |  |  |  |  |  |
| 1 | transcript:Vitvi08g01751\_t001 |  | \| \| |  |  |  |  |  |  |  |  |  |  |
| 1 | transcript:Vitvi08g01752\_t001 |  | \| \| |  |  |  |  |  |  |  |  |  |  |
| 1 | transcript:Vitvi08g01753\_t001 |  | \| \| |  |  |  |  |  |  |  |  |  |  |
| 1 | transcript:Vitvi08g02352\_t002 |  | \| \| |  |  |  |  |  |  |  |  |  |  |
| 1 | transcript:Vitvi08g01756\_t001 |  | \| \| |  |  |  |  |  |  |  |  |  |  |
| 1 | transcript:Vitvi08g01757\_t001 |  | \| \| |  |  |  |  |  |  |  |  |  |  |
| 1 | transcript:Vitvi08g01758\_t001 |  | \| \| |  |  |  |  |  |  |  |  |  |  |
| 1 | transcript:Vitvi08g01759\_t001 |  | \| \| |  |  |  |  |  |  |  |  |  |  |
| 1 | transcript:Vitvi08g04400\_t001 |  | \| \| |  |  |  |  |  |  |  |  |  |  |
| 1 | transcript:Vitvi08g01760\_t001 |  | transcript:Vitvi06g00108\_t002 |  |  |  |  |  |  |  |  |  |  |
| 1 | transcript:Vitvi08g01761\_t001 |  | \| \| |  |  |  |  |  |  |  |  |  |  |
| 1 | transcript:Vitvi08g04401\_t001 |  | \| \| |  |  |  |  |  |  |  |  |  |  |
| 1 | transcript:Vitvi08g01762\_t001 |  | transcript:Vitvi06g00106\_t001 |  |  |  |  |  |  |  |  |  |  |
| 1 | transcript:Vitvi08g01764\_t001 |  | \| \| |  |  |  |  |  |  |  |  |  |  |
| 1 | transcript:Vitvi08g01765\_t001 |  | \| \| |  |  |  |  |  |  |  |  |  |  |
| 1 | transcript:Vitvi08g01766\_t001 |  | \| \| |  |  |  |  |  |  |  |  |  |  |
| 1 | transcript:Vitvi08g01767\_t001 |  | transcript:Vitvi06g00102\_t001 |  |  |  |  |  |  |  |  |  |  |
| 0 | transcript:Vitvi08g04402\_t001 |  |  |  |  |  |  |  |  |  |  |  |  |
| 0 | transcript:Vitvi08g04403\_t001 |  |  |  |  |  |  |  |  |  |  |  |  |
| 0 | transcript:Vitvi08g01768\_t001 |  |  |  |  |  |  |  |  |  |  |  |  |
| 0 | transcript:Vitvi08g04404\_t001 |  |  |  |  |  |  |  |  |  |  |  |  |
| 0 | transcript:Vitvi08g01769\_t001 |  |  |  |  |  |  |  |  |  |  |  |  |
| 0 | transcript:Vitvi08g04405\_t001 |  |  |  |  |  |  |  |  |  |  |  |  |
| 0 | transcript:Vitvi08g01770\_t001 |  |  |  |  |  |  |  |  |  |  |  |  |
| 0 | transcript:Vitvi08g01771\_t001 |  |  |  |  |  |  |  |  |  |  |  |  |
| 0 | transcript:Vitvi08g01772\_t002 |  |  |  |  |  |  |  |  |  |  |  |  |
| 0 | transcript:Vitvi08g01773\_t001 |  |  |  |  |  |  |  |  |  |  |  |  |
| 0 | transcript:Vitvi08g01774\_t001 |  |  |  |  |  |  |  |  |  |  |  |  |
| 0 | transcript:Vitvi08g02355\_t001 |  |  |  |  |  |  |  |  |  |  |  |  |
| 0 | transcript:Vitvi08g02357\_t001 |  |  |  |  |  |  |  |  |  |  |  |  |
| 0 | transcript:Vitvi08g02358\_t001 |  |  |  |  |  |  |  |  |  |  |  |  |
| 0 | transcript:Vitvi08g02359\_t001 |  |  |  |  |  |  |  |  |  |  |  |  |
| 0 | transcript:Vitvi08g04406\_t001 |  |  |  |  |  |  |  |  |  |  |  |  |
| 0 | transcript:Vitvi08g02360\_t001 |  |  |  |  |  |  |  |  |  |  |  |  |
| 0 | transcript:Vitvi08g04407\_t001 |  |  |  |  |  |  |  |  |  |  |  |  |
| 0 | transcript:Vitvi08g01776\_t001 |  |  |  |  |  |  |  |  |  |  |  |  |
| 0 | transcript:Vitvi08g04408\_t001 |  |  |  |  |  |  |  |  |  |  |  |  |
| 0 | transcript:Vitvi08g01777\_t001 |  |  |  |  |  |  |  |  |  |  |  |  |
| 0 | transcript:Vitvi08g01778\_t001 |  |  |  |  |  |  |  |  |  |  |  |  |
| 0 | transcript:Vitvi08g02363\_t004 |  |  |  |  |  |  |  |  |  |  |  |  |
| 0 | transcript:Vitvi08g01779\_t002 |  |  |  |  |  |  |  |  |  |  |  |  |
| 1 | transcript:Vitvi08g01780\_t001 |  | transcript:Vitvi06g04028\_t001 |  |  |  |  |  |  |  |  |  |  |
| 1 | transcript:Vitvi08g01781\_t001 |  | \| \| |  |  |  |  |  |  |  |  |  |  |
| 1 | transcript:Vitvi08g01782\_t001 |  | transcript:Vitvi06g00086\_t003 |  |  |  |  |  |  |  |  |  |  |
| 1 | transcript:Vitvi08g01783\_t001 |  | \| \| |  |  |  |  |  |  |  |  |  |  |
| 1 | transcript:Vitvi08g01784\_t001 |  | \| \| |  |  |  |  |  |  |  |  |  |  |
| 1 | transcript:Vitvi08g01786\_t001 |  | \| \| |  |  |  |  |  |  |  |  |  |  |
| 1 | transcript:Vitvi08g02366\_t001 |  | \| \| |  |  |  |  |  |  |  |  |  |  |
| 1 | transcript:Vitvi08g01787\_t001 |  | \| \| |  |  |  |  |  |  |  |  |  |  |
| 1 | transcript:Vitvi08g01788\_t001 |  | \| \| |  |  |  |  |  |  |  |  |  |  |
| 1 | transcript:Vitvi08g01789\_t001 |  | \| \| |  |  |  |  |  |  |  |  |  |  |
| 1 | transcript:Vitvi08g01790\_t001 |  | \| \| |  |  |  |  |  |  |  |  |  |  |
| 1 | transcript:Vitvi08g01791\_t002 |  | \| \| |  |  |  |  |  |  |  |  |  |  |
| 1 | transcript:Vitvi08g01792\_t002 |  | \| \| |  |  |  |  |  |  |  |  |  |  |
| 1 | transcript:Vitvi08g01793\_t003 |  | \| \| |  |  |  |  |  |  |  |  |  |  |
| 1 | transcript:Vitvi08g01794\_t001 |  | transcript:Vitvi06g00071\_t001 |  |  |  |  |  |  |  |  |  |  |
| 1 | transcript:Vitvi08g04409\_t001 |  | \| \| |  |  |  |  |  |  |  |  |  |  |
| 1 | transcript:Vitvi08g01795\_t001 |  | \| \| |  |  |  |  |  |  |  |  |  |  |
| 1 | transcript:Vitvi08g02367\_t001 |  | \| \| |  |  |  |  |  |  |  |  |  |  |
| 1 | transcript:Vitvi08g04410\_t001 |  | \| \| |  |  |  |  |  |  |  |  |  |  |
| 1 | transcript:Vitvi08g01796\_t002 |  | \| \| |  |  |  |  |  |  |  |  |  |  |
| 1 | transcript:Vitvi08g01797\_t001 |  | transcript:Vitvi06g00059\_t001 |  |  |  |  |  |  |  |  |  |  |
| 1 | transcript:Vitvi08g01798\_t001 |  | transcript:Vitvi06g00053\_t001 |  |  |  |  |  |  |  |  |  |  |
| 1 | transcript:Vitvi08g01965\_t001 |  | transcript:Vitvi06g00052\_t001 |  |  |  |  |  |  |  |  |  |  |
| 1 | transcript:Vitvi08g01800\_t001 |  | transcript:Vitvi06g00051\_t002 |  |  |  |  |  |  |  |  |  |  |
| 1 | transcript:Vitvi08g04411\_t001 |  | \| \| |  |  |  |  |  |  |  |  |  |  |
| 1 | transcript:Vitvi08g01802\_t001 |  | \| \| |  |  |  |  |  |  |  |  |  |  |
| 1 | transcript:Vitvi08g01803\_t001 |  | \| \| |  |  |  |  |  |  |  |  |  |  |
| 1 | transcript:Vitvi08g01804\_t001 |  | \| \| |  |  |  |  |  |  |  |  |  |  |
| 1 | transcript:Vitvi08g01805\_t001 |  | \| \| |  |  |  |  |  |  |  |  |  |  |
| 1 | transcript:Vitvi08g02368\_t001 |  | \| \| |  |  |  |  |  |  |  |  |  |  |
| 1 | transcript:Vitvi08g01807\_t001 |  | transcript:Vitvi06g00046\_t002 |  |  |  |  |  |  |  |  |  |  |
| 1 | transcript:Vitvi08g01808\_t002 |  | \| \| |  |  |  |  |  |  |  |  |  |  |
| 1 | transcript:Vitvi08g02369\_t001 |  | transcript:Vitvi06g01582\_t001 |  |  |  |  |  |  |  |  |  |  |
| 1 | transcript:Vitvi08g01809\_t001 |  | \| \| |  |  |  |  |  |  |  |  |  |  |
| 1 | transcript:Vitvi08g01810\_t001 |  | \| \| |  |  |  |  |  |  |  |  |  |  |
| 1 | transcript:Vitvi08g01811\_t001 |  | \| \| |  |  |  |  |  |  |  |  |  |  |
| 1 | transcript:Vitvi08g01812\_t002 |  | \| \| |  |  |  |  |  |  |  |  |  |  |
| 1 | transcript:Vitvi08g01813\_t001 |  | \| \| |  |  |  |  |  |  |  |  |  |  |
| 1 | transcript:Vitvi08g01814\_t001 |  | \| \| |  |  |  |  |  |  |  |  |  |  |
| 1 | transcript:Vitvi08g01815\_t001 |  | \| \| |  |  |  |  |  |  |  |  |  |  |
| 1 | transcript:Vitvi08g01816\_t001 |  | transcript:Vitvi06g01575\_t001 |  |  |  |  |  |  |  |  |  |  |
| 1 | transcript:Vitvi08g01817\_t003 |  | transcript:Vitvi06g00029\_t001 |  |  |  |  |  |  |  |  |  |  |
| 1 | transcript:Vitvi08g01818\_t001 |  | \| \| |  |  |  |  |  |  |  |  |  |  |
| 1 | transcript:Vitvi08g04412\_t001 |  | \| \| |  |  |  |  |  |  |  |  |  |  |
| 1 | transcript:Vitvi08g01820\_t001 |  | \| \| |  |  |  |  |  |  |  |  |  |  |
| 1 | transcript:Vitvi08g01821\_t001 |  | \| \| |  |  |  |  |  |  |  |  |  |  |
| 1 | transcript:Vitvi08g01822\_t001 |  | \| \| |  |  |  |  |  |  |  |  |  |  |
| 1 | transcript:Vitvi08g01824\_t001 |  | \| \| |  |  |  |  |  |  |  |  |  |  |
| 1 | transcript:Vitvi08g01825\_t001 |  | \| \| |  |  |  |  |  |  |  |  |  |  |
| 1 | transcript:Vitvi08g01826\_t001 |  | \| \| |  |  |  |  |  |  |  |  |  |  |
| 1 | transcript:Vitvi08g01828\_t001 |  | \| \| |  |  |  |  |  |  |  |  |  |  |
| 1 | transcript:Vitvi08g01829\_t001 |  | \| \| |  |  |  |  |  |  |  |  |  |  |
| 1 | transcript:Vitvi08g01831\_t001 |  | \| \| |  |  |  |  |  |  |  |  |  |  |
| 1 | transcript:Vitvi08g01832\_t001 |  | \| \| |  |  |  |  |  |  |  |  |  |  |
| 1 | transcript:Vitvi08g01833\_t004 |  | \| \| |  |  |  |  |  |  |  |  |  |  |
| 1 | transcript:Vitvi08g01834\_t001 |  | transcript:Vitvi06g00024\_t001 |  |  |  |  |  |  |  |  |  |  |
| 1 | transcript:Vitvi08g01835\_t001 |  | \| \| |  |  |  |  |  |  |  |  |  |  |
| 1 | transcript:Vitvi08g01836\_t001 |  | transcript:Vitvi06g00021\_t001 |  |  |  |  |  |  |  |  |  |  |
| 0 | transcript:Vitvi08g01837\_t001 |  |  |  |  |  |  |  |  |  |  |  |  |
| 0 | transcript:Vitvi08g01838\_t001 |  |  |  |  |  |  |  |  |  |  |  |  |
| 0 | transcript:Vitvi08g01839\_t001 |  |  |  |  |  |  |  |  |  |  |  |  |
| 0 | transcript:Vitvi08g01841\_t001 |  |  |  |  |  |  |  |  |  |  |  |  |
| 0 | transcript:Vitvi08g02370\_t001 |  |  |  |  |  |  |  |  |  |  |  |  |
| 0 | transcript:Vitvi08g01842\_t001 |  |  |  |  |  |  |  |  |  |  |  |  |
| 0 | transcript:Vitvi08g01843\_t001 |  |  |  |  |  |  |  |  |  |  |  |  |
| 0 | transcript:Vitvi08g02371\_t001 |  |  |  |  |  |  |  |  |  |  |  |  |
| 1 | transcript:Vitvi08g01844\_t001 |  | transcript:Vitvi13g01771\_t001 |  |  |  |  |  |  |  |  |  |  |
| 1 | transcript:Vitvi08g01845\_t001 |  | \| \| |  |  |  |  |  |  |  |  |  |  |
| 1 | transcript:Vitvi08g02372\_t001 |  | transcript:Vitvi13g01766\_t001 |  |  |  |  |  |  |  |  |  |  |
| 1 | transcript:Vitvi08g01846\_t001 |  | \| \| |  |  |  |  |  |  |  |  |  |  |
| 1 | transcript:Vitvi08g01847\_t001 |  | \| \| |  |  |  |  |  |  |  |  |  |  |
| 1 | transcript:Vitvi08g01848\_t001 |  | \| \| |  |  |  |  |  |  |  |  |  |  |
| 1 | transcript:Vitvi08g04413\_t001 |  | \| \| |  |  |  |  |  |  |  |  |  |  |
| 1 | transcript:Vitvi08g02373\_t001 |  | \| \| |  |  |  |  |  |  |  |  |  |  |
| 1 | transcript:Vitvi08g02374\_t001 |  | \| \| |  |  |  |  |  |  |  |  |  |  |
| 1 | transcript:Vitvi08g02375\_t001 |  | \| \| |  |  |  |  |  |  |  |  |  |  |
| 1 | transcript:Vitvi08g01851\_t001 |  | \| \| |  |  |  |  |  |  |  |  |  |  |
| 1 | transcript:Vitvi08g02377\_t001 |  | transcript:Vitvi13g01765\_t001 |  |  |  |  |  |  |  |  |  |  |
| 2 | transcript:Vitvi08g01852\_t001 |  | transcript:Vitvi13g01764\_t001 |  | transcript:Vitvi06g00181\_t001 |  |  |  |  |  |  |  |  |
| 2 | transcript:Vitvi08g01853\_t001 |  | \| \| |  | \| \| |  |  |  |  |  |  |  |  |
| 2 | transcript:Vitvi08g01854\_t001 |  | \| \| |  | \| \| |  |  |  |  |  |  |  |  |
| 2 | transcript:Vitvi08g01855\_t001 |  | \| \| |  | \| \| |  |  |  |  |  |  |  |  |
| 2 | transcript:Vitvi08g02378\_t004 |  | \| \| |  | \| \| |  |  |  |  |  |  |  |  |
| 2 | transcript:Vitvi08g01856\_t001 |  | \| \| |  | \| \| |  |  |  |  |  |  |  |  |
| 2 | transcript:Vitvi08g01857\_t001 |  | \| \| |  | \| \| |  |  |  |  |  |  |  |  |
| 2 | transcript:Vitvi08g01858\_t001 |  | \| \| |  | \| \| |  |  |  |  |  |  |  |  |
| 2 | transcript:Vitvi08g01859\_t001 |  | \| \| |  | \| \| |  |  |  |  |  |  |  |  |
| 2 | transcript:Vitvi08g01860\_t003 |  | \| \| |  | \| \| |  |  |  |  |  |  |  |  |
| 2 | transcript:Vitvi08g01861\_t001 |  | \| \| |  | transcript:Vitvi06g00187\_t001 |  |  |  |  |  |  |  |  |
| 2 | transcript:Vitvi08g01863\_t001 |  | \| \| |  | \| \| |  |  |  |  |  |  |  |  |
| 2 | transcript:Vitvi08g04414\_t001 |  | \| \| |  | \| \| |  |  |  |  |  |  |  |  |
| 2 | transcript:Vitvi08g01864\_t001 |  | transcript:Vitvi13g01755\_t001 |  | transcript:Vitvi06g00189\_t003 |  |  |  |  |  |  |  |  |
| 2 | transcript:Vitvi08g04415\_t001 |  | \| \| |  | \| \| |  |  |  |  |  |  |  |  |
| 2 | transcript:Vitvi08g01865\_t001 |  | \| \| |  | \| \| |  |  |  |  |  |  |  |  |
| 2 | transcript:Vitvi08g01866\_t002 |  | \| \| |  | transcript:Vitvi06g00191\_t002 |  |  |  |  |  |  |  |  |
| 2 | transcript:Vitvi08g02381\_t001 |  | transcript:Vitvi13g02516\_t001 |  | transcript:Vitvi06g04056\_t001 |  |  |  |  |  |  |  |  |
| 1 | transcript:Vitvi08g04416\_t001 |  |  |  | \| \| |  |  |  |  |  |  |  |  |
| 1 | transcript:Vitvi08g04417\_t001 |  |  |  | \| \| |  |  |  |  |  |  |  |  |
| 2 | transcript:Vitvi08g02382\_t001 |  | transcript:Vitvi13g02508\_t001 |  | transcript:Vitvi06g04057\_t001 |  |  |  |  |  |  |  |  |
| 2 | transcript:Vitvi08g02383\_t001 |  | \| \| |  | \| \| |  |  |  |  |  |  |  |  |
| 2 | transcript:Vitvi08g04418\_t001 |  | \| \| |  | \| \| |  |  |  |  |  |  |  |  |
| 2 | transcript:Vitvi08g04419\_t001 |  | \| \| |  | \| \| |  |  |  |  |  |  |  |  |
| 2 | transcript:Vitvi08g04420\_t001 |  | \| \| |  | \| \| |  |  |  |  |  |  |  |  |
| 2 | transcript:Vitvi08g04421\_t001 |  | \| \| |  | \| \| |  |  |  |  |  |  |  |  |
| 2 | transcript:Vitvi08g02386\_t001 |  | \| \| |  | \| \| |  |  |  |  |  |  |  |  |
| 2 | transcript:Vitvi08g02387\_t001 |  | \| \| |  | \| \| |  |  |  |  |  |  |  |  |
| 2 | transcript:Vitvi08g01867\_t001 |  | \| \| |  | \| \| |  |  |  |  |  |  |  |  |
| 2 | transcript:Vitvi08g01868\_t001 |  | \| \| |  | \| \| |  |  |  |  |  |  |  |  |
| 2 | transcript:Vitvi08g01869\_t001 |  | transcript:Vitvi13g01748\_t001 |  | transcript:Vitvi06g00195\_t001 |  |  |  |  |  |  |  |  |
| 1 | transcript:Vitvi08g01870\_t002 |  | \| \| |  |  |  |  |  |  |  |  |  |  |
| 1 | transcript:Vitvi08g01871\_t001 |  | \| \| |  |  |  |  |  |  |  |  |  |  |
| 1 | transcript:Vitvi08g04422\_t001 |  | \| \| |  |  |  |  |  |  |  |  |  |  |
| 1 | transcript:Vitvi08g02389\_t001 |  | \| \| |  |  |  |  |  |  |  |  |  |  |
| 1 | transcript:Vitvi08g01872\_t001 |  | \| \| |  |  |  |  |  |  |  |  |  |  |
| 1 | transcript:Vitvi08g01873\_t001 |  | \| \| |  |  |  |  |  |  |  |  |  |  |
| 1 | transcript:Vitvi08g01874\_t001 |  | \| \| |  |  |  |  |  |  |  |  |  |  |
| 1 | transcript:Vitvi08g02390\_t001 |  | \| \| |  |  |  |  |  |  |  |  |  |  |
| 1 | transcript:Vitvi08g04423\_t001 |  | \| \| |  |  |  |  |  |  |  |  |  |  |
| 1 | transcript:Vitvi08g04424\_t001 |  | \| \| |  |  |  |  |  |  |  |  |  |  |
| 1 | transcript:Vitvi08g01966\_t001 |  | \| \| |  |  |  |  |  |  |  |  |  |  |
| 1 | transcript:Vitvi08g01875\_t001 |  | \| \| |  |  |  |  |  |  |  |  |  |  |
| 1 | transcript:Vitvi08g02391\_t001 |  | \| \| |  |  |  |  |  |  |  |  |  |  |
| 1 | transcript:Vitvi08g01876\_t002 |  | \| \| |  |  |  |  |  |  |  |  |  |  |
| 1 | transcript:Vitvi08g01878\_t001 |  | \| \| |  |  |  |  |  |  |  |  |  |  |
| 1 | transcript:Vitvi08g01879\_t001 |  | transcript:Vitvi13g01734\_t001 |  |  |  |  |  |  |  |  |  |  |
| 1 | transcript:Vitvi08g01880\_t002 |  | transcript:Vitvi13g01732\_t001 |  |  |  |  |  |  |  |  |  |  |
| 1 | transcript:Vitvi08g01881\_t001 |  | \| \| |  |  |  |  |  |  |  |  |  |  |
| 1 | transcript:Vitvi08g02393\_t001 |  | \| \| |  |  |  |  |  |  |  |  |  |  |
| 1 | transcript:Vitvi08g01883\_t002 |  | transcript:Vitvi13g01729\_t002 |  |  |  |  |  |  |  |  |  |  |
| 1 | transcript:Vitvi08g01884\_t001 |  | \| \| |  |  |  |  |  |  |  |  |  |  |
| 1 | transcript:Vitvi08g01886\_t001 |  | \| \| |  |  |  |  |  |  |  |  |  |  |
| 1 | transcript:Vitvi08g01887\_t001 |  | \| \| |  |  |  |  |  |  |  |  |  |  |
| 1 | transcript:Vitvi08g01888\_t001 |  | \| \| |  |  |  |  |  |  |  |  |  |  |
| 1 | transcript:Vitvi08g01889\_t002 |  | \| \| |  |  |  |  |  |  |  |  |  |  |
| 1 | transcript:Vitvi08g01890\_t003 |  | \| \| |  |  |  |  |  |  |  |  |  |  |
| 1 | transcript:Vitvi08g01891\_t001 |  | \| \| |  |  |  |  |  |  |  |  |  |  |
| 1 | transcript:Vitvi08g02394\_t001 |  | transcript:Vitvi13g02493\_t001 |  |  |  |  |  |  |  |  |  |  |
| 1 | transcript:Vitvi08g01893\_t001 |  | transcript:Vitvi13g01722\_t001 |  |  |  |  |  |  |  |  |  |  |
| 1 | transcript:Vitvi08g01894\_t001 |  | transcript:Vitvi13g01703\_t001 |  |  |  |  |  |  |  |  |  |  |
| 1 | transcript:Vitvi08g01895\_t001 |  | \| \| |  |  |  |  |  |  |  |  |  |  |
| 1 | transcript:Vitvi08g01896\_t001 |  | \| \| |  |  |  |  |  |  |  |  |  |  |
| 1 | transcript:Vitvi08g02395\_t001 |  | \| \| |  |  |  |  |  |  |  |  |  |  |
| 1 | transcript:Vitvi08g01897\_t001 |  | \| \| |  |  |  |  |  |  |  |  |  |  |
| 1 | transcript:Vitvi08g04425\_t001 |  | \| \| |  |  |  |  |  |  |  |  |  |  |
| 1 | transcript:Vitvi08g01899\_t001 |  | \| \| |  |  |  |  |  |  |  |  |  |  |
| 1 | transcript:Vitvi08g01900\_t001 |  | \| \| |  |  |  |  |  |  |  |  |  |  |
| 1 | transcript:Vitvi08g02397\_t001 |  | \| \| |  |  |  |  |  |  |  |  |  |  |
| 1 | transcript:Vitvi08g01901\_t001 |  | \| \| |  |  |  |  |  |  |  |  |  |  |
| 1 | transcript:Vitvi08g02398\_t001 |  | \| \| |  |  |  |  |  |  |  |  |  |  |
| 1 | transcript:Vitvi08g01902\_t001 |  | \| \| |  |  |  |  |  |  |  |  |  |  |
| 1 | transcript:Vitvi08g04426\_t001 |  | \| \| |  |  |  |  |  |  |  |  |  |  |
| 1 | transcript:Vitvi08g01904\_t001 |  | transcript:Vitvi13g01686\_t002 |  |  |  |  |  |  |  |  |  |  |
| 1 | transcript:Vitvi08g01905\_t002 |  | transcript:Vitvi13g01684\_t001 |  |  |  |  |  |  |  |  |  |  |
| 1 | transcript:Vitvi08g01906\_t001 |  | \| \| |  |  |  |  |  |  |  |  |  |  |
| 1 | transcript:Vitvi08g01910\_t001 |  | \| \| |  |  |  |  |  |  |  |  |  |  |
| 1 | transcript:Vitvi08g01911\_t001 |  | \| \| |  |  |  |  |  |  |  |  |  |  |
| 1 | transcript:Vitvi08g01912\_t001 |  | \| \| |  |  |  |  |  |  |  |  |  |  |
| 1 | transcript:Vitvi08g04427\_t001 |  | \| \| |  |  |  |  |  |  |  |  |  |  |
| 1 | transcript:Vitvi08g02401\_t001 |  | \| \| |  |  |  |  |  |  |  |  |  |  |
| 1 | transcript:Vitvi08g01913\_t001 |  | \| \| |  |  |  |  |  |  |  |  |  |  |
| 1 | transcript:Vitvi08g01914\_t001 |  | transcript:Vitvi13g01672\_t001 |  |  |  |  |  |  |  |  |  |  |
| 0 | transcript:Vitvi08g01915\_t001 |  |  |  |  |  |  |  |  |  |  |  |  |
| 0 | transcript:Vitvi08g01916\_t002 |  |  |  |  |  |  |  |  |  |  |  |  |
| 0 | transcript:Vitvi08g01917\_t001 |  |  |  |  |  |  |  |  |  |  |  |  |
| 0 | transcript:Vitvi08g01918\_t001 |  |  |  |  |  |  |  |  |  |  |  |  |
| 0 | transcript:Vitvi08g01919\_t001 |  |  |  |  |  |  |  |  |  |  |  |  |
| 0 | transcript:Vitvi08g01920\_t001 |  |  |  |  |  |  |  |  |  |  |  |  |
| 0 | transcript:Vitvi08g04428\_t001 |  |  |  |  |  |  |  |  |  |  |  |  |
| 0 | transcript:Vitvi08g01922\_t001 |  |  |  |  |  |  |  |  |  |  |  |  |
| 0 | transcript:Vitvi08g02406\_t001 |  |  |  |  |  |  |  |  |  |  |  |  |
| 0 | transcript:Vitvi08g01923\_t001 |  |  |  |  |  |  |  |  |  |  |  |  |
| 0 | transcript:Vitvi08g01924\_t001 |  |  |  |  |  |  |  |  |  |  |  |  |
| 0 | transcript:Vitvi08g01925\_t001 |  |  |  |  |  |  |  |  |  |  |  |  |
| 0 | transcript:Vitvi08g01926\_t001 |  |  |  |  |  |  |  |  |  |  |  |  |
| 0 | transcript:Vitvi08g01927\_t001 |  |  |  |  |  |  |  |  |  |  |  |  |
| 0 | transcript:Vitvi08g01929\_t001 |  |  |  |  |  |  |  |  |  |  |  |  |
| 0 | transcript:Vitvi08g01931\_t001 |  |  |  |  |  |  |  |  |  |  |  |  |
| 0 | transcript:Vitvi08g01932\_t001 |  |  |  |  |  |  |  |  |  |  |  |  |
| 0 | transcript:Vitvi08g01933\_t001 |  |  |  |  |  |  |  |  |  |  |  |  |
| 0 | transcript:Vitvi08g01934\_t001 |  |  |  |  |  |  |  |  |  |  |  |  |
| 0 | transcript:Vitvi08g01935\_t001 |  |  |  |  |  |  |  |  |  |  |  |  |
| 0 | transcript:Vitvi08g01936\_t001 |  |  |  |  |  |  |  |  |  |  |  |  |
| 0 | transcript:Vitvi08g01938\_t001 |  |  |  |  |  |  |  |  |  |  |  |  |
| 0 | transcript:Vitvi08g01939\_t001 |  |  |  |  |  |  |  |  |  |  |  |  |
| 0 | transcript:Vitvi08g01940\_t001 |  |  |  |  |  |  |  |  |  |  |  |  |
| 0 | transcript:Vitvi08g01943\_t001 |  |  |  |  |  |  |  |  |  |  |  |  |
| 0 | transcript:Vitvi08g02408\_t001 |  |  |  |  |  |  |  |  |  |  |  |  |
| 0 | transcript:Vitvi08g02410\_t001 |  |  |  |  |  |  |  |  |  |  |  |  |
| 0 | transcript:Vitvi08g04429\_t001 |  |  |  |  |  |  |  |  |  |  |  |  |
| 0 | transcript:Vitvi08g02411\_t001 |  |  |  |  |  |  |  |  |  |  |  |  |
| 0 | transcript:Vitvi08g02412\_t001 |  |  |  |  |  |  |  |  |  |  |  |  |
| 0 | transcript:Vitvi08g01944\_t001 |  |  |  |  |  |  |  |  |  |  |  |  |
| 0 | transcript:Vitvi08g04430\_t001 |  |  |  |  |  |  |  |  |  |  |  |  |
| 0 | transcript:Vitvi08g01945\_t001 |  |  |  |  |  |  |  |  |  |  |  |  |
| 0 | transcript:Vitvi08g01946\_t001 |  |  |  |  |  |  |  |  |  |  |  |  |
| 0 | transcript:Vitvi08g01947\_t001 |  |  |  |  |  |  |  |  |  |  |  |  |
| 0 | transcript:Vitvi08g02413\_t001 |  |  |  |  |  |  |  |  |  |  |  |  |
| 0 | transcript:Vitvi08g01949\_t001 |  |  |  |  |  |  |  |  |  |  |  |  |
| 0 | transcript:Vitvi08g04431\_t001 |  |  |  |  |  |  |  |  |  |  |  |  |
| 0 | transcript:Vitvi08g02414\_t001 |  |  |  |  |  |  |  |  |  |  |  |  |
| 0 | transcript:Vitvi08g02415\_t001 |  |  |  |  |  |  |  |  |  |  |  |  |
| 0 | transcript:Vitvi08g01950\_t001 |  |  |  |  |  |  |  |  |  |  |  |  |
| 0 | transcript:Vitvi08g04432\_t001 |  |  |  |  |  |  |  |  |  |  |  |  |
| 0 | transcript:Vitvi08g01951\_t002 |  |  |  |  |  |  |  |  |  |  |  |  |
| 0 | transcript:Vitvi08g01952\_t001 |  |  |  |  |  |  |  |  |  |  |  |  |
| 0 | transcript:Vitvi08g01953\_t001 |  |  |  |  |  |  |  |  |  |  |  |  |
| 0 | transcript:Vitvi08g01964\_t001 |  |  |  |  |  |  |  |  |  |  |  |  |
| 0 | transcript:Vitvi08g01963\_t001 |  |  |  |  |  |  |  |  |  |  |  |  |
| 0 | transcript:Vitvi08g02419\_t001 |  |  |  |  |  |  |  |  |  |  |  |  |
| 0 | transcript:Vitvi08g01962\_t001 |  |  |  |  |  |  |  |  |  |  |  |  |
| 0 | transcript:Vitvi08g01960\_t001 |  |  |  |  |  |  |  |  |  |  |  |  |
| 0 | transcript:Vitvi08g01959\_t001 |  |  |  |  |  |  |  |  |  |  |  |  |
| 0 | transcript:Vitvi08g01958\_t001 |  |  |  |  |  |  |  |  |  |  |  |  |
| 0 | transcript:Vitvi08g02418\_t001 |  |  |  |  |  |  |  |  |  |  |  |  |
| 0 | transcript:Vitvi08g04433\_t001 |  |  |  |  |  |  |  |  |  |  |  |  |
| 0 | transcript:Vitvi08g01957\_t001 |  |  |  |  |  |  |  |  |  |  |  |  |
| 0 | transcript:Vitvi08g01955\_t001 |  |  |  |  |  |  |  |  |  |  |  |  |
